# Sustainable age-friendly cities and communities in China: a scoping review and narrative assessment of national policies

**DOI:** 10.1016/j.lanwpc.2025.101723

**Published:** 2025-11-07

**Authors:** Yanhui Jia, Siwon Lee, Mikiko Kanda, Pankyu Park, Sally J. Edwards, Jiuxuan Gao, Weiju Zhou, John S. Ji

**Affiliations:** a Vanke School of Public Health, Tsinghua University, Beijing, 100084, China; b World Health Organization (WHO) Regional Office for the Western Pacific, Manila, Philippines

**Keywords:** Healthy aging, Sustainable, Climate resilience, China, Policy, Scoping review

## Abstract

**Background:**

Climate change, aging populations, and rapid urbanization intersect to pose public health challenges in China, impacting the livelihoods of older adults. This review maps China's national policies shaping Sustainable Age-Friendly Cities and Communities (AFCC), highlights supporting sub-national innovations, and identifies potential policy gaps and opportunities.

**Methods:**

We searched 26 national government websites for policy documents published from January 1, 2020, to December 20, 2024, screening 35,809 records and including 125 that met criteria.

**Findings:**

National frameworks (National Strategy for Climate Change Adaptation 2035, Healthy China 2030, Healthy Cities) have parallel efforts linking climate resilience and healthy aging. However, policy domains are siloed: age-friendly policies focus on healthcare accessibility, whereas sustainability policies target ecological restoration. Sub-national innovations, such as 15-min life circles, sponge cities for flood, “one old, one young” inter-generational hubs, advance climate adaptation. The large-scale Three-North Shelter Forest Program indirectly benefits older adults respiratory risk by controlling dust and desertification. However, these initiatives run in parallel, with limited shared targets, data systems, and co-financing. Institutional fragmentation and regional disparities in geographic, economic, and administrative capacities impede uniform implementation, risking inefficient spending and missed synergies.

**Interpretation:**

Effective action on concurrent aging and climate risks hinges on policy-and-practice integration of AFCC and sustainability agendas. Age-friendly plans should include sustainability and vice versa. China's administrative grid and large-scale ecological programs provide infrastructure for delivery of dual-benefits.

**Funding:**

This work is supported by the World Health Organization (WPRO/2024-02/AGE-DHP/22552 4), the National Natural Science Foundation of China (82422064, 82250610230, 42307535, 42577488), the Natural Science Foundation of Beijing (IS23105), and the National Bureau for Disease Control and Prevention (20241660047).

## Introduction

China's rapid economic and technological development is closely tied to its large population and rapid urbanization both in cities and peri-urban areas. These dynamics introduce societal shifts, including population aging and urban sustainability challenges, posing both pressures and opportunities for public health. As of 2023, China has over 210 million people aged 65 and above (15.4% of the national population).^1^ With increasing life expectancy and declining fertility rates,^2^ the proportion of older individuals is expected to continue rising. By 2035, it is projected that the number of older people aged 60 and above will exceed 400 million, accounting for more than 30% of the total population, which indicates that China will formally enter a phase of severe population aging.^3^

Significant regional disparities exist, while the absolute number of older individuals is higher in urban compared to rural areas, the degree of aging is more pronounced in rural areas. This disparity is closely linked to rapid urbanization, urban sprawl, and population migration patterns. Younger individuals migrate to developed cities, resulting in higher concentrations of older adults in rural or less developed urban areas. This demographic distribution presents numerous challenges for older people, including limited access to healthcare, lack of inter-generational companionship, transportation difficulties, and inadequate nutritional intake, all of which adversely affect their physical and mental well-being.

Urbanization has brought significant changes to the built environment, with direct impacts for public health. China accounted for 47.5% of the total urban expansion worldwide between 2001 and 2018,^4^ which brings challenges such as resource scarcity, traffic congestion, environmental pollution, and ecological degradation. In the context of global climate change, urban development can modify local temperatures and alter the spatial distribution of precipitation, increasing the frequency of extreme weather and severe convective events.^5^^,^^6^ Rapid urbanization has also been shown to exacerbate warming from continental to regional scales.^7^ Enhancing urban climate resilience and ensuring sustainable development is critical to achieving the goals of a “Healthy China”.

Urban public health, environmental gerontology, and sustainable city design have converged on a shared problem: how the built environment, services, and governance shape the older adults’ exposure and adaptive capacity in a warming, urbanizing world. Older populations are facing compounded risks: frequent heatwaves elevate the mortality risk^8^; urban heat islands intensify chronic health conditions^9^; dense, high-rise urban form can impede evacuation and timely access to care during disasters.^10^^,^^11^ Furthermore, evidence has revealed that neighborhood walkability and transit, green space accessibility, barrier-free housing and public spaces, and access to primary care and social participation are associated with morbidity, mortality, and well-being in later life.^12^, ^13^, ^14^, ^15^

During the past few years, more countries have realized the significance of collaborative governance in addressing these challenges. The United Nations has reframed aging policy as an adaptation agenda, encouraging cities to integrate heat preparedness, universal design, and social care into mainstream planning.^16^ Japan has mainstreamed universal design and disaster-ready neighborhood improvements after major heat and flood events.^17^ Australia has also linked age-friendly planning guidelines with urban greening and climate-resilience strategies to deliver co-benefits for health and emissions reduction.^18^ At the same time, China's national strategies have increasingly addressed these issues, yet implementation remains fragmented across sectors.

While aging, climate change, and urbanization are widely recognized as global challenges, their intersection in China's policy system remains underexplored. This scoping review synthesizes national-level policy documents from 2020 to 2024, alongside selected sub-national practices, to map where age-friendly and sustainable policies align or diverge and to identify actionable opportunities for integration in urban development. We aim to provide policymakers, urban planners, researchers, and practitioners with an up-to-date picture of progress, gaps, and implementation levers to foster inclusive, resilient, and thriving urban environments for older adults.

## Methods

This scoping review follows the Joanna Briggs Institute (JBI) guidelines and adheres to the Preferred Reporting Items for Systematic Reviews and Meta-Analysis Extension for Scoping Reviews (PRISMA-ScR) 2020 standards.^19^ The review examines national-level policies in China concerning aging populations, age-friendly environments, sustainable development, and urban planning, organized using the PCC framework. The framework defines P (Population) as the general population, with a specific emphasis on aging individuals. C (Concept) encompasses key themes, including “age-friendly,” “healthy aging,” “sustainable environment,” “climate change,” and “livable communities.” The second C (Context) covers policies and interventions situated within the realms of urban planning, public health, and environmental sustainability in China.

### Information sources

The primary information sources for this scoping review are national government official websites, including the State Council and 25 affiliated departments (as provided in Supplementary Material: B-1. List of search databases). These grey literatures (news, case reports, etc.) identified via Microsoft Bing were only used to contextualize policy implementation examples and were not included in policy counts or formal synthesis.

### Search strategy and selection of sources of evidence

According to the aim and scope of our scoping review, we selected several Chinese keywords related to “age-friendly”, “environmentally sustainable”, and “cities and communities” in advance (see details below). Due to limitations in national government website search functions, which support only simple single-word queries without Boolean operators (e.g., 'AND,' 'OR,' 'NOT'), we applied a three-step approach for each website. First, we used several pre-specific Chinese keywords for ageing, sustainability, and urban development to conduct the initial search on each ministry's website. Secondly, all the search results from each website were merged, and duplicates were removed first within the same ministry and then across different ministries. Finally, an expanded search was conducted by reviewing references to other policies mentioned in the remaining records. The retrieval and screening process of the literature was repeated independently by two researchers (Jia Y. and Zhou W.), and any inconsistencies were discussed and agreed upon with a third researcher (Gao J.). The search window was Jan 1, 2020, to Dec 20, 2024. The pre-specific Chinese keywords used in the retrieval process are listed in the Supplementary Material.

### Eligibility criteria

Documents were included if they were (1) policy documents related to “age-friendly”, “healthy ageing”, “sustainable environment”, “climate change”, or “livable”, aiming to promote the health status for population especially aging populations; (2) issued by the ministries at the national level affiliated to the State Council; (3) published during Jan 1st, 2020 and December 20th, 2024. However, documents were excluded if the documents were aligned with one of these criteria such as (1) region-specific policy documents; (2) government follow-up responses or interpretations to previous policy regulations; (3) the policy purpose was the fact statement, for example, technical standard, specific clinical or pharmaceutical guidelines; (4) the full text of the policy was not publicly available. A calibration exercise preceded formal screening.

### Data extraction and charting

Data extraction of included documents was performed independently by two researchers (Jia Y. and Zhou W.), and any disagreements were resolved through a joint discussion. The form for data extraction was jointly discussed and formulated by all co-authors before the extraction process. Five documents were randomly selected to verify the rationality and comprehensiveness of the form design. We extracted the title of the policy documents, lead and supporting departments, date of publication, reference documents, main aims, interventions, and programs, and detailed aims, interventions, and programs for each eligible document. We also encode each policy document based on the policy type and policy objective attribute dimensions. Additionally, policies were encoded according to the key domains of age-friendliness and environmental sustainability mentioned in the content.

The retrieval records were managed and screened through EndNote 21 (Clarivate; reference de-duplication and screening lists), while the data extraction, coding, and charting were carried out using Microsoft Excel 2019 (Microsoft).

Following scoping-review conventions, we did not appraise the study quality of policy texts; instead, we emphasised breadth of coverage and mapping. PRISMA-ScR reporting standards were followed.

### Role of the funding source

The World Health Organization Western Pacific Regional Office had contributed to the study design, data interpretation, and manuscript revision. All other funding agencies had no involvement in study design, data collection, data analysis, data interpretation, or manuscript preparation.

## Results

### Selection of sources of evidence in scoping review

**Fig. 1:**
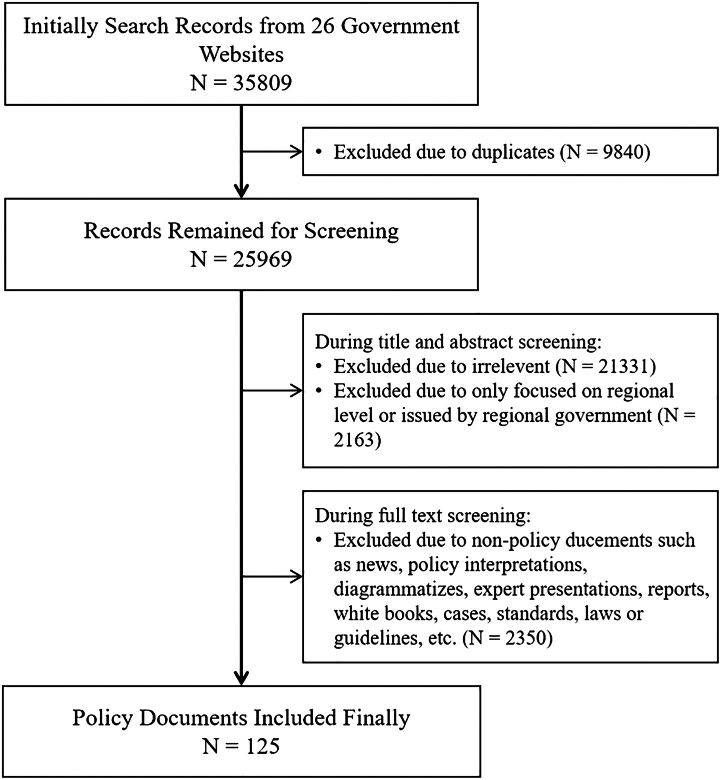
Flowchart for search and selection process.

**Fig. 2:**
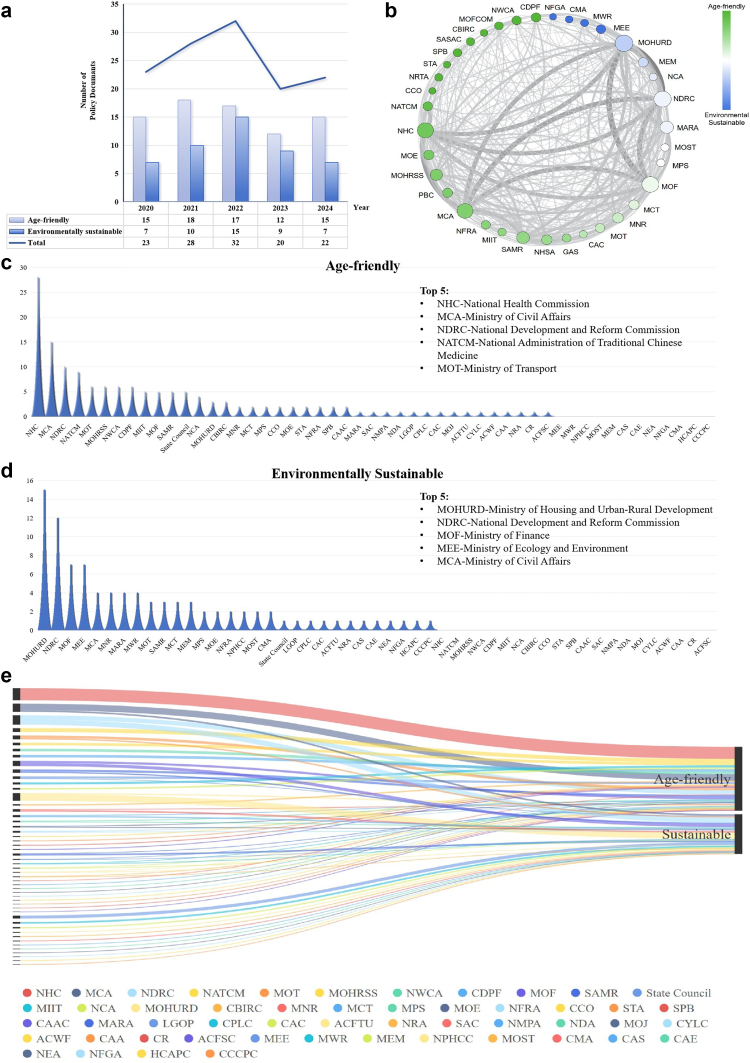
Basic characteristics of included policy documents and involved departments. a) refers to the number of policy documents grouped by year of issue. b) indicates the inter-departmental policy cooperation network. Each node represents a ceentral government department, an undirected edge links two departments when they co-appear in the same policy document. Edge thickness is proportional to the total number of co-issued documents between the pair; node size reflects the node's weighted degree in the overall network; node color refers to main policy orientation (green for age-friendly and blue for environmentally sustainable). c) the frequency of government departments involved in age-friendly policy documents. d) the frequency of government departments involved in environmentally sustainable policy documents. e) Sankey diagram of inter-ministerial contributions to age-friendly and sustainable policies. Left nodes are department (color coded, see legend), right nodes are age-friendly and sustainable agendas. Each colored stream denotes the number of national policy documents a department co-issued within the corresponding agenfa, stream width is proportional to that count. ACFSC, All-China Federation of Supply and Marketing Cooperatives; ACFTU, All-China Federation of Trade Unions; ACWF, All-China Women's Federation; CAA, China Aging Association; CAAC, Civil Aviation Administration of China; CAC, Cyberspace Administration of China; CAE, Chinese Academy of Engineering; CAS, Chinese Academy of Sciences; CBIRC, China Banking and Insurance Regulatory Commission; CCCPC, General Office of the Central Committee of the Communist Party of China; CCO, Central Civilization Office; CDPF, China Disabled Persons' Federation; CGCBS, Central Guidance Commission for Building Spiritual Civilization; CMA, China Meteorological Administration; CPCD, Central Propaganda Department; CPLC, Central Political and Legal Affairs Commission; CR, China State Railway Group Co., Ltd.; CSRC, China Securities Regulatory Commission; CYLC, Communist Youth League Central Committee; GAS, General Administration of Sport; HCAPC, Healthy China Action Promotion Committee; LGOP, State Council Leading Group Office of Poverty Alleviation and Development; MARA, Ministry of Agriculture and Rural Affairs; MCA, Ministry of Civil Affairs; MCT, Ministry of Culture and Tourism; MEE, Ministry of Ecology and Environment; MEM, Ministry of Emergency Management; MIIT, Ministry of Industry and Information Technology; MNR, Ministry of Natural Resources; MOE, Ministry of Education; MOF, Ministry of Finance; MOFCOM, Ministry of Commerce; MOHRSS, Ministry of Human Resources and Social Security; MOHURD, Ministry of Housing and Urban-Rural Development; MOJ, Ministry of Justice; MOST, Ministry of Science and Technology; MOT, Ministry of Transport; MPS, Ministry of Public Security; MVA, Ministry of Veterans Affairs; MWR, Ministry of Water Resources; NATCM, National Administration of Traditional Chinese Medicine; NCA, National Disease Control and Prevention Administration; NCHA, National Cultural Heritage Administration; NDA, National Data Administration; NDRC, National Development and Reform Commission; NEA, National Energy Administration; NFGA, National Forestry and Grassland Administration; NFRA, National Financial Regulatory Administration; NFRA, National Fire and Rescue Administration; NHC, National Health Commission; NHSA, National Healthcare Security Administration; NPHCC, National Patriotic Health Campaign Committee; NRA, National Railway Administration; NRTA, National Radio and Television Administration; NWCA, National Working Commission on Aging; PBC, People's Bank of China; SAC, Standardization Administration of China; SAMR, State Administration for Market Regulation; SASAC, State-owned Assets Supervision and Administration Commission; SPB, State Post Bureau; STA, State Taxation Administration.

The PRISMA flow diagram for search and selection of this scoping review is shown in Fig. 1. The initial search identified a total of 35809 documents. Among those, 9840 were removed due to duplication; then 21331 were excluded since irrelevant or out of scope during title and abstract screening, and 4513 during full-text screening. Finally, a total of 125 documents were included in the current scoping review. Detailed information on the included policy documents is listed in Table S1. Policies related to age-friendliness are largely distinct from those related to environmental sustainability in China, as indicated by the content of the included policy documents. Among the 125 included policy documents, age-friendly policies (N = 77) outnumber those focused on environmental sustainability (N = 48), and the issuance has a small peak in 2022 (Fig. 2a).

**Fig. 3:**
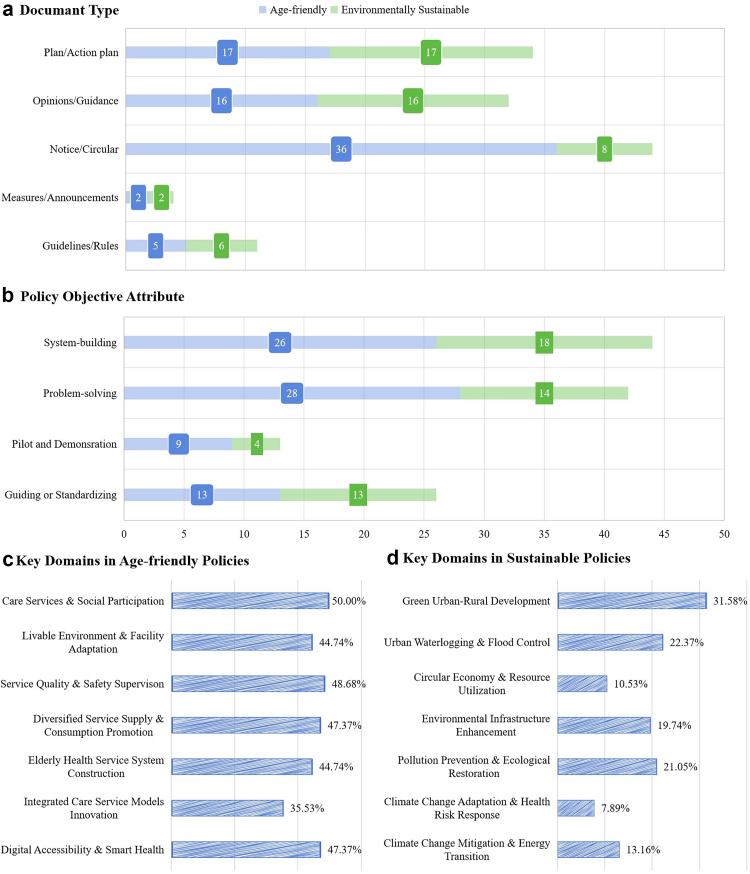
Typologies, objectives and key domains of included policy documents. a) Document Type. Horizontal bars show the number of documents by type. The numeric labels on bars are the exact counts for each agenda. b) Policy Objective Attribute. Bars show the number of documents whose primary objective was coded as System-building, Problem-solving, Pilot & Demonstration, or Guiding/Standardizing. Labels give counts by agenda. c) Key Domains in Age-friendly Policies. Bars show the share (%) of Age-friendly documents coded to each domain. Values are percentages of Age-friendly documents; Percentages are within-agenda shares and may not sum to 100% because multiple domains can be assigned to a single document. d) Key Domains in Sustainable Policies. Bars show the share (%) of Environmentally Sustainable documents coded to each domain. Percentages are within-agenda shares and may not sum to 100% because multiple domains can be assigned to a single document.

The distribution of document types differs by agenda (Fig. 3a): age-friendly is dominated by Notices/Circulars (N = 36) with a sizable share of Opinions/Guidance (N = 16), whereas the sustainable stream relies more on Plans/Action Plans (N = 17) and Opinions/Guidance (N = 16). Objectives also diverge (Fig. 3b): age-friendly policies skew toward problem-solving (N = 28) and system-building (N = 26), while sustainable policies emphasize system-building (N = 18) and guiding/standardizing (N = 13).

### Age-friendly and sustainable cities and communities in China's policy framework

#### City and administrative architecture

China's development of age-friendly and sustainable cities operates within a distinct policy framework shaped by unique administrative definitions. Cities function as hierarchical administrative divisions encompassing both urban and rural areas under their jurisdiction. This structure enables standardized policy implementation across 685 cities, categorized into five tiers by population size (from megacities to small and medium-sized cities),^20^ while simultaneously fostering metropolitan clusters, such as the Yangtze River Delta, through cross-jurisdictional coordination. At the grassroots level, the concept of “community” diverges fundamentally from Western notions of residential neighborhoods. Chinese communities, governed by residents' committees under sub-district administrations, serve as the smallest administrative units responsible for delivering care services to older people, organizing public health initiatives, and implementing Party-state directives, among other tasks.

#### Foundational policy and governance framework

“***Healthy China 2030” Planning Outline***^21^ and its subsequent implementation blueprint, the ***Healthy China Action (2019–2030)***,^22^ both issued during the 13th Five-Year Plan period (2016–2020), serve as the foundational framework guiding China's governance on health-related affairs in recent years. The establishment of a Healthy China has since become a central pillar of national development strategy, aiming to enhance public well-being and quality of life. These documents delineate not only measurable targets for improving population health outcomes (e.g., life expectancy extension, disease prevention, and healthcare service optimization), especially for older people, but also elevate the creation of a healthy environment to a core strategic objective. Within the healthy environment, the physical domain originally emphasized pollution mitigation (e.g., emission controls, indoor air quality standards, and risk assessment systems for water, soil, and atmospheric health) as well as disaster and emergency preparedness (e.g., urban public safety infrastructure development). However, growing climate impacts prompted a strategic expansion during the 14th Five-Year Plan period (2021–2025). The ***National Climate Change Adaptation Strategy 2035***^23^ and the ***National Climate Change Health Adaptation Action Plan (2024–2030)***^24^ formally integrate climate health risks and climate-resilient environments into national health governance, addressing critical gaps in earlier pollution-centric frameworks.

Despite shared targets in improving urban livability and public well-being, China's “age-friendly city” and “sustainable city” initiatives are governed by largely separate government departments in execution (Fig. 2c and d). The “Age-Friendly City” policies, primarily overseen by the Ministry of Civil Affairs (MCA) and the National Health Commission (NHC), focus on enhancing the social welfare and healthcare frameworks to accommodate the aging population (Fig. 2c). Meanwhile, the “Sustainable City” policies, mainly introduced by the Ministry of Ecology and Environment (MEE) and the Ministry of Housing and Urban-Rural Development (MOHURD), emphasize ecological balance and urban development (Fig. 2d). The National Development and Reform Commission (NDRC) is involved in policy documents in both domains, suggesting potential overlaps in policies and governance. As the cooperation network indicates (Fig. 2b), nodes cluster into two loosely connected groups (health - social protection vs environment - urban development) with relatively few cross-edges, which is consistent with the parallel policy streams observed in the texts. This administrative division, however, belies significant practical overlaps in built-environment interventions. Both agendas converge on enhancing urban green spaces, optimizing community micro-environments, upgrading disaster-resilient infrastructure, and promoting barrier-free green buildings. Such measures simultaneously address climate adaptation goals, such as mitigating heat island effects, and age-friendly priorities like improving accessibility for older residents. Yet the absence of institutional coordination mechanisms between these parallel systems not only risks diluting synergies but also leads to fragmented implementations: for example, duplicate infrastructure development may cause inefficiencies in resource allocation, the various climate and population backgrounds may lead to inconsistencies across local jurisdictions when implementation, separate management of green spaces and senior activity spaces may miss the opportunities for integrated policy-making. These gaps may potentially undermine the co-benefits of climate resilience and aging-in-place.

### Building age-friendly cities and communities

#### National level

According to the screening process, there are 77 policy documents related to age-friendly cities and communities (AFCC), systematically addressing the challenges of population aging through healthcare integration, social welfare expansion, and urban infrastructure modernization. Within age-friendly policies, leading domains are Care Services & Social Participation (50.0%) and Service Quality & Safety Supervision (48.68%), followed by Digital Accessibility & Smart Health and Diversified Service Supply (each 47.37%). Livable Environment & Facility Adaptation appears in 44.74% of documents (Fig. 3c).

The **14th *Five-Year Plan on Healthy Aging*** forms the cornerstone, prioritizing preventive healthcare, chronic disease management, and geriatric care integration. It aims to achieve a balanced distribution of health resources, enhance healthcare infrastructure, and integrate medical and care services for older people by 2025.^25^ Key initiatives include expanding health education, strengthening preventive healthcare systems, broadening palliative care services, incorporating Traditional Chinese Medicine (TCM) into palliative care systems, and developing a skilled healthcare workforce, with specific targets for training professionals in geriatrics, rehabilitation, and palliative care. Complementing this health-centric focus, the **14th *Five-Year Plan for National Aging Development and the Elderly Care Service System*** outlines structural reforms to expand care infrastructure for older people, advance preventive healthcare and geriatric medicine, and aim to universalize home-community-institutional care integration, develop a robust healthcare support system, including home-based medical services.^26^ This plan also emphasizes expanding pension and healthcare coverage and developing technological innovation to bridge urban-rural service disparities. Simultaneously, the **14th *Five-Year Plan for Actively Responding to Population Aging and Childcare Infrastructure Implementation*** introduces the “One Old, One Young” strategy, which pairs care for older people with childcare development through shared spatial and fiscal resources—for instance, repurposing underused training centers into inter-generational care hubs.^27^ The overarching framework is codified in the ***Opinions on Strengthening Aging Work in the New Era***, which elevates aging responsiveness to a national priority by mandating age-friendly housing retrofits, expanding pension coverage, and fostering the “silver economy” through entrepreneurship programs for older people.^28^ Together, these policies reflect a multi-dimensional approach that intertwines healthcare accessibility, social equity, and economic participation to empower aging populations within urban ecosystems.

#### Community level

The nationwide Elderly-Friendly Communities initiative, since 2020, has established a set of criteria for communities, comprising 40 benchmarks for urban areas and 30 standards for rural areas.^29^^,^^30^ These criteria include barrier-free housing retrofits, in–home medical services for disabled older individuals, meal assistance programs, tap water accessibility, regular visits to vulnerable older adults, mainly focused on the home-based age-friendly renovations and services (Supplementary Material C-1). Demonstration sites such as Xiamen's Yanwu Community integrate day care centers for older people, activity hubs with fitness equipment, and cultural programs.^31^ Besides, a series of community-led trainings and simplified health monitoring apps are applied to promote technological inclusivity, addressing challenges such as digital literacy gaps. Parallel measures—including transportation subsidies, free or discounted park and tourist admission, elevator retrofits in older walk-ups, community hospitals and clinicals, and public filial piety campaigns—reinforce accessibility and social inclusion, meeting the daily needs and spiritual well-being for older adults.

As a complement to these home-based age-specific efforts, the MOHURD promotes the comprehensive communities built with well-established 15-min life circles, an urban renewal model focused on spatial equity and multi-generational livability.^32^^,^^33^ It emphasizes universal service accessibility within a 15-min walk (around an 800 to 1000-m radius), co-locating healthcare, education, and commercial facilities. Care facilities for older people are prioritized among all in the planning of 15-min life circles, as older adults could experience a bigger barrier if they are facing declining mobility. Shanghai has issued the “***Planning Guidelines for 15-Minute Community Life Circle***” spearheadedly with a mature “1+N” model, to provide comprehensive services to community residents within a short distance (See supplementary material).^34^^,^^35^ In Handan and Beijing, other pilots integrate smart governance systems—such as real-time safety monitoring and telemedicine platforms—alongside ecological designs, including shaded walkways and rooftop gardens.^36^ For instance, Handan's Beigangyuan Community retrofitted a 1980s residential area with a smart management platform, combining care centers for older people, communal dining halls, and inter-generational activity spaces. Although not exclusively focused on older individuals, these communities often mandate accessibility features such as wheelchair-friendly parks and retrofitted housing, creating synergies with age-friendly objectives. This dual approach—offering specialized services for older people within universally accessible neighborhoods—ensures that aging populations are neither siloed nor overlooked in broader urban development.

### Co-benefits of sustainable cities and communities

#### Policy level

As the global climate warms, China has made many efforts in mitigating and adapting to climate change. Improving the ability and resilience of urban and rural construction and infrastructure to adapt to climate change is reiterated across major policy documents. Among the 125 included policy documents, 48 related to environmental sustainability and climate resilience. Sustainable policies cluster around Green Urban - Rural Development (31.58%) and Urban Waterlogging & Flood Control (22.37%), with Pollution Prevention & Ecological Restoration (21.05%) and Environmental Infrastructure (19.74%) close behind (Fig. 3d). Climate mitigation/energy transition (13.16%) and climate–health adaptation (7.89%) are less prominent, indicating a still-limited explicit health framing in this stream.

The ***Circular on Further Promoting the Nationwide Battle to Prevent and Control Pollution*** prioritizes reducing industrial and construction-related emissions, directly linking air quality improvements to urban livability goals.^37^ Besides, the ***Opinions on Promoting Green Development in Urban and Rural Construction*** and the **14th *Five-Year Plan for Building Energy Efficiency and Green Building Development*** jointly advance systemic decarbonization by institutionalizing energy-efficient building retrofits, renewable energy integration, and smart grid technologies.^38^^,^^39^ The ***National Climate Change Adaptation Strategy 2035*** expands this framework by mandating infrastructure upgrades such as flood-resilient drainage systems and green space networks to mitigate climate risks like heatwaves and extreme rainfall,^23^ while the ***National Climate Change Health Adaptation Action Plan (2024–2030)*** integrates public health safeguards into urban and climate governance through various pathways such as early warning systems for extreme weather and climate events, climate-sensitive disease surveillance, and healthcare emergency preparedness and dynamic assessments of climate change related health risks vulnerabilities and adaptation capacities.^24^ Together, these policies mark a shift from isolated pollution control to holistic climate action, weaving environmental remediation, carbon reduction, and health co-benefits to enhance air quality, water safety, and thermal comfort, and thereby urban livability.

Against the backdrop of rapid urbanization and escalating climate risks, China's pursuit of **carbon peaking before 2030** and **carbon neutrality before 2060** necessitates a systemic green overhaul of cities, the primary contributors to national emissions.^40^ Urban transformation strategies integrate industrial restructuring, energy mix optimization, and low-carbon transportation systems with large-scale ecological restoration and the development of green infrastructure. Key initiatives include energy-efficient building retrofits (enhance indoor comfort), stormwater management systems (reduce urban inland inundation and subsequent infectious diseases risk), and the expansion of urban green spaces through forest cities, pocket parks, and garden city models (support walkable areas and opportunities to exercise and leisure). The “Greenery within 300 m, Parks within 500 m” vision exemplifies this approach, enhancing climate resilience by embedding accessible green networks into the urban fabric.^23^ Parallel efforts such as railway and highway greening, coupled with carbon market mechanisms, synergize emission reduction with ecological benefits.

#### Implementation level

**Fig. 4:**
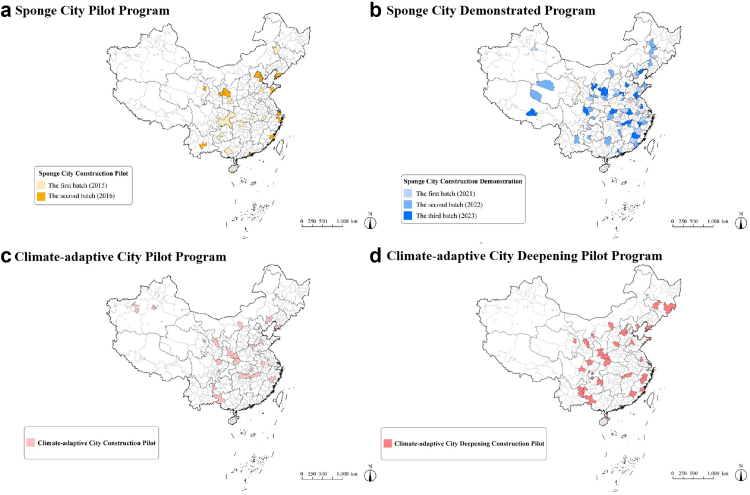
Geographical distribution of the a) sponge city pilot program, b) sponge city demonstrated program, c) climate-adaptative city pilot program, and d) climate-adaptative city deepening pilot program.

At the implementation level, China encourages all cities to adopt holistic, sustainable, and climate resilience strategies while prioritizing pilot programs in selected regions to exemplify and scale innovative solutions (see detailed information in Supplementary Material). The Sponge City Pilot and Demonstrated Program,^41^, ^42^, ^43^ initiated in 2015 across 30 pilot cities (Fig. 4a and b), employs green infrastructure such as rain gardens and smart water management systems to enhance stormwater retention, as demonstrated in Shanghai's Suzhou River Walkway project.^44^ To further address the climate pressure, the Climate-Adaptive Pilot Cities initiative, launched in 2017 and expanded to 39 cities by 2023, tailors resilience strategies to regional risks (Fig. 4c and d).^45^^,^^46^ Shanghai has strengthened coastal flood defenses in response to sea-level rise and an increase in storm surges,^44^ while Guangzhou deployed permeable pavements and urban wetlands to mitigate heat island effects,^47^ with documented temperature reductions in targeted areas. These efforts align with broader greening campaigns, including Shenzhen's Forest City initiative, which has significantly increased urban tree coverage and carbon sequestration capacity,^47^ and Chengdu's Park City model, which prioritizes accessible green corridors for residents.^48^ Smaller-scale interventions, such as Beijing's Pocket Parks, repurposing underutilized spaces, offer scalable neighborhood-level solutions. However, these pilots and initiatives have largely been implemented in eastern coastal cities; uptake in inland or less-resourced areas has been more limited, potentially exacerbating inequities in environmental protection and age-friendly infrastructures.

Large-scale ecological restoration further reinforces urban sustainability objectives. The Three-North Shelter Forest Program, spanning over 4 million square kilometers, has substantially increased forest coverage in northern regions since 1978, contributing to reduced desertification and improved agricultural productivity.^49^ However, challenges persist in arid zones, where water scarcity limits tree survival rates, prompting shifts toward drought-resistant native species in areas like Inner Mongolia.

## Discussion

### Overview of included scoping reviews

#### Policy landscape and cross-cutting synergies

This scoping review synthesizes 125 national-level policy documents to map China's efforts toward age-friendly and sustainable cities and communities since 2020. To support the key information and initiatives mentioned in these policy documents, we also listed several ongoing programs and exemplified cases, aiming to present a more comprehensive and integrated perspective of current China (Table 1; Supplementary Materials: C, D). These policies reveal two largely distinct, but sometimes overlapping, policy streams: (1) an age-friendly stream focused on care for older adults, healthcare integration, and social equity, and (2) a sustainable stream prioritizing climate resilience, low-carbon transitions, and ecological restoration. The overarching framework is anchored by ***Healthy China 2030*** and ***National Climate Change Adaptation Strategy 2035***, while sector-specific plans like the **14th *Five-Year Plan on Healthy Ageing*** and ***Opinions on Promoting Green Development in Urban and Rural Construction***, translate mandates into measures such as care infrastructure upgrades, green building retrofits, and renewable energy integration.

**Table 1:** Overview of key national policies, programs, and practice cases in China.

Domain	National Level	Ministries	Cases in Sub-National Level^a^
Main guideline	Healthy China 2030	CCCPC, State Council	
	Healthy China Action (2019–2030)	NHC	
Age-friendly	“14th Five-Year Plan” on Healthy Ageing	NHC; MOE; MOST; MIIT; MOF; MOHRSS; MOHURD; MVA; SAMR; NRTA; GAS; NHSA; CBIRC; NATCM; CDPF	• Panan County's “meteorology + TCM + wellness tourism” model for elderly care, integrating environmental and health resources. • Yanwu Community in Xiamen: Elderly-friendly community integrating commerce, tourism, and education, with care services like day care and fitness facilities. • 15-min life circle in Meishan and Shanghai: Concept to ensure essential services within a 15-min walk/bike ride, improving community well-being.
	The “14th Five-Year Plan” for Actively Responding to Population Aging and Childcare Infrastructure Implementation Plan	NDRC; MCA; NHC	
	The “Opinions of the Central Committee of the CPC and the State Council on Strengthening Aging Work in the New Era”	CCCPC, State Council	
	Notice on the Establishment of National Demonstration Age-Friendly Communities	NHC; NWCA	
	National Standards for Demonstration Age-Friendly Urban and Rural Communities (Trial)	NHC	
	Three-year Action Plan to Comprehensively promote the Construction of Urban one-quarter Hour Convenient Living Circle (2023–2025)	MOFCOM; NDRC; MCA; MOF; MOHRSS; MNR; MOHURD; MCT; NHC; SAMR; NFRA; GAS; SPB	
Environmentally Sustainable	National Climate Change Adaptation Strategy 2035	MEE; NDRC; MOST; MOF; MNR; MOHURD; MOT; MWR; MARA; MCT; NHC; MEM; PBC; CAS; CMA; NEA; NFGA	• Dual-purpose infrastructure development in Beijing to enhance urban resilience to climate change • Dual-purpose infrastructure development in Beijing • Shanghai's sponge city initiative, using permeable pavements, green roofs, and rain gardens to manage water, reduce floods, and enhance sustainability.
	14th Five-Year Plan for National Urban Infrastructure Construction	MOHURD; NDRC	
	The Opinions on Promoting Green Development in Urban and Rural Construction	CCCPC; General Office of the State Council	
	The Five-Year Action Plan for the In-depth Implementation of the People-Centered New Urbanization Strategy	the State Council	
	14th Five-Year Plan for Building Energy Efficiency and Green Building Development	MOHURD	
	Guiding Opinions on Actively and Steadily Promoting the Construction of Public Infrastructure for Both Peacetime and Emergency Use in Megacities	the State Council	
	Notice on the Pilot Work of Building climate-resilient Cities	MOHURD; NDRC	
	Notice on Further Clarifying the Relevant Requirements of Sponge City Construction Work	MOHURD	

CBIRC, China Banking and Insurance Regulatory Commission; CCCPC, Central Committee of the Communist Party of China; CDPF, China Disabled Persons' Federation; CMA, China Meteorological Administration; CAS, Chinese Academy of Sciences; GAS, General Administration of Sport; MARA, Ministry of Agriculture and Rural Affairs; MCA, Ministry of Civil Affairs; MCT, Ministry of Culture and Tourism; MEE, Ministry of Ecology and Environment; MOE, Ministry of Education; MOF, Ministry of Finance; MOFCOM, Ministry of Commerce; MOHRSS, Ministry of Human Resources and Social Security; MOHURD, Ministry of Housing and Urban-Rural Development; MIIT, Ministry of Industry and Information Technology; MEM, Ministry of Emergency Management; MNR, Ministry of Natural Resources; MOST, Ministry of Science and Technology; MOT, Ministry of Transport; MVA, Ministry of Veterans Affairs; MWR, Ministry of Water Resources; NATCM, National Administration of Traditional Chinese Medicine; NDRC, National Development and Reform Commission; NEA, National Energy Administration; NFGA, National Forestry and Grassland Administration; NFRA, National Food and Reserves Administration; NHSA, National Healthcare Security Administration; NHC, National Health Commission; NRTA, National Radio and Television Administration; NWCA, National Working Commission on Aging; PBC, People's Bank of China; SAMR, State Administration for Market Regulation; SPB, State Post Bureau.

a See detailed information of “Cases in Sub-National Level” in Supplementary Material.

Key synergies emerge in areas such as green infrastructure (e.g., pocket parks serving dual purposes for recreation and stormwater management) and community accessibility (e.g., barrier-free designs aligning with climate-resilient urban renewal), as they can share the spatial and functional targets. Spatial planning models that embed care, mobility, and environmental features within a 15-min life circle could offer templates for bridging age-friendly and climate-resilient design, to improve everyday convenience for the elderly when reducing car dependence and the associated fuel consumption and emissions.^50^ The shaded green corridors, designed to mitigate urban heat islands, will also enhance walkability for older adults. Yet these initiatives that could bring win–win features in both age-friendliness and environmental sustainability are not widely reflected in national policy mandates. Institutional fragmentation also creates barriers. Age-friendly policies, led by the MCA and NHC, and sustainable urbanization programs, overseen by the MEE and MOHURD, exhibit minimal coordination. Embedding such shared outcomes into policy frameworks can optimize resource allocation and stakeholder engagement. Such coordination is not only beneficial for older people but also for other age groups.

#### Age-friendly governance, instruments, and international comparisons

China's age-friendly policies and initiatives combine healthcare integration and social welfare expansion through integrated medical-care models, home-based care, smart health monitoring, and community support. Targeted interventions, such as centralized care facilities, home visit programs, and long-term insurance schemes, are designed to support individuals facing structural disadvantages, including those with limited financial resources, disabilities, or complex care needs, many of whom are older adults. Simultaneously, initiatives fostering lifelong learning, flexible employment, and cultural engagement cater to the aspirations of the “dynamic older people”, promoting active social participation. A pivotal governance shift in 2023 further solidified this holistic vision, transferring the governance of affairs related to older people from the NHC to MCA, thereby reframing aging policy within a broader social welfare framework. Confronted with the multifaceted challenges of building age-friendly cities and addressing complex aging-related societal needs, this institutional realignment has facilitated structural coordination across departments, thereby alleviating fragmented policy implementation. While challenges persist in standardizing metrics and resource allocation, the emphasis on cultural narratives of “active aging” and inter-generational equity signals a transformative phase, positioning older citizens not merely as care recipients but as vital contributors to sustainable urban development.

China's age-friendly and healthy city policies predominantly rely on supply-based interventions, such as infrastructure development and healthcare expansion, which account for 62.9% of policy content. However, demand-oriented policy tools, including social participation and market cultivation, are significantly underutilized, comprising only 10.6% of policies, with social participation at 69.2% and market cultivation at 30.8% within this category. To enhance the effectiveness of age-friendly city initiatives, policymakers should incorporate demand-based tools like government procurement of age-friendly services, outsourcing to private providers, and consumption incentives to encourage community engagement. Such measures could mobilize older adults and private sectors, fostering sustainable and inclusive urban environments.^51^ In an international perspective, China's strengths in care system consolidation and barrier-free retrofits align with trends seen in Japan (universal design linked to everyday mobility and disaster preparedness)^52^ and Australia (green-blue infrastructure integrated with walkable, service-rich neighbourhoods).^18^ Looking forward, routinely embedding transport and housing metrics and age-disaggregated resilience indicators in adaptation plans and scaling co-funded dual-benefit projects (e.g., sponge-city upgrades paired with accessibility retrofits) would enhance transferability and impact.

### Comparing and contrasting the WHO's age-friendly framework and China's approach

#### Domains emphasised and under-emphasised in China

China's age-friendly policies focused the health and medical services, living and outdoor environments, social participation, social security, the silver economy, and barrier-free facilities. Compared with the eight core themes determined by WHO (Table 2),^53^ China pays more attention to the construction level of the care service system and various security systems, emphasizing the management and security of service work for older people. However, aspects such as “housing” and “transportation” receive less emphasis in China, with a greater focus on reforming barrier-free public infrastructure. This configuration risks a “break in the chain” when older residents face mobility and affordability constraints in ageing neighbourhoods. Furthermore, the development of age-friendly cities in China, particularly in areas such as “community support & health services” and “respect & social participation,” incorporates unique elements such as the integration of traditional Chinese medicine and healthcare services, as well as the promotion of community cultural activities like Tai Chi, Mahjong, and Square Dancing.

**Table 2:** Horizontal comparison of healthy aging related policies in China and the WHO AFCC framework.

Domain	WHO AFCC Framework	Healthy China 2030 Plan Outline	14th Five-Year Plan for Healthy Aging
Health Services			
Community and Health Care	Provide appropriate eldercare services, such as preventive care, geriatric clinics, hospitals, adult day centers, respite care, rehabilitation care, nursing home care, home care, and palliative care, conveniently located near where older people live, and delivered by trained healthcare and social workers.	Promote healthy aging, advance the construction of the elder medical and health service system, and extend healthcare services to communities and families. Strengthen community medical services and health management, especially chronic disease management and health support for older adults.	Support home (community) care services, encourage social forces to establish nursing stations, providing home health services for disabled older adults; advance the development of the integrated medical-nursing service system, integrating home, community, and institutional care to improve care for disabled older adults.
Traditional Chinese Medicine in Elder Health	Not specifically mentioned.	Promote the integration of TCM with elderly care services, supporting medical-nursing integration, providing treatment during hospitalization, rehabilitation care, long-term living care, and palliative care for older adults.	Develop TCM services for elderly health, promote TCM's role in prevention, rehabilitation, and palliative care; actively conduct TCM health check-ups, health assessments, health interventions, and educate on herbal food therapy; promote traditional Chinese exercise programs and cultivate healthy living habits and lifestyle concepts.
Mental Health Services	Not specifically mentioned.	Promote elderly mental health and care services, strengthening effective interventions for conditions like dementia.	Implement elderly mental care actions. Summarize and promote successful elderly mental care experiences, continuously expand coverage; provide counseling, emotional support, and grief relief services, especially for older adults with special difficulties.
Chronic Disease Management & Health Management	Not specifically mentioned.	Promote comprehensive chronic disease prevention and management services closely integrated with home, community, and institutional elderly care.	Build a comprehensive chronic disease prevention and treatment system. Strengthen early screening, intervention, classification management, and health guidance for key chronic diseases such as hypertension, diabetes, coronary heart disease, and neurodegenerative diseases like Alzheimer's and Parkinson's.
Health Education and Knowledge Promotion	Learning about aging and older people is included in primary and secondary school curricula; active participation of older people in school interaction activities.	Improve national health literacy, advance the national healthy lifestyle campaign; increase media coverage of health science knowledge, actively create and regulate various health programs in radio and television, and use new media to expand health education.	Elderly Health Education Special Project: Implement programs to promote elderly health literacy. Monitor elderly health literacy and TCM health literacy, conduct targeted health education activities, continuously increase awareness of key health information, and improve the health literacy of older adults; hold Elderly Health Promotion Weeks with annual themes to raise awareness of elderly health in urban and rural areas.
Infrastructure Construction			
Housing	Provide affordable, well-designed, safe, and elderly-friendly housing with age-friendly, barrier-free design and modification services to ensure safety.	Not specifically mentioned.	Accelerate the construction of barrier-free environments and the modification of housing to be age-friendly. Promote the installation of AEDs in elderly care facilities.
Outdoor Spaces and Buildings	A clean and pleasant city with green spaces, well-maintained recreational areas, adequate rest areas, and well-built, safe pedestrian walkways, crosswalks, and building infrastructure, as well as a safe environment.	Healthy city and village construction, ensuring the allocation of public facilities related to health, improving the system, layout, and standards of public facilities, and integrating health into urban and rural planning, construction, and governance.	Strengthen the construction of fitness facilities in urban and rural communities and integrated medical-nursing institutions to enhance the degree of age-friendliness.
Transportation	Provide affordable, convenient, and safe public transportation; driving conditions and parking facilities suitable for older adults.	Strengthen road traffic safety facility design, planning, and construction, implement highway safety protection projects, and address traffic safety hazards.	Not specifically mentioned.
Social Atmosphere & Participation			
Social Participation	Provide a series of accessible and affordable activities to encourage older adults to engage in social, volunteer, educational, and cultural activities, strengthening social connections; promote inter-generational interaction, ensuring older adults connect with other age groups and cultures in the community.	Not specifically mentioned.	Promote the integration of physical health and sports. Research and promote suitable sports and fitness activities for older adults; release a guide for older adults' fitness activities and organize relevant events.
Civic Participation and Employment	Provide volunteer activities and employment opportunities for older adults, supporting their participation in the labor force and ensuring continued self-sufficiency; create a positive attitude among employers to retain and hire older workers, and support older adults' involvement in social decision-making and political processes.	Not specifically mentioned.	Not specifically mentioned.
Respect and Social Inclusion	Strengthen aging education, increase awareness of aging issues, and promote respect for older adults; the media should actively portray older adults in a positive and healthy light; encourage their participation in community activities and community building; ensure economically disadvantaged elderly have equal access to public services and cultural activities.	Not specifically mentioned.	Cultivate professionals in elderly health care; establish a framework for long-term care insurance. Encourage commercial insurers to develop exclusive products for older adults, such as disease insurance, long-term care insurance, and accident insurance.
Communication and Information	Ensure that information is easily accessible and help older adults maintain connections with society through appropriate media.	Build a health information service system; establish a unified, authoritative, and interconnected health information platform for population health, promote "Internet + healthcare" services, and innovate healthcare service models using the internet.	Use digital platforms to disseminate health knowledge and improve the ability of older adults to access information and health literacy.
Technology and Industry Development	Not specifically mentioned.	Promote health technology innovation, develop the health industry, and integrate health with elderly care, tourism, internet, fitness, and food industries to create new health-related industries, business models, and trends.	Promote the deep integration of elderly health care with industries such as elderly care, health preservation, culture, tourism, sports, and education; support the use of emerging technologies like artificial intelligence, virtual reality, and new materials in elderly health; enhance the age-friendliness of smart products and wearable devices; leverage information technologies like IoT, big data, and the internet to innovate service models and improve elderly health service quality and efficiency.

AED, automated external defibrillators; AFCC, age-friendly cities and communities; IoT, Internet of things; TCM, traditional Chinese medicine; WHO, World Health Organization.

#### Cultural and participation features

In China, age-friendly communities place significant emphasis on Party building, leadership, and political requirements. Under the management of community grid workers, retired older individuals often volunteer, engaging in community work and activities, thereby contributing to community development. Besides, the existing community resources can also be well leveraged when promoting the age-friendliness of communities. For example, grid workers are assigned to specific buildings or neighborhoods according to community conditions, allowing them to gain a comprehensive understanding of the basic status of older residents. The phenomenon of inter-generational caregiving is also prominent in China, as the traditional Chinese concept of 'filial piety' emphasizes children's primary responsibility for elder care.^27^^,^^54^^,^^55^ Age-friendly initiatives are often linked to child-friendly policies and are commonly addressed together in policy frameworks under the concept of “one old, one young.” New explorations of creating the model and space for the coexistence of older adults and children are rising to balance the population aging and fertility decline. “Time bank” encourages young volunteers to accumulate future elderly-care services by providing such services to older adults. Cultural and social dynamics significantly shape the effectiveness of age-friendly city policies in China. Traditional values like filial piety, which emphasize family-based caregiving, are challenged by rapid urbanization, reducing immediate family support for older adults. Policies should integrate culturally appropriate solutions, such as inter-generational community spaces that foster social inclusion while respecting traditional values. Additionally, addressing barriers like low digital literacy and privacy concerns in smart city initiatives can enhance social connectedness for older adults, ensuring policies are both culturally sensitive and inclusive.^56^

### Populations disproportionately affected by climate change and promoting community engagement in climate resilience

#### Priority groups and vulnerable locations

China's ***National Climate Change Adaptation Strategy 2035***^23^ addresses the prioritization of vulnerable populations, including older people(65 years and older), children, pregnant women, individuals with chronic diseases, poverty-stricken populations, as well as residents of urban areas with inadequate infrastructure, and those living in regions prone to extreme weather events such as high temperatures, floods, and typhoons. Resilience is defined as the capacity to withstand, adapt to, and recover from the adverse impacts of climate change. It involves enhancing the robustness of both natural ecosystems and socioeconomic systems against climate risks. To operationalise these priorities, the strategy calls for targeted assessments, risk evaluations, and tailored adaptation measures that strengthen resilience in health systems, upgrade infrastructure, and enhance emergency preparedness.

However, whether these priorities are sufficient depends on the actual implementation, the extent to which these measures reach the most vulnerable, and the effectiveness of community engagement efforts. To address these challenges, the strategy specifies near-term actions by 2025, including research to identify health risks, vulnerable areas, and populations related to extreme weather, development of adaptation strategies and technologies, and the launch of pilot actions in cities, rural areas, and key sites (schools, hospitals, and nursing homes), with nationwide implementation by 2035. Critical infrastructure enhancements include strengthening general hospitals, disease control centers, centers for older people, kindergartens, municipal pipeline networks, transportation systems, parking lots, charging stations, sewage and waste treatment facilities, and industrial platform support facilities. These measures aim to foster inclusive, community-driven initiatives that enhance adaptive capacity and mitigate the impacts of climate change across China.^23^

#### Engagement levers and indicator gaps

Community engagement measures include public education on climate risks, promoting grassroots involvement in adaptation actions, enhancing local disaster response capabilities, and the broad mobilisation of enterprises, communities, associations, and citizens. China promotes the diversification of participants in adaptation initiatives by organizing networked coordination mechanisms between communities and enterprises, expansion of volunteer teams, and a societal atmosphere of extensive participation. This approach aims to harness the collective strength of the entire society to effectively enhance climate resilience. Effective delivery, however, depends on robust local governance and consistent national support.

A notable gap remains; there are no specific measures or indicators in place to address extreme events and climate disasters affecting older people in age-friendly cities in China. From the perspective of urban safety and disaster prevention, China has been promoting the construction of “safe cities” and “resilient cities” under the supervision of national emergency management departments. The relevant actions and measures are intended for the entire population, with no special emphasis on older individuals. Additionally, disaster victim statistics have not typically emphasized age group data, even though many older adults live in ageing residential communities, which may be at increased risk during climate-related events. These older buildings often have structural vulnerabilities that heighten exposure to climate hazards.

### Challenges and recommendations for the future

#### Fragmented governance and misaligned metrics

In this era of severe climate change and accelerating population aging, China still faces numerous challenges and difficulties due to fragmented governance and misaligned institutional priorities. While national policies are increasingly acknowledging the interconnection between aging and climate resilience, implementation gaps persist due to siloed administrative mandates, competing performance metrics, and limited cross-sectoral coordination (Fig. 2e). The Sankey view visualises this split: cross-stream flow is thin, with only a small set of agencies (notably NDRC) contributing substantively to both agendas. In practice, climate adaptation strategies often overlook age-specific vulnerabilities, while age-friendly urban renewal projects rarely prioritize ecological co-benefits, such as stormwater retention or carbon sequestration. Local governments, constrained by rigid funding structures and sector-specific targets, struggle to reconcile care objectives with sustainability goals, resulting in duplicated efforts and missed opportunities for synergistic outcomes.

In short, integration is weakest at the interface between climate adaptation plans and age-friendly urban renewal. The former rarely include age-disaggregated heat/flood indicators, while the latter seldom carry ecological co-benefit KPIs (e.g., stormwater retention, carbon sequestration). Ring-fenced budgets and single-agency performance targets compound this split, discouraging joint planning and pooled delivery.

#### Regional disparities and local adaptability

A strong national framework does not automatically translate into local effectiveness. Ascertain the number of older individuals in rural versus urban areas and their differing needs for social participation and infrastructure support. Research shows that policy strength varies significantly across provinces, with rural older adults (mean social participation score of 0.29) benefiting more from physical infrastructure improvements compared to urban older adults (mean score of 0.81).^57^ These differences imply scenario-specific priorities: island and rural jurisdictions need access-first investments such as cooling shelters, barrier-free transit nodes, and digital access, whereas large cities should emphasize participation-oriented services and retrofits near daily destinations. Systemic equity and efficiency tensions also remain. Urban-rural disparities in green infrastructure investment are evident, with coastal cities historically receiving disproportionate funding for initiatives like the Sponge City Program than inland cities (Fig. 4). Current transfers and earmarks seldom reward dual-benefit results, which helps explain the coastal-inland skew in green-infrastructure funding. Fragmented governance across environmental, urban planning, and transport sectors has complicated implementation, though recent institutional reforms aim to centralize ecological oversight. Emerging models, such as Xiong'an New Area's renewable energy transition and pilot smart mobility systems, reflect evolving priorities to integrate technological innovation with citizen-centric green development.

#### Governance and delivery mechanisms

Addressing these challenges requires a paradigm shift toward inclusive, adaptive governance models that bridge policy domains while respecting regional diversity. At the national level, fostering inter-ministerial collaboration could involve establishing flexible frameworks to align aging and sustainability agendas, such as integrating older people's health risk assessments into climate adaptation planning or encouraging dual-benefit infrastructure investments through pooled funding mechanisms. Local governments might adopt participatory planning processes that engage communities in co-designing climate-resilient public spaces. For example, shaded walkways in neighborhoods with high older populations could be prioritized, or community centers could be retrofitted to serve both as cooling shelters and social hubs. Such approaches would benefit from decentralized decision-making, allowing municipalities to tailor solutions to local demographic and ecological conditions rather than adhering to uniform mandates.

#### Grassroots innovation and the role of older adults

Simultaneously, empowering grassroots innovation could amplify older people's agency in sustainability transitions. Communities might explore inter-generational partnerships that leverage older people's traditional ecological knowledge alongside youth-driven technological solutions, such as collaborative urban gardening projects that enhance biodiversity while fostering social cohesion. Public awareness campaigns could highlight the mutual benefits of integrating older people's needs into green infrastructure development, cultivating broader societal recognition of aging populations as vital contributors to climate resilience rather than passive care recipients. Older individuals should be formally recognized and encouraged as active contributors to climate resilience. Policy frameworks could motivate their participation as community stewards in healthy ageing affairs or disaster preparedness networks, leveraging their social capital and traditional ecological knowledge.

#### Quantifying co-benefits & integrated indicator

Based on these efforts, research and capacity-building initiatives should prioritize evidence-based strategies to quantify and scale co-benefits. Academic institutions could partner with policymakers to evaluate how age-friendly retrofits, such as energy-efficient age-friendly housing or accessible green spaces, simultaneously reduce carbon footprints and improve health outcomes. Training programs for local officials might emphasize adaptive governance skills, equipping them to balance competing priorities in resource allocation and risk management. China's healthy and age-friendly city initiatives could be strengthened by better integrating the five key domains: health services (21.5% of policy focus), a healthy environment (31.3%), a healthy society (13.8%), a healthy culture (19.5%), and a healthy lifestyle (13.8%). The current policy landscape often prioritizes health services and environmental measures, potentially overlooking opportunities for synergy across domains. For instance, integrating green infrastructure, such as pocket parks, with community spaces designed for older adults could simultaneously address climate resilience and social inclusion. Adopting the “integrating health into all policies” approach could foster more cohesive urban development strategies.^58^ By embracing collaborative approaches, such as establishing an inter-ministerial coordination mechanism, adopting integrated planning mandates, or developing co-funding schemes for dual-purpose infrastructure, we can transform its aging and sustainability agendas from parallel tracks into a cohesive strategy.

#### Policy implications

Taken together, aligning ageing and sustainability requires a common evaluative language and coordinated delivery. Embedding age-responsive indicators into climate-adaptation plans and adding climate/decarbonisation metrics to age-friendly programmes would create that shared framework. At the financing and project level, co-funding arrangements should prioritise dual-benefit infrastructure (e.g. shaded corridors and green–blue networks implemented alongside barrier-free retrofits) so that each investment advances both resilience and inclusion. Addressing regional disparities calls for scenario-specific priorities (rural areas emphasising infrastructure and access; urban areas emphasising participation-oriented services).^58^ Governments should also encourage public procurement of age-friendly services, social outsourcing, and mobilise communities and private providers. Finally, establishing a shared monitoring registry that links health and climate outcomes (heat exposure, flood resilience, accessibility, greenery) would allow routine co-benefit assessment and timely course correction, gradually shifting ageing and sustainability from parallel tracks to a coherent strategy.

### Conclusion

Drawing on 125 national-level policies since 2020, this scoping review shows that the age-friendly and sustainable policies are two largely parallel streams with limited coordination. Co-benefit levers including green/blue infrastructure, barrier-free retrofits, 15-min life circles are present but not yet mainstreamed. Governance fragmentation and misaligned metrics impede delivery, and regional disparities persist.

Future research work should also move beyond description to comparative and empirical evaluation. Cross-country studies could test which governance models, indicators, and financing arrangements best integrate ageing and climate agendas. Quasi-experimental assessments should estimate implementation impacts on health, heat/flood resilience, emissions, and cost-effectiveness. In-depth qualitative research with older residents, implementers, and planners can surface barriers to co-production and contextual design. Priorities also include developing standardized, age-disaggregated indicators and shared monitoring registries, evaluating co-funding schemes for dual-benefit infrastructure, and testing demand-side mechanisms that mobilize participation. Taken together, these directions would convert policy intent into measurable co-benefits and strengthen transferability across diverse urban and rural settings.

## Contributors

John S. Ji, Siwon Lee, and Yanhui Jia conceived the study. Yanhui Jia, Jiuxuan Gao, Weiju Zhou and John S. Ji designed the methods and analysis framework. Yanhui Jia, Jiuxuan Gao, and Weiju Zhou searched, screened, and reviewed the policy documents. Yanhui Jia and Weiju Zhou extracted data from the literature. Yanhui Jia conducted statistical analyses. Yanhui Jia and John S. Ji led the writing processes. Weiju Zhou, Siwon Lee, Mikiko Kanda, Pankyu Park, and Sally J. Edwards contributed to finding interpretation and draft revision. The authors approved the final version of the manuscript. John S. Ji had final responsibility for the decision to submit for publication.

## Data sharing statement

The data used in this study are extracted from open-accessed policy documents. All data analyzed or produced as a result of this review are included in the main file and Supplementary Materials. Full text of policy documents can be searched on the corresponding websites.

## Editor note

The Lancet Group takes a neutral position with respect to territorial claims in published maps and institutional affiliations.

## Declaration of interests

We declare no competing interests.
