# Supplementary material for: Sustainable age-friendly cities and communities in China: a scoping review and narrative assessment of national policies
Source: Lancet Reg Health West Pac. 2025 Nov 7;64:101723. doi: 10.1016/j.lanwpc.2025.101723 (PMC12639885; doi:10.1016/j.lanwpc.2025.101723)
Supplement: Supplementary Materials [file mmc1.pdf]

Supplementary Materials

Sustainable Age-Friendly Cities and Communities in China: A Scoping Review and Narrative Assessment of National Policies

Authors: Yanhui Jia[1], Siwon Lee[2], Mikiko Kanda[2], Pankyu Park[2], Sally J. Edwards[2], Jiuxuan Gao[1], Weiju Zhou[1], John S. Ji[1,*]

Affiliations:
[1] Vanke School of Public Health, Tsinghua University, Beijing, 100084, China
[2] World Health Organization (WHO) Regional Office for the Western Pacific, Manila, Philippines

***Correspondence to:**
John S. Ji, ScD
Associate Professor
Vanke School of Public Health
Tsinghua University, Beijing, 100084, China
email: johnji@tsinghua.edu.cn

Author Contact Information:
Yanhui Jia, PhD candidate: jiayh23@mails.tsinghua.edu.cn;
Siwon Lee: alee@who.int;
Mikiko Kanda: mkanda@who.int;
Pankyu Park: parkp@who.int;
Sally J. Edwards: edwardss@who.int;
Jiuxuan Gao, MPH: qddxgaojiuxuan@163.com;
Weiju Zhou, PhD: weijuzhou@tsinghua.edu.cn

Table of Contents

A. Glossary for key words mentioned in included policy documents. **3**

B. Search Strategy. **17**

B-1. List of search databases. 17

B-2. List of pre-specific Chinese keywords used in searching process. 18

C. Innovative programs and initiatives in China **19**

C-1. Age-friendly demonstrated communities in China 19

C-2. Comprehensive Communities and Urban Renewal 21

C-3. Pilot Project for Climate-Adaptive Cities 23

C-4. Sponge City Pilot and Demonstration Cities in China 25

C-5. Forest City, Park City, and Pocket Park Initiatives in China 27

C-6. China's Three-North Shelter Forest Program (The Green Great Wall) 30

D. Best practice and sub-national cases in China **31**

D-1. City-specific model linking weather and health data for better preparation of health risks 31

D-2. 15-minute life circle in Meishan and Shanghai 33

D-3. Panan's practice on combining environmental meteorological resources, traditional Chinese medicine, wellness tourism into integrated medical service and elderly care. 35

D-4. Dual-purpose infrastructure development in Beijing to enhance urban resilience to climate change 38

Table S1. Characteristics of the included policy documents. **40**

Reference **58**

A. Glossary for key words mentioned in included policy documents.

Chinese Terms	English Terms	Definition and Description
爱国卫生运动	Patriotic health campaign	The Patriotic Health Campaign is a mass health movement launched by the People's Republic of China starting in the 1950s. It is a key component of the country's health efforts and represents a successful application of the Communist Party's mass line in public health and disease prevention work.
安宁疗护	Palliative care	"Palliative care" refers to specialized medical care focused on providing relief from the symptoms, pain, and stress of serious illness, with the goal of improving the quality of life for both patients and their families. This type of care is provided alongside curative treatment or as the main approach when curative options are no longer viable. It addresses physical, emotional, and psychological needs, and often includes support for end-of-life care.
长期照护	Long-term care	"Long-term care" refers to a range of services and support designed to meet the needs of individuals who have chronic illnesses, disabilities, or other conditions that require extended care over an extended period. This can include assistance with daily activities (such as bathing, dressing, and eating), medical care, and other supportive services. Long-term care can be provided in various settings, including home care, assisted living facilities, nursing homes, and specialized care institutions.
城市更新	Urban renewal	"Urban renewal" refers to planned redevelopment activities in areas of a city that are no longer suited to the demands of modern urban life. People living in cities often have various expectations and dissatisfactions with their housing, surrounding environment, transportation, shopping, entertainment, and other aspects of daily life. They call for timely repair and renovation of their homes, as well as improvements to streets, parks, green spaces, and disadvantaged residential areas, in order to create a comfortable living environment and a beautiful cityscape. All activities aimed at achieving these goals are part of urban renewal. Urban renewal involves the restoration, improvement, and optimization of urban spatial forms and functions within developed areas of central cities. It aims to comprehensively enhance the use of buildings, municipal facilities, public infrastructure, and more, while promoting industrial restructuring, environmental quality, and cultural heritage preservation.
城市基础设施建设和无障碍化	Urban infrastructure construction and accessibility / Urban infrastructure	"Urban infrastructure development and barrier-free accessibility" refers to the planning and construction of essential city facilities and systems, ensuring they are accessible to all individuals, including those with

	development and barrier-free accessibility	disabilities or limited mobility. This includes creating and upgrading infrastructure such as roads, public transportation, sidewalks, buildings, and public spaces to remove physical barriers and ensure that everyone can navigate and use these facilities safely and comfortably. The goal is to promote inclusivity and equal access to urban services and amenities, improving the overall quality of life for all residents.
高龄	Advanced age	Individuals who are in the later stages of life, typically those aged 60 or 65 and older. The term is used when discussing aging populations, healthcare, and social services for the elderly. The definition may vary slightly depending on the cultural or medical context, but it generally denotes a stage where increased support and consideration for age-related issues are necessary.
公园城市	Park City	A "Park City" integrates urban and rural green space systems, park networks, and park-like ecological layouts as fundamental and preemptive elements in urban and rural development. It emphasizes the provision of more high-quality ecological products to meet people's growing needs for a beautiful environment, creating a new urban-rural living environment that promotes a better life. The park city represents an advanced form of urban development and is an important pathway and distinct expression of urban modernization.
国家老年疾病临床医学研究中心	National Clinical Research Center for Geriatric Diseases	The "National Clinical Research Center for Geriatric Diseases" is a research institution officially approved by the former National Health and Family Planning Commission. Its goal is to promote the establishment of the National Geriatric Medical Center and to advance the development of geriatric medicine more effectively and rapidly.
海绵城市	Sponge city	A sponge city is a concept for modern urban stormwater management, which refers to cities that have good 'resilience' in adapting to environmental changes and dealing with natural disasters caused by rainfall. It is also known as a "water-resilient city" The internationally recognized term is "low-impact development (LID) stormwater systems" This approach involves absorbing, storing, infiltrating, and purifying rainwater during rainfall, and releasing and utilizing the stored water when needed.
环境卫生	Environmental health / sanitation	"Environmental health" refers to the branch of public health focused on understanding and managing the impact of environmental factors on human health. This includes studying how air, water, soil quality, and other environmental conditions affect health outcomes and implementing measures to protect and improve environmental conditions to promote public health. Environmental health covers a wide range of issues such

		as pollution control, waste management, safe drinking water, and reducing exposure to harmful substances. In policy and public health discussions, environmental health is essential for creating safe and healthy living environments and preventing health issues related to environmental factors.
环境友好	Environment-friendly	"Environmentally friendly" refers to practices, products, or processes that have minimal negative impact on the environment. This includes reducing pollution, conserving natural resources, minimizing waste, and using sustainable materials and methods. Environmentally friendly approaches aim to protect and preserve the natural environment, promote ecological balance, and contribute to the health and well-being of both current and future generations. In various contexts, being environmentally friendly is crucial for fostering sustainability and reducing ecological footprints.
家庭病床	Home sick-bed / Family sick-bed	A "home / family sick-bed" refers to a specialized bed provided in a patient's home to offer a hospital-like environment for those requiring extended medical care or recovery. These beds are designed to provide comfort, support, and accessibility for patients with significant medical needs, such as those recovering from surgery or managing chronic conditions. They often come with adjustable features to assist with positioning and care.
减缓	Mitigation	"Mitigation" refers to actions or strategies aimed at reducing the severity or impact of a problem or risk. In various contexts, it involves efforts to lessen the adverse effects of an issue, such as climate change, environmental degradation, or health risks. For example, climate change mitigation includes measures like reducing greenhouse gas emissions and transitioning to renewable energy sources. In general, mitigation focuses on preventing or minimizing damage to achieve better outcomes and improve resilience.
健康老龄化	Healthy aging	"Healthy aging" refers to the process of maintaining good physical, mental, and social health as one grows older. It emphasizes the importance of adopting lifestyle practices and receiving care that promote well-being and quality of life throughout the aging process. Healthy aging includes aspects such as regular exercise, balanced nutrition, mental stimulation, social engagement, and preventive healthcare to manage chronic conditions and prevent decline.
健康中国建设	Healthy China Construction / Initiative	The "Healthy China Initiative" refers to a comprehensive national strategy aimed at improving public health and enhancing the overall well-being of the population in China. This initiative encompasses a range of policies and programs focused on disease prevention, health promotion, access to healthcare services, and the

		integration of health considerations into various aspects of public policy and social development. The goal is to build a healthier society by addressing key health challenges, promoting healthy lifestyles, and ensuring equitable access to medical resources and services. In international contexts, similar initiatives are recognized as crucial for advancing public health and achieving sustainable development goals.
居家护理	Home nursing	"Home nursing" refers to professional nursing services provided in a patient's home. This type of care includes medical tasks such as administering medications, wound care, monitoring vital signs, and managing chronic conditions. Home nursing care is often part of a broader home care plan and is typically provided by registered nurses or specialized nursing professionals. This approach allows patients to receive high-quality medical care in a familiar and comfortable environment, which can be particularly beneficial for those with chronic illnesses or recovering from surgery.
居家适老化改造	Home modification for the elderly / Age-Friendly Home Modification	"Age-friendly home modification" refers to the process of adapting and remodeling a home to make it more accessible and safer for older adults. This includes changes such as installing grab bars, improving lighting, modifying bathroom fixtures, and eliminating tripping hazards to accommodate the physical and functional changes associated with aging. The goal is to enhance the safety, comfort, and independence of elderly residents, allowing them to remain in their homes longer.
居家医疗服务	Home-nursing services	"Home-nursing services" refer to healthcare professionals from medical institutions providing door-to-door services for specific groups, with a focus on elderly patients. These services include diagnosis and treatment, medical care, rehabilitation therapy, pharmaceutical services, palliative care, and traditional Chinese medicine services, in accordance with relevant requirements.
居家照护	Home care	"Home care" refers to a range of services provided to individuals in their own homes to assist with daily living activities and medical needs. This can include personal care, such as bathing and dressing, as well as medical services like medication management and physical therapy. Home care is often used by elderly individuals or those with chronic conditions who prefer to remain in their own homes rather than move to a healthcare facility.
康复	Recovery / Rehabilitation	"Rehabilitation" refers to a range of services and therapies aimed at helping individuals recover or improve their physical, mental, and functional abilities following illness, injury, or surgery. Rehabilitation may include physical therapy, occupational therapy, speech therapy, and psychological support, depending on the specific needs of the individual. The goal is to restore the highest

		possible level of independence and quality of life.
可持续	Sustainable, sustainability	"Sustainability" is the process of maintaining environmental balance and harmony in resource development, investment direction, technological advancement, and institutional change while meeting human needs and ensuring future development. Sustainability can be an idea, a characteristic of a living system, a method of production, or a way of life.
口袋公园	Pocket Park	Pocket parks, also known as "mini-parks", are small-scale urban open spaces, often scattered or hidden within the city's structure, directly serving local residents. They can offer functions such as beautification, public interaction, recreation, and cultural display. Pocket parks can take the form of parks, plazas, or green spaces attached to various types of urban development land. They represent the revitalization and enhancement of unused or repurposed spaces within the city. Their typical characteristics include small size, specialized functions, close proximity, flexible space, and high efficiency, distinguishing them from traditional urban parks.
老龄	Aging	The demographic trend where the proportion of elderly individuals in a population increases, typically due to longer life expectancy and lower birth rates.
老年	Older / Older people / Elderly	The later stages of an individual's life, typically associated with retirement and aging-related challenges. The specific age range for "old age" may vary, but it generally refers to people aged 65 and above.
老年痴呆防治	Prevention and treatment of dementia	"Prevention and management of dementia" refers to a range of strategies and interventions aimed at reducing the risk of dementia and managing its progression once it occurs. This includes preventive measures such as lifestyle modifications, cognitive training, and early detection through screening. For those already diagnosed, management involves medical treatment, cognitive therapies, support services, and caregiving strategies to improve quality of life and maintain cognitive function.
老年口腔健康	Elderly oral health	"Elderly oral health" refers to the maintenance and care of dental and oral hygiene specific to older adults. This includes the prevention and treatment of common oral issues that affect seniors, such as tooth decay, gum disease, and oral cancers. It also encompasses practices to maintain healthy teeth and gums, manage dentures or other dental prosthetics, and address difficulties related to aging, such as dry mouth or changes in taste.
老年医学科	Geriatrics Department	The Geriatrics Department is specifically designed for individuals aged 60 and above. It functions as a comprehensive department encompassing general medicine, including the treatment of acute illnesses, disease prevention and management, long-term care, and end-of-life care. It integrates health maintenance, medical

		care, and research into a unified multidisciplinary field
老年营养改善	Elderly nutrition improvement	"Elderly nutrition improvement" refers to efforts and strategies aimed at enhancing the dietary intake and nutritional status of older adults. This involves ensuring that elderly individuals receive a balanced diet that meets their specific health needs, which may include managing chronic conditions, preventing malnutrition, and promoting overall health and well-being. Nutritional improvement can include tailored meal plans, dietary supplements, and education on healthy eating practices.
老年友好型医疗机构	Age-friendly medical institutions	An "age-friendly medical institutions" refers to medical institutions designed to meet the specific needs of elderly patients. These facilities incorporate features and practices that accommodate the physical, cognitive, and emotional requirements of older adults. This may include accessible design, specialized medical services, staff trained in geriatric care, and programs that promote senior well-being.
老年友善医疗卫生机构	Age-friendly medical and health institutions	An "age-friendly medical and health institutions" refers to a medical and health care establishment that is designed to cater specifically to the needs of elderly patients. Including general hospitals, rehabilitation hospitals, nursing homes and primary medical institutions to provide medical services for the elderly. This includes features and practices that enhance accessibility, comfort, and safety for older adults. Such facilities integrate specialized services for aging populations, including geriatric care, preventive health measures, and support for chronic conditions. The aim is to create an environment where elderly patients receive appropriate and compassionate care, promoting their overall well-being and quality of life.
老年专科	Geriatrics / Geriatric Specialization	"Geriatrics refers to the branch of medicine that focuses on the healthcare of elderly people. This field addresses the unique medical needs and challenges associated with aging, such as chronic diseases, mobility issues, cognitive decline, and the management of multiple medications.
老年综合征	Geriatric syndrome	"Geriatric syndrome" refers to a range of clinical manifestations or issues in older adults caused by multiple diseases or factors. It is not a specific disease but a collective term for various clinical syndromes unique to the elderly. Common geriatric syndromes include falls, dementia, urinary incontinence, delirium, insomnia, depression, sarcopenia, frailty, polypharmacy, and chronic pain, among others. The incidence and prevalence of geriatric syndromes increase with age. With the aging population, the number of elderly individuals affected by these syndromes is substantial.
老人	Elderly（Individuals）	An individual who has reached an advanced stage of life,

		generally 65 years or older. Senior citizens are often considered a vulnerable group that requires special attention in terms of rights, access to services, and protection in various societal contexts.
临终关怀	Hospice care / Palliative care / End-of-Life care	"End-of-life care" refers to the support and medical care provided to individuals who are in the final stages of a terminal illness or are approaching death. This type of care focuses on ensuring comfort, dignity, and quality of life for patients, while also supporting their families. It includes pain and symptom management, emotional and psychological support, and assistance with decision-making and legacy issues.
绿色	Green	Ecological civilization construction is the process of integrating and mutually enhancing ecological sustainability with economic and social development. The consumption rate of renewable resources should not exceed their regeneration rate, and the consumption rate of non-renewable resources should not surpass the regeneration rate of renewable resources that can replace them. The emission of pollutants should not exceed the natural environment's capacity for self-purification. Green is the fundamental color of nature, representing the value of nature itself.
绿色低碳发展	Green and low-carbon development	"Green and low-carbon development" refers to reducing high-carbon energy consumption and lowering greenhouse gas emissions through technological innovation, institutional innovation, industrial transformation, and new energy development, all under the guidance of sustainable development principles. The goal is to achieve a win-win situation between economic and social development and ecological environmental protection. Green low-carbon industries play a crucial role in driving the green transformation of development models by providing other industries with green low-carbon products and services.
绿色空间建设	Green space construction	"Green space construction" refers to the planning, creation, and enhancement of areas that are dedicated to natural vegetation and open spaces within urban and rural environments. This includes parks, gardens, greenways, and natural reserves. The objective is to improve environmental quality, provide recreational opportunities, support biodiversity, and enhance the overall well-being of communities. In urban planning and environmental policy, green space development is crucial for promoting sustainable living, improving air quality, and fostering a connection between people and nature.
慢性病全程防治管理服务	Comprehensive prevention， treatment， and management service	"Comprehensive management and care services for chronic diseases" refer to a holistic approach to managing chronic conditions throughout their entire course. This includes prevention, early detection, ongoing treatment,

	for chronic diseases / Comprehensive management and care services for chronic diseases	and regular monitoring of chronic diseases such as diabetes, hypertension, and heart disease. The approach aims to improve patient outcomes by integrating various aspects of care, including medical treatment, lifestyle changes, patient education, and coordination of services.
美丽中国建设	Beautiful China Construction / Initiative	Adhere to the concept that humans and nature are a shared life community, create an ecological environment with green mountains and clear waters, build a comfortable and livable living environment, and allow the people to share in the beauty of nature, life, and living.
"平急两用"公共基础设施	Dual-purpose infrastructure	"Dual-purpose infrastructure" refers to public infrastructure designed to serve both everyday needs and emergency situations. It means that facilities and systems are built to function effectively under normal conditions while also being adaptable for use during emergencies or crises. This approach ensures that public services and resources remain functional and effective, whether for routine use or in times of disaster.
气候变化	Climate change	After a considerable period of observation, the climate changes caused by human activities that directly or indirectly alter the global atmospheric composition, in addition to natural climate variability.
气候变化适应	Climate change adaptation	"Climate change adaptation" is the process of adjusting to current or anticipated impacts of climate change. For humans, the goal of adaptation is to reduce or avoid harm while also finding and taking advantage of opportunities. Humans can assist adaptation to natural systems through various interventions. Different methods, strategies, and options for responding to climate change impacts already exist around the world. Adaptation practices include four areas: infrastructure and technology, institutional aspects, behavior and culture, and nature-based approaches.
韧性城市/适应型城市/弹性城市	Resilient city	In a broad sense, a resilient city refers to an urban area that can quickly respond to and maintain the basic functions of economic, social, infrastructure, and resource systems during sudden "black swan" events such as economic crises, public health emergencies, earthquakes, floods, fires, wars, and terrorist attacks. Additionally, it possesses the capability to recover rapidly and achieve a safer state after the impact has ended.
三北防护林工程/绿色长城	The Three-North Shelter Forest Program / Green Great Wall	"The Three-North Shelter Forest Program" refers to a large-scale afforestation ecological project in the Three-North regions of China (Northwest, North, and Northeast). Building upon the protection of existing forest and grassland vegetation, methods such as artificial afforestation, aerial seeding, and forest and grass restoration through the closure of hillsides and sand dunes are employed. The project establishes windbreak

		and sand-fixation forests, soil and water conservation forests, farmland shelter forests, pasture shelter forests, as well as fuelwood and economic forests. It creates a protective forest system that integrates trees, shrubs, and grasses, combining forest belts, forest networks, and forest patches, with rational arrangements of various forest types and tree species, promoting coordinated development of agriculture, forestry, and animal husbandry.
森林城市	Forest City	A "Forest City" refers to a healthy and stable forest ecosystem dominated by forests and trees within the administrative boundaries of a city, integrating both urban and rural areas. It meets certain standards in areas such as forest networks, forest health, ecological benefits, and ecological culture, thereby achieving harmonious development between humans and nature.
森林康养	Forest health preservation / Forest therapy / Forest health care	"Forest health care" refers to utilizing high-quality forest resources to organically combine modern medicine with traditional Chinese medicine. It involves equipping forest areas with corresponding health, leisure, medical, and rehabilitation facilities, and offering a range of activities aimed at improving personal well-being, adjusting physical functions, and delaying aging. These activities include forest recreation, vacationing, therapy, health preservation, and elderly care, all designed to benefit human physical and mental health.
上门巡诊	Home visits for medical consultations / visitation	"Home visits for medical consultations / visitation" refers to a healthcare service where medical professionals, such as doctors or nurses, visit patients at their homes to provide medical care, consultations, or routine check-ups. This service is often used for patients who are elderly, disabled, or have chronic conditions that make it difficult for them to visit a healthcare facility. Home visit consultations ensure that these patients receive the necessary medical attention in the comfort of their own homes, improving access to healthcare and supporting patient well-being.
生态修复	Ecological restoration	"Ecological restoration" refers to using the self-repair capabilities of ecosystems, or implementing appropriate human-assisted measures, to gradually restore the stability of degraded, damaged, or destroyed natural ecosystems. The goal is to achieve a stable and healthy ecosystem that is either self-sustaining or requires minimal human assistance for maintenance. Measures for ecological restoration include designating ecological redlines, developing reasonable regional development patterns, and adjusting land use directions and layouts.
适老	Fit for the older / Age-friendly / Elder-friendly	"Age-friendly" refers to environments, products, services, or policies designed to accommodate the needs of older adults. This concept is often applied to urban planning,

		housing, healthcare, and public services to ensure they are accessible and comfortable for the elderly.
失能预防及干预	Disability prevention and intervention	"Disability prevention and intervention" refers to strategies and measures aimed at reducing the risk of disabilities and managing or mitigating their impact. This includes a range of activities such as early detection of health issues, implementation of preventive measures to avoid disability, and providing timely interventions to manage existing disabilities. It encompasses medical, social, and rehabilitative approaches to help individuals maintain their functional abilities and quality of life.
15 分钟基本卫生医疗服务圈	15-minute basic medical service coverage	The "15-minute basic medical service coverage" refers to a healthcare delivery model where basic medical services are designed to be accessible within a 15-minute distance from a person's home. This model aims to ensure that essential healthcare services, including preventive care, treatment of common illnesses, and emergency care, are readily available to all individuals within a short travel time. The goal is to improve access to healthcare, reduce barriers to service utilization, and promote equitable health outcomes.
15 分钟生活圈	15-minutes life circle	A "15-minutes life circle" refers to a basic unit serving a population of 50,000 to 100,000 people, where within a 15-minute walking distance from home, residents can access essential public services as much as possible, such as elderly care, healthcare, education, commerce, transportation, and cultural and sports facilities.
适应	Adaptation	"Adaptation" refers to the alignment of biological structures at various levels (from macromolecules, cells, tissues, and organs, to populations composed of individuals) with their functions. On the other hand, this structure-function relationship (including behaviors and habits) is suited to the survival and persistence of the organism under specific environmental conditions."
施治	Treatment / Administration	"Treatment or administration" refers to the process of applying or implementing therapeutic interventions to address a medical condition or health issue. This encompasses various methods and techniques used by healthcare professionals to treat patients, including medication, surgical procedures, physical therapy, and other forms of medical care. The goal is to alleviate symptoms, cure diseases, or manage chronic conditions effectively, based on the diagnosis and clinical needs of the patient.
数字化国家级老年健康教育科普资源库	Digital national health education and science popularization resource library for the elderly / Digital	The "Digital national health education and science popularization resource library for the elderly" refers to a comprehensive online repository that provides educational materials and information focused on elderly health. This resource library includes digital content such as research, guidelines, educational programs, and public

	national resource library for elderly health education and public awareness	awareness campaigns designed to support the health and well-being of older adults. The aim is to offer accessible and reliable information to both healthcare professionals and the general public, promoting informed decision-making and effective management of elderly health issues.
太极拳、八段锦、五禽戏	Tai Chi<br>Baduanjin<br>Wuqinxi（five-animal boxing）	Tai Chi is a form of boxing in Chinese martial arts. Ba Duan Jin (Eight Pieces of Brocade) is a fitness method invented in ancient China, consisting of eight different body movements that include both physical exercises and breath regulation. The Five Animal Frolics include movements imitating the tiger, deer, bear, monkey, and bird, each designed to emulate the actions of these animals.
碳中和	Carbon neutrality	"Carbon neutrality" it refers to the total amount of carbon dioxide or greenhouse gas emissions directly or indirectly produced by a country, company, product, activity, or individual over a certain period. This is achieved through measures such as afforestation and energy conservation to offset the emissions generated, achieving a balance between emissions and offsets to reach a relative "zero emissions" status.
体医融合	Integration of sports and medicine	Integration of sports and medicine refers to the organic combination of sports methods and modern medical concepts and technologies. It involves scientifically incorporating elements of physical exercise into various stages of medical care. This integration not only reflects the mutual learning and application of techniques between the two fields but also demonstrates a close fusion and promotion of ideas and theories across multiple levels.
完整社区	Comprehensive community	A "comprehensive community" refers to a residential area where, within a walkable distance for residents, there are well-established basic public services, comprehensive commercial services, adequate municipal infrastructure, sufficient public activity spaces, full coverage of property management, and a sound community management mechanism. It is also a community where residents have a strong sense of belonging and identity. Residential communities are the fundamental units of urban life and governance.
无障碍环境建设	Barrier-free environment construction / Accessible environment construction	"Barrier-free environment construction" refers to creating conditions that enable people with disabilities and the elderly to independently and safely navigate roads, access buildings and their facilities, use public transportation, obtain and use information, and access social services. It is a crucial aspect of safeguarding the rights of these groups and holds significant value in promoting equitable access to the benefits of economic and social development for all.

学科诊疗模式	Discipline-based diagnosis and treatment model	The "discipline-based diagnosis and treatment model" refers to a healthcare approach where medical treatment and diagnosis are organized around specific medical disciplines or specialties. Each discipline focuses on particular areas of health, utilizing specialized knowledge and techniques to diagnose and treat conditions effectively. This model often involves collaboration across various specialties to provide comprehensive care, ensuring that complex health issues are approached from multiple angles for more effective patient outcomes. It emphasizes the importance of expertise within each medical field to enhance precision and efficiency in treatment.
养老	Elderly care	"Elderly care" refers to the services and support provided to older adults, particularly those who may need assistance with daily living activities due to aging. This can include in-home care, community-based services, or residential care facilities. In diplomatic and policy discussions, it is a key issue as countries address the needs of their aging populations, including healthcare, long-term care insurance, and support for caregivers. The term encompasses a wide range of services, from medical care to social and emotional support.
养生保健	Health preservation and nourishment	Health preservation activities guided by theory aim to enhance physical fitness, prevent diseases, and extend lifespan through various methods.
药膳食疗	Dietary therapy with medicinal herbs	"Dietary therapy with medicinal herbs" refers to adding traditional Chinese medicinal herbs to food with the goal of treating diseases, a concept introduced by Luo Li. This approach integrates medicinal herbs into food to achieve therapeutic effects. It is known as medicinal cuisine because it incorporates herbs into meals, forming a unique practice in the health field. It differs from conventional drug therapy and is distinct from ordinary dietary practices.
宜居	Livable	"Livable" refers to the quality of an environment or place that makes it suitable and pleasant for people to live in. This includes factors such as safety, comfort, accessibility, cleanliness, and the availability of essential services and amenities. In urban planning and community development, creating livable spaces is essential for ensuring a high quality of life for residents and fostering vibrant, healthy communities.
医疗照护	Medical care	"Medical care" refers to the range of services provided by healthcare professionals to diagnose, treat, and manage health conditions and illnesses. This encompasses preventive care, acute treatment, chronic disease management, and rehabilitation. Medical care includes various services such as consultations with physicians, diagnostic tests, surgeries, medication management, and

		therapeutic interventions.
医养结合	Integration of medical treatment and elderly support	"Integration of medical treatment and elderly support" refers to a coordinated approach that combines healthcare services with long-term care for the elderly. This model ensures that older adults receive both medical treatment and daily living support in one comprehensive system, often within the same facility or through closely linked services.
预防保健	Preventive health care	"Preventive health care" refers to measures and practices aimed at preventing the onset of diseases and maintaining overall health and well-being. This includes activities such as regular health screenings, vaccinations, health education, and lifestyle modifications to reduce the risk of illness. The goal of preventive health care is to identify and address potential health issues before they develop into more serious conditions, thereby improving long-term health outcomes and reducing healthcare costs.
园林城市	Garden City	A "Garden City" is a city selected based on the 'National Garden City Standards' issued by the Ministry of Housing and Urban-Rural Development. It features balanced distribution, reasonable structure, comprehensive functions, beautiful landscapes, and a fresh, comfortable, and safe living environment. It is one of the important city brands.
整合型医疗卫生服务体系	Integrated health service system	"Integrated health service system" refers to a coordinated approach that combines various aspects of health care and medical services into a unified framework. This system aims to provide seamless and efficient care by integrating primary care, specialty care, hospital services, and public health initiatives. The goal is to improve patient outcomes, enhance service delivery, and ensure that individuals receive comprehensive care across different levels of the health system.
中医体质辨识	The recognition of TCM constitution	The "recognition of TCM constitution" refers to the process of recognizing and understanding an individual's constitution, focusing on the characteristics of different constitutions and their implications for health and disease. By assessing the overall factors and individual differences in constitution, TCM practitioners develop preventive and treatment principles, and select appropriate methods for treatment, prevention, and health maintenance, thereby implementing 'personalized' interventions.
中医药养生养老	Traditional Chinese Medicine (TCM) for health preservation and elderly care	"Traditional Chinese Medicine (TCM) for health preservation and elderly care" refers to the use of TCM principles and practices to promote health, prevent disease, and manage the well-being of elderly individuals. This approach includes techniques such as acupuncture, herbal medicine, dietary recommendations, and tai chi, tailored to the specific needs of older adults.

		The aim is to support longevity, enhance quality of life, and address age-related health issues through holistic and preventive measures rooted in traditional Chinese medical philosophy.
中医治未病中心	TCM preventive treatment center	The "Traditional Chinese Medicine Preventive Care Center" focuses on the prevention of diseases by applying the principles of Traditional Chinese Medicine (TCM). The concept of "治未病" (Zhi Wei Bing) emphasizes early intervention and maintaining balance in the body to prevent the onset of illness. These centers provide health assessments, lifestyle guidance, herbal treatments, acupuncture, and other TCM therapies aimed at strengthening the body's defenses and promoting overall well-being, helping individuals avoid disease before symptoms appear.
住宅适老化改造	Residential modification for the elderly	"Residential modification for the elderly" refers to the process of adapting and renovating residential spaces to make them safer, more accessible, and more comfortable for elderly individuals. This includes modifications such as installing grab bars, improving lighting, adjusting bathroom fixtures, and removing tripping hazards. The goal is to enhance the functionality of the home and support the independence and quality of life of older adults, allowing them to live comfortably and safely in their own homes as they age. In policy and urban planning, age-friendly home modifications are essential for supporting aging populations and promoting inclusive living environments.
资源节约	Resource-saving	Resource conservation has two meanings: first, it refers to reducing the absolute amount of resource consumption relative to waste; second, it involves improving resource utilization efficiency through a combination of legal, economic, technological, and administrative measures to achieve the maximum economic and social benefits with the least resource consumption.

62

B. Search Strategy.

B-1. List of search databases.

The policy source of departments affiliated to the State Council of China			
**ID**	**Government administration**	**Name in Chinese**	**URL of website**
1	General Office of the State Council	国务院办公厅	http://www.gov.cn/guowuyuan/
2	Ministry of Foreign Affairs	外交部	https://www.fmprc.gov.cn/web/
3	Ministry of National Defense	国防部	http://www.mod.gov.cn/
4	National Development and Reform Commission	国家发展和改革委员会	http://www.ndrc.gov.cn/
5	Ministry of Education	教育部	http://www.moe.gov.cn/
6	Ministry of Science and Technology	科学技术部	http://www.most.gov.cn/
7	Ministry of Industry and Information Technology	工业和信息化部	www.miit.gov.cn
8	National Ethnic Affairs Commission	国家民族事务委员会	http://www.seac.gov.cn/
9	Ministry of Public Security	公安部	http://www.mps.gov.cn/
10	Ministry of Civil Affairs	民政部	http://www.mca.gov.cn/
11	Ministry of Justice	司法部	http://www.moj.gov.cn
12	Ministry of Finance	财政部	http://www.mof.gov.cn/index.htm
13	Ministry of Human Resources and Social Security	人力资源和社会保障部	http://www.mohrss.gov.cn/
14	Ministry of Natural Resources	自然资源部	http://www.mnr.gov.cn/
15	Ministry of Ecology and Environment	生态环境部	http://www.mee.gov.cn/
16	Ministry of Housing and Urban-Rural Development	住房和城乡建设部	http://www.mohurd.gov.cn/

17	Ministry of Transport	交通运输部	http://zs.mot.gov.cn/so
18	Ministry of Water Resources	水利部	www.mwr.gov.cn/
19	Ministry of Agriculture and Rural Affairs	农业农村部	http://www.moa.gov.cn/
20	Ministry of Commerce	商务部	http://www.mofcom.gov.cn/
21	Ministry of Culture and Tourism	文化和旅游部	https://www.mct.gov.cn/
22	National Health Commission	国家卫生健康委员会	http://www.nhc.gov.cn/
23	Ministry of Veterans Affairs	退役军人事务部	http://www.mva.gov.cn/
24	Ministry of Emergency Management	应急管理部	http://www.chinasafety.gov.cn/
25	The People’s Bank of China	中国人民银行	http://www.pbc.gov.cn/
26	National Audit Office	审计署	http://www.audit.gov.cn/

B-2. List of pre-specific Chinese keywords used in searching process.

Each ministry’s website was searched using specific Chinese keywords:

- **Age-friendly:** 老年 (Lao Nian), 老龄 (Lao Ling), 老人 (Lao Ren), 高龄 (Gao Ling), 养老 (Yang Lao), 适老 (Shi Lao), 宜居 (Yi Ju), 医养结合 (Yi Yang Jie He)
  Older, aging, elderly, advanced age, elderly care, elder-friendly, livable, integration of medical treatment and elderly support

- **Environmentally sustainable:** 可持续 (Ke Chi Xu), 气候变化 (Qi Hou Bian Hua), 海绵城市 (Hai Mian Cheng Shi), 韧性城市 (Ren Xing Cheng Shi), 弹性城市 (Tan Xing Cheng Shi), 健康城市 (Jian Kang Cheng Shi), 环境健康 (Huan Jing Jian Kang), 绿色 (Lv Se), 低碳 (Di Tan), 资源节约 (Zi Yuan Jie Yue), 环境友好 (Huan Jing You Hao), 碳中和 (Tan Zhong He)
  sustainable, climate change, sponge city, resilient city, healthy city, environmental health, green/greenness, low-carbon, resource-saving, environment-friendly, carbon neutrality

- **Cities and communities:** 社区 (She Qu), 城乡 (Cheng Xiang), 基础设施 (Ji Chu She Shi) community, urban and rural, infrastructure

C. Innovative programs and initiatives in China

C-1. Age-friendly demonstrated communities in China

Since 2009, the National Office on Aging（全国老龄办）has launched nationwide pilot programs to create "Elderly-Friendly Communities" and "Age-Friendly Cities". On December 9, 2020, the National Health Commission of China issued a notification titled "Notice on the Establishment of National Demonstration Age-Friendly Communities" [1], which outlines a strategic initiative to enhance the service capacity and quality of communities to better meet the needs of the elderly. This initiative aims to improve various aspects of elderly life, including living environments, daily transportation, health and eldercare services, social participation, and cultural life. The overarching goal is to develop a sustainable model for creating age-friendly communities and establish a long-term mechanism to significantly enhance the sense of gain, happiness, and security among the elderly. By 2025, the plan is to establish 5,000 demonstration age-friendly communities across urban and rural areas, with nationwide coverage targeted by 2035. The notification specifies six key tasks (Figure C1):

- Improving the living environment for the elderly.
- Facilitating daily transportation for seniors.
- Enhancing the quality of services provided to the elderly.
- Expanding social participation opportunities for the elderly.
- Enriching the cultural and spiritual life of the elderly.
- Increasing the level of technological support in services for the elderly.

To guide the creation of age-friendly communities, the “National Standards for Demonstration Age-Friendly Urban and Rural Communities (Trial)” [2] was introduced. This standard provides reference criteria for both urban and rural communities, covering seven key areas:

- Safe and clean living environment.
- Comprehensive and convenient transportation facilities.
- Accessible and convenient community services.
- Broad and active social participation.
- Strong cultural respect for the elderly.
- Technological innovation to support aging.
- Effective management and protection mechanisms.

This initiative underscores the commitment to fostering environments where the elderly can live comfortably, participate fully in social life, and enjoy a high quality of life through well-structured and supportive community systems.

Elderly-friendly communities are self-declared by the communities themselves and undergo a multi-level approval process. The final evaluation and naming are conducted by the National Health Commission (National Office on Aging), after comprehensive reviews and public announcements. Eight main aspects are focused on for both urban and rural areas when evaluation, including safe and

clean living environments, convenient and complete travel facilities, accessible community services, widespread social participation, a strong culture of filial piety and respect for the elderly, technological innovation to assist the elderly, and unique highlights.

The specific selection criteria and priorities of urban and rural areas are slightly different. For urban communities, they must meet 40 specific criteria, including ecological environment construction, fire prevention and emergency rescue networks, barrier-free housing, elevator installations, separate pedestrian and vehicular traffic on main roads, in-home medical services for disabled elderly, meal assistance services for the elderly, and caregiver training. While for rural communities, 30 specific criteria are pre-requisite, including household tap water, toilet renovations, daily garbage collection and disposal, clean rivers and canals, hardened main roads, lighting facilities, visits to elderly individuals in special difficulties, and services for left-behind elderly.

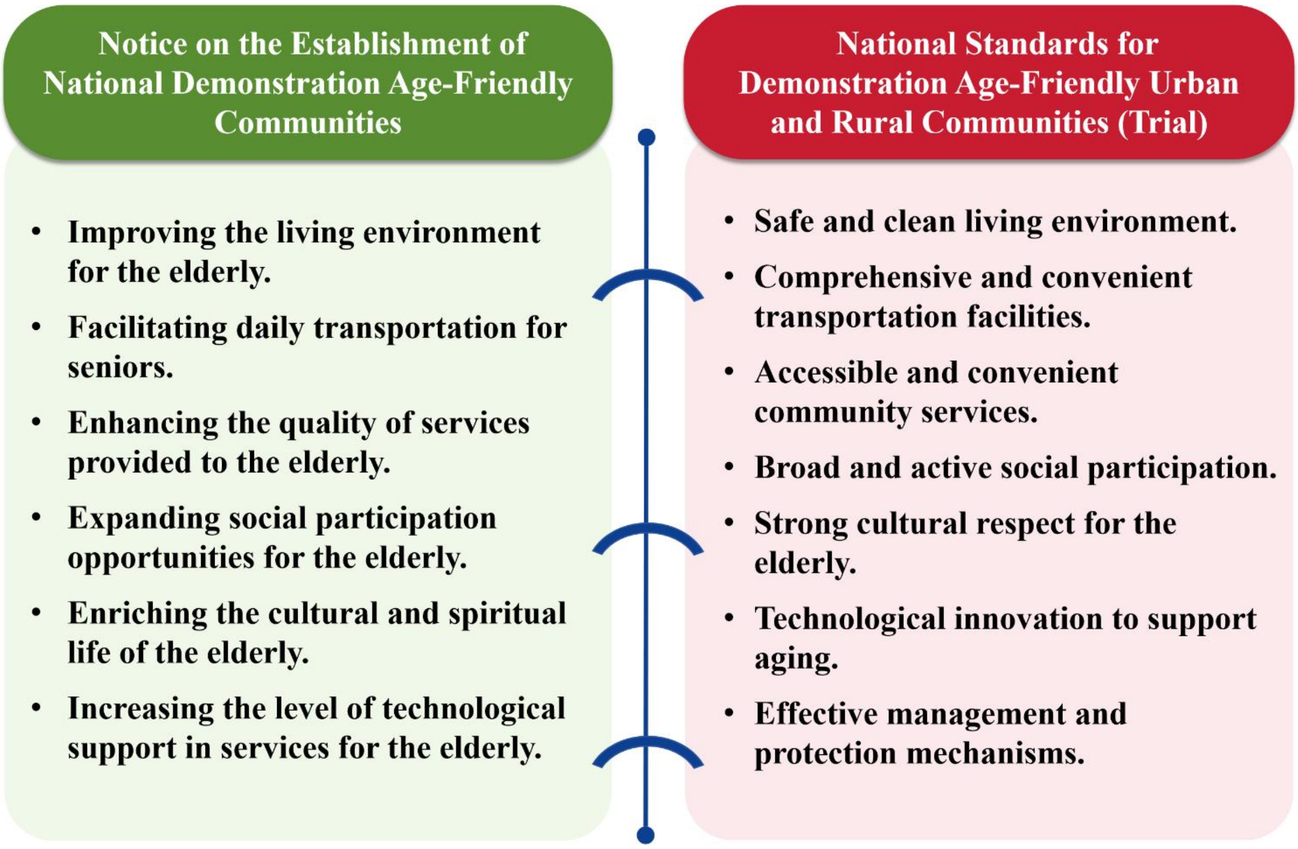

Figure C1. China's key policy document on building age-friendly and sustainable cities and communities covers key areas

As of now, there are 2985 elderly-friendly communities nationwide in total, covering 32 cities and provinces. For example, Yanwu Community in Xiamen (厦门市思明区滨海街道演武社区) is one of the demonstrative elderly-friendly communities which integrates commerce, tourism, religion, and education. It has eight residential areas with 1570 households and a population of 5550, including 1144 elderly people (21%). To make the community more elderly-friendly, a home-based elderly care service station (居家养老服务站) has been established, offering day care (日间照料) and lunch break (午休服务) services for the elderly. Additionally, an elderly activity center (老年人活动之家)

has been set up, equipped with books, Go tables, and fitness testing equipment. A fitness and recreation home (老年人健身康乐家园)has also been created to enrich the later life of elderly residents [3].

In addition, there are several measures to develop age-friendly communities in terms of transportation and daily living, including elderly transportation cards, subsidies and consumption vouchers, dining facilities, free access to public parks, and retrofitting of older residential areas. Simultaneously, the community has formed a leadership group dedicated to creating an elderly-friendly environment. The group has clearly defined responsibilities and has undertaken various initiatives, including renovating activity venues and facilities for the elderly, adding informational boards, and promoting the concepts of "active aging, healthy aging, and happy elderly". These efforts have significantly improved the well-being of the elderly population in the community.

C-2. Comprehensive Communities and Urban Renewal

"Urban renewal" (城市更新) in China has increasingly focused on creating age-friendly environments as part of the broader effort to build sustainable and livable cities. The concept of "comprehensive communities" has emerged as a key strategy in this context, supported by the Ministry of Housing and Urban-Rural Development. These communities are designed to provide residents with a full range of services and amenities within close proximity, promoting social inclusion and enhancing the quality of life, particularly for the elderly population.

One of the most significant recent initiatives is the pilot program for the construction of "comprehensive communities" (also known as "complete communities") [4]. Comprehensive communities are a recent urban development model in China aimed at meeting the diverse needs of residents in urban areas, particularly the elderly and other vulnerable groups. The core idea behind comprehensive communities is to create neighborhoods where residents can access a full range of essential services—such as housing, healthcare, education, recreation, and shopping—within walking distance, thereby enhancing the livability and sustainability of the community. There are some key features of the comprehensive communities:

- **Comprehensive Service Facilities:** Comprehensive communities are equipped with a full range of public services, including community hospitals, schools, commercial facilities, fitness centers, and cultural activity spaces. These facilities are not only designed to meet the daily needs of residents but are also strategically located to ensure convenience and accessibility. A standard comprehensive community includes a community service center with a police station, activity rooms, and a party group activity center, a kindergarten, a daycare center, an elderly service station, and a community health service station.

- **Green and Ecological Environment:** Emphasis is placed on creating an ecological environment with ample green spaces, parks, and walking paths, encouraging residents to engage in outdoor activities and promoting a healthy lifestyle. The design of these communities also focuses on barrier-free facilities, making them accessible to the elderly and people with disabilities. Public

activity spaces, including sports fields and public parks, are also integral, with specific areas dedicated to sports and recreation.

- **Social Service Network:** A well-developed social service system is an essential component of comprehensive communities. Community service centers within these neighborhoods offer a range of services, including elderly care, childcare, cultural and recreational activities, and psychological counseling, fostering interaction and support among residents. Property management services are also professionalized, ensuring that daily needs are met efficiently.

- **Smart Management:** With the advancement of smart city initiatives, comprehensive communities increasingly incorporate intelligent management systems, such as smart security systems, telemedicine services, and community network platforms, to improve the efficiency and quality of community management and services. A digital management platform often supports these features, facilitating online and offline community services.

- **Convenient Transportation:** Comprehensive communities prioritize convenient transportation, with well-planned public transit stops, bike lanes, and pedestrian networks, ensuring that residents can easily access other parts of the city. Parking and charging facilities for electric vehicles are also provided, reflecting the growing need for sustainable transport solutions.

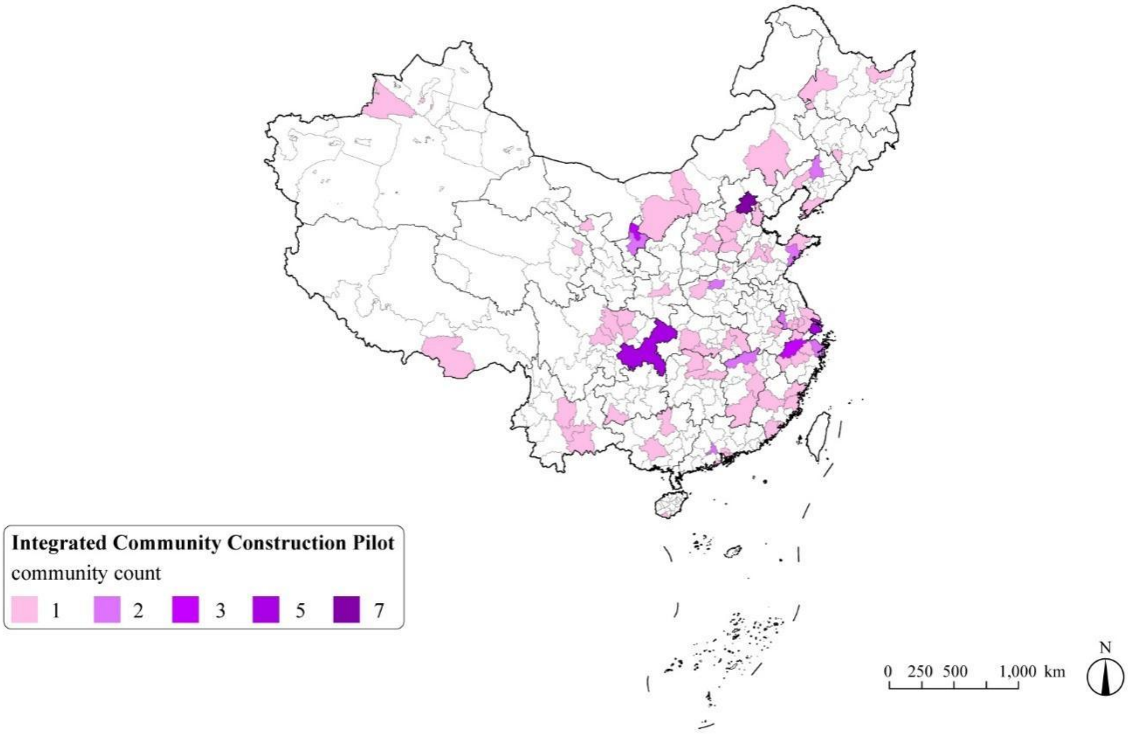

Figure C2. The distribution of "Pilot Communities of Comprehensive Community".

Several cities in China have begun piloting and promoting the development of comprehensive communities. In September 2020, Hebei Province inaugurated its first "Complete Community" in the city of Handan [5]. The Beigangyuan community, initially built in the 1980s, was revitalized and upgraded to meet the new standards. This project integrated various facilities, such as a community

supermarket, dining hall, cultural and sports centers, a neighborhood center, and an elderly care center. One of the standout features of this community is its smart management platform. The platform provides real-time monitoring of community activities, ensuring safety and efficiency. It includes smart surveillance, fire safety systems, healthcare monitoring, and smart access controls, making the community a model for modern urban living.

In Beijing, multiple communities are piloting the conversion of illegally constructed buildings into elder care centers, while integrating nearby commercial and medical resources to create a "15-minute life circle", meaning residents can meet all their basic living needs within a 15-minute walk. In Suzhou, Jiangsu Province, significant experience has been gained in building age-friendly communities, and successful cases have been compiled into books for other cities to reference.

Additionally, the widespread establishment of "elderly universities" and community service centers highlights the importance of social and recreational opportunities in these comprehensive communities. These institutions provide cultural and sports training, enriching the lives of the elderly, and are increasingly mandated to include dedicated spaces for elderly activities and social interaction. This holistic approach to urban renewal and community building is a crucial step in addressing the challenges posed by an aging population while promoting sustainability and inclusiveness in Chinese cities.

C-3. Pilot Project for Climate-Adaptive Cities

China's pilot project for Climate-adaptative cities is a critical part of the country's broader strategy to address the impacts of climate change on urban areas. Launched by the Ministry of Ecology and Environment (MEE) in collaboration with other governmental agencies, this initiative aims to enhance the resilience of cities to climate-related risks through systematic planning, policy innovation, and infrastructure development. The main objectives of the pilot project include:

- **Assessing Vulnerabilities:** Identifying the specific vulnerabilities of each pilot city to climate change, including risks such as flooding, heatwaves, droughts, and sea-level rise. This involves conducting detailed climate risk assessments to inform targeted adaptation measures.

- **Strengthening Urban Infrastructure:** Upgrading and adapting urban infrastructure to better withstand climate impacts. This includes improving drainage systems to handle increased rainfall, retrofitting buildings to cope with extreme temperatures, and enhancing green spaces to mitigate urban heat islands.

- **Enhancing Institutional Capacity:** Developing and strengthening local government capacity to implement climate adaptation policies. This involves training officials, creating dedicated climate adaptation departments, and integrating climate considerations into urban planning processes.

- **Promoting Sustainable Practices:** Encouraging cities to adopt sustainable practices that reduce their overall environmental impact, such as energy efficiency improvements, renewable energy integration, and sustainable water management.

- **Fostering Public Awareness and Engagement:** Engaging local communities in climate adaptation efforts by raising awareness of climate risks and promoting community-led initiatives. Public participation is encouraged to ensure that adaptation measures are socially inclusive and reflect the needs of all residents.

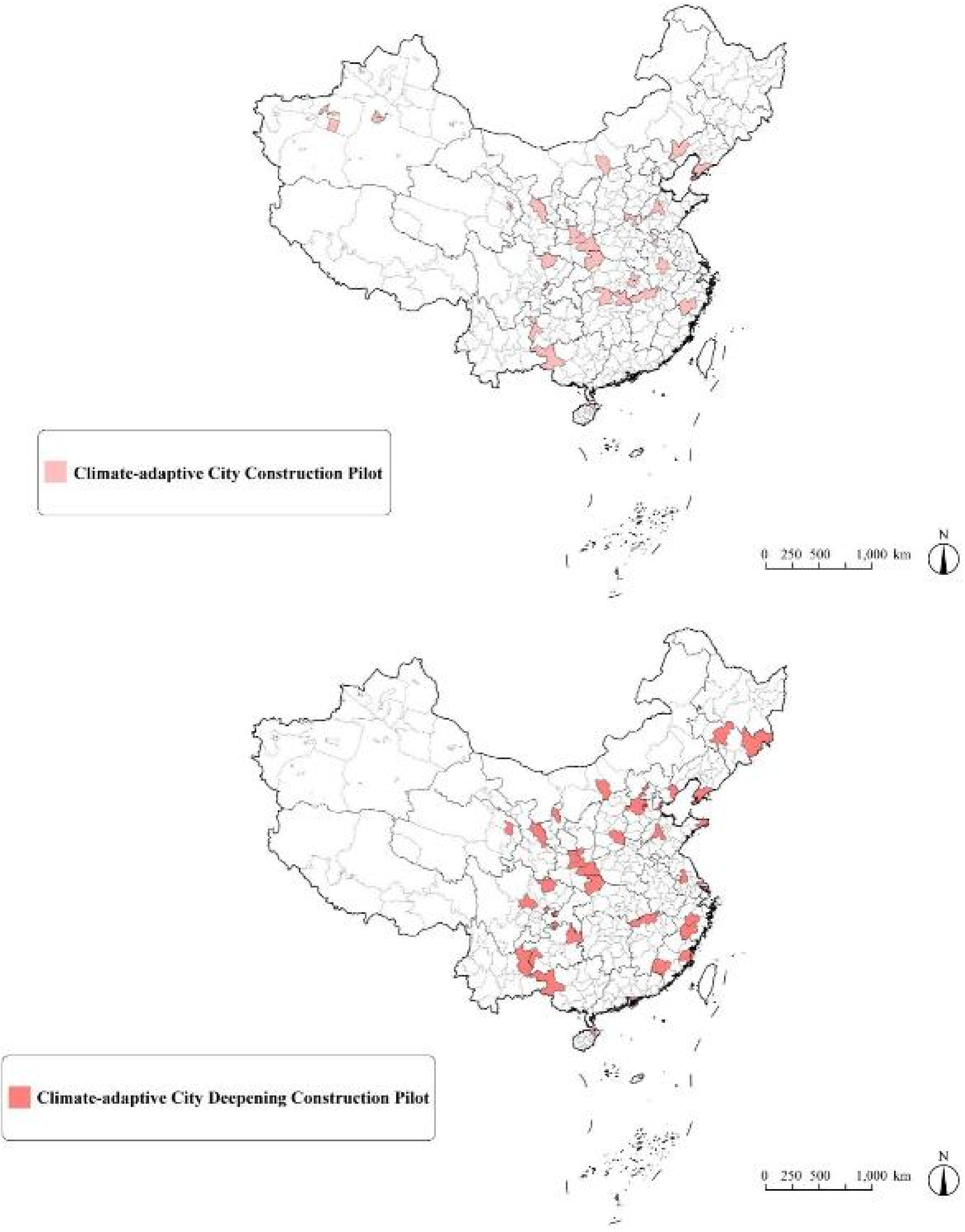

Figure C3. The distribution of "pilot projects of Climate-adaptive Cities" and "deepening pilot projects of Climate-adaptive Cities"

Several cities (28 cities in 2017 as "pilot projects of climate-adaptive cities" [6] and 39 cities in 2023 as "deepening pilot projects of climate-adaptive cities" [7]) across China have been selected as pilot cities, representing different geographic regions and climate zones. These include major urban centers like Shanghai, Guangzhou, and Shenzhen, as well as smaller cities that face unique climate challenges. Each pilot city is tasked with developing a comprehensive climate adaptation plan tailored to its specific risks and vulnerabilities.

Shanghai, for instance, has focused on enhancing its flood defenses due to its vulnerability to rising sea levels and increased storm surges. Guangzhou has prioritized improving its drainage systems and creating more green spaces to combat extreme heat. Meanwhile, cities in northern China, such as Harbin, have focused on adapting to changing patterns of snow and ice.

C-4. Sponge City Pilot and Demonstration Cities in China

The concept of a "Sponge City" (海绵城市) in China is part of a broader urban water management strategy designed to address issues of urban flooding, water scarcity, and environmental degradation. A Sponge City is designed to absorb, store, and purify rainwater like a sponge, allowing for natural infiltration and retention. The goal is to reduce surface runoff, mitigate urban flooding, and improve water quality while promoting sustainable urban development.

Sponge City Pilot Program

China launched the Sponge City Pilot Program [8] in 2015 as a response to increasing urban flooding and environmental concerns. An initial group of 16 pilot cities was selected in 2015, with an additional 14 cities selected the following year, based on their vulnerability to water-related issues and their readiness to implement innovative urban water management solutions. The cities selected were expected to integrate green infrastructure, such as permeable pavements, green roofs, rain gardens, and constructed wetlands, into their urban planning and design.

The primary objectives of the Sponge City Pilot Program were to enhance urban water resilience, improve water quality, and promote sustainable development. The program aimed to reduce the frequency and severity of urban flooding by improving cities' ability to absorb and manage rainwater more effectively. By filtering and purifying rainwater, sponge cities were also designed to help lower pollution levels in rivers and lakes. Additionally, the integration of green infrastructure was intended to foster healthier and more sustainable urban environments, benefiting both the residents and the surrounding ecosystems.

Sponge City Demonstration Cities

Following the initial pilot program, the Chinese government expanded the initiative to include "Sponge City Demonstration Cities" (海绵示范城市) [9]. These cities serve as examples of best practices in implementing sponge city principles on a larger scale. Demonstration cities are tasked with not only managing water more effectively but also showcasing how sponge city concepts can be integrated into broader urban development strategies.

These cities typically feature extensive green infrastructure, innovative water management technologies, and policies designed to promote sustainable urban growth. The demonstration cities are also intended to provide valuable insights and models that can be replicated in other urban areas across China.

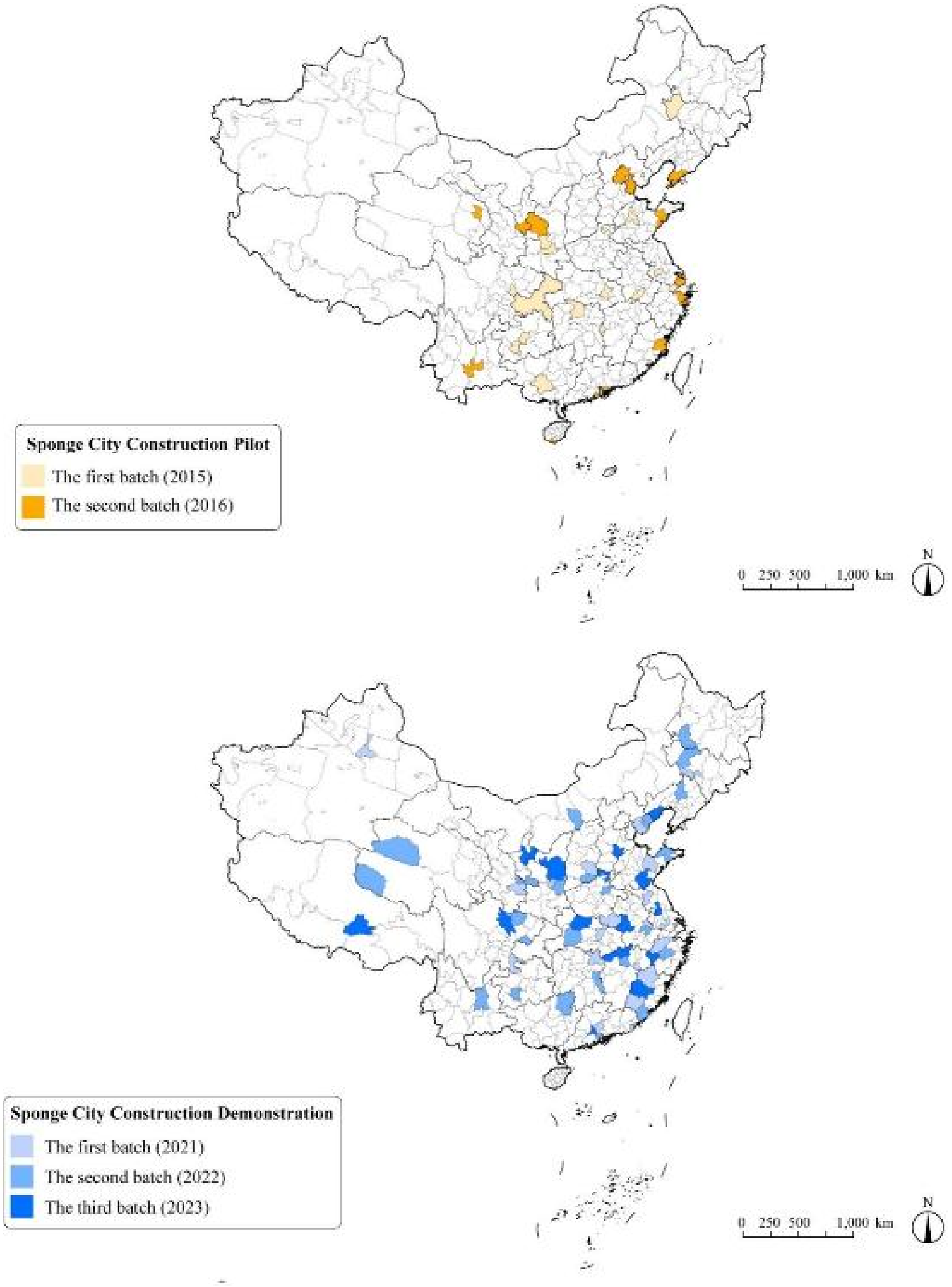

Figure C4. The distribution of "Sponge Cities Pilot Program" and "Sponge City Demonstration Cities

Notable Example of Sponge City Initiatives

Shanghai's sponge city initiative is transforming the urban landscape into a sustainable and resilient

environment by integrating natural and engineered water management systems. The city employs several key strategies to manage rainwater and reduce flooding [10].

- **Permeable pavements and green roofs,** as seen in places like Wujiaochang Sunken Plaza and Lin'gang Pocket Park, reduce surface runoff by allowing rainwater to infiltrate the ground and be absorbed by vegetation, thereby easing the burden on the drainage system.

- **Rain gardens** and artificial wetlands in areas like the Suzhou River Walkway and Yunjin Road Track Park filter pollutants and improve water quality. **Ecological ditches** and **retention areas**, such as those in Jinshan New City Public Green Space, slow water flow and enhance infiltration, further mitigating flood risks.

- **Sunken green spaces and rainwater harvesting systems** at locations like Wujiaochang Sunken Plaza and Rainbow Bay Green Space capture and store rainwater, reducing flood risks and decreasing the demand on potable water supplies.

- **Ecological riverbank renovations** in areas like the Shanghai Expo Urban Best Practices Area stabilize banks and improve flood control, while **smart water management systems** in key locations optimize infrastructure performance by monitoring rainfall and water quality in real-time.

Through these integrated measures, Shanghai is not only addressing the challenges of urbanization but also setting a global benchmark for sustainable urban development.
The Sponge City initiative is a key part of China's long-term strategy to create more resilient and sustainable urban environments. The lessons learned from the pilot and demonstration cities are expected to inform national policies and encourage the adoption of sponge city principles across the country. As urbanization continues to accelerate, the Sponge City initiative represents an innovative approach to managing the environmental challenges posed by rapid urban growth and climate change.

C-5. Forest City, Park City, and Pocket Park Initiatives in China

China has launched several urban greening initiatives aimed at enhancing the quality of life in its cities, promoting environmental sustainability, and improving public health. Among these initiatives, "Forest City", "Park City", and "Pocket Park" programs are particularly significant, each with its own focus and objectives.

Forest City Initiative (森林城市)

The "Forest City" initiative is part of China's broader efforts to combat air pollution, improve biodiversity, and enhance urban livability through extensive afforestation. The initiative aims to increase the proportion of green spaces in cities by planting trees and creating urban forests. The concept goes beyond traditional urban parks by integrating forests into the very fabric of the city, making green spaces a fundamental part of urban infrastructure.

The key objectives of the initiative include increasing urban green coverage, improving air quality,

and promoting biodiversity. The goal is to achieve significant green coverage within urban areas by integrating trees and vegetation into streets, residential zones, and public spaces. This effort not only enhances the visual appeal of cities but also plays a crucial role in improving air quality. Urban forests help absorb pollutants, reduce the urban heat island effect, and contribute to a healthier atmosphere. Additionally, these urban forests provide habitats for various species, thereby promoting biodiversity and contributing to more resilient ecosystems within cities.

Notable examples of the Forest City initiative can be seen in cities like Shenzhen and Guangzhou. Shenzhen has become one of China's leading Forest Cities, thanks to extensive tree-planting campaigns that have transformed the city into a green oasis. Similarly, Guangzhou has implemented large-scale afforestation projects, integrating forests into urban planning and ensuring that green spaces are accessible to all residents. These efforts demonstrate the potential of urban forests to create healthier, more sustainable cities.

Park City Initiative (公园城市)

The "Park City" initiative represents a vision of urban development where parks and green spaces are central to city planning. This initiative emphasizes creating a harmonious relationship between urban life and nature, ensuring that every citizen has easy access to recreational green spaces.

The key objectives of the Park City initiative are to integrate parks into urban life, enhance urban aesthetics, and support sustainable development. Parks are designed to be within easy reach of all residents, providing spaces for recreation, social interaction, and relaxation. The initiative aims to beautify cities with well-designed parks, gardens, and public spaces, contributing to a more pleasant urban environment. Additionally, by incorporating green infrastructure, Park Cities seek to support sustainable urban growth, reduce pollution, and enhance residents' overall well-being.

Notable examples of the Park City initiative include Chengdu and Shanghai. Chengdu is pioneering the Park City concept with large-scale projects that integrate green spaces throughout the city. The Tianfu Greenway, for example, is a vast network of parks and green corridors connecting various parts of the city. Similarly, Shanghai has been developing numerous urban parks and green spaces, seamlessly integrating them into the city’s dense urban fabric to create a more livable environment.

Pocket Park Initiative (口袋公园)

The "Pocket Park" initiative is a grassroots approach to urban greening that focuses on creating small, accessible green spaces scattered throughout the city. Pocket Parks are typically small parcels of land, often repurposed from underused or vacant lots, and are transformed into green spaces for community use.

The key objectives of the Pocket Park initiative include maximizing green space in dense urban areas, enhancing community engagement, and promoting environmental awareness. Pocket Parks are designed to bring nature into the most densely populated urban areas, offering residents a nearby retreat from the hustle and bustle of city life. These parks are often developed with input from local communities, ensuring they meet the specific needs and desires of residents. Additionally, by making

green spaces more accessible, the initiative helps raise awareness about the importance of urban green spaces and encourages residents to participate in their maintenance and care.

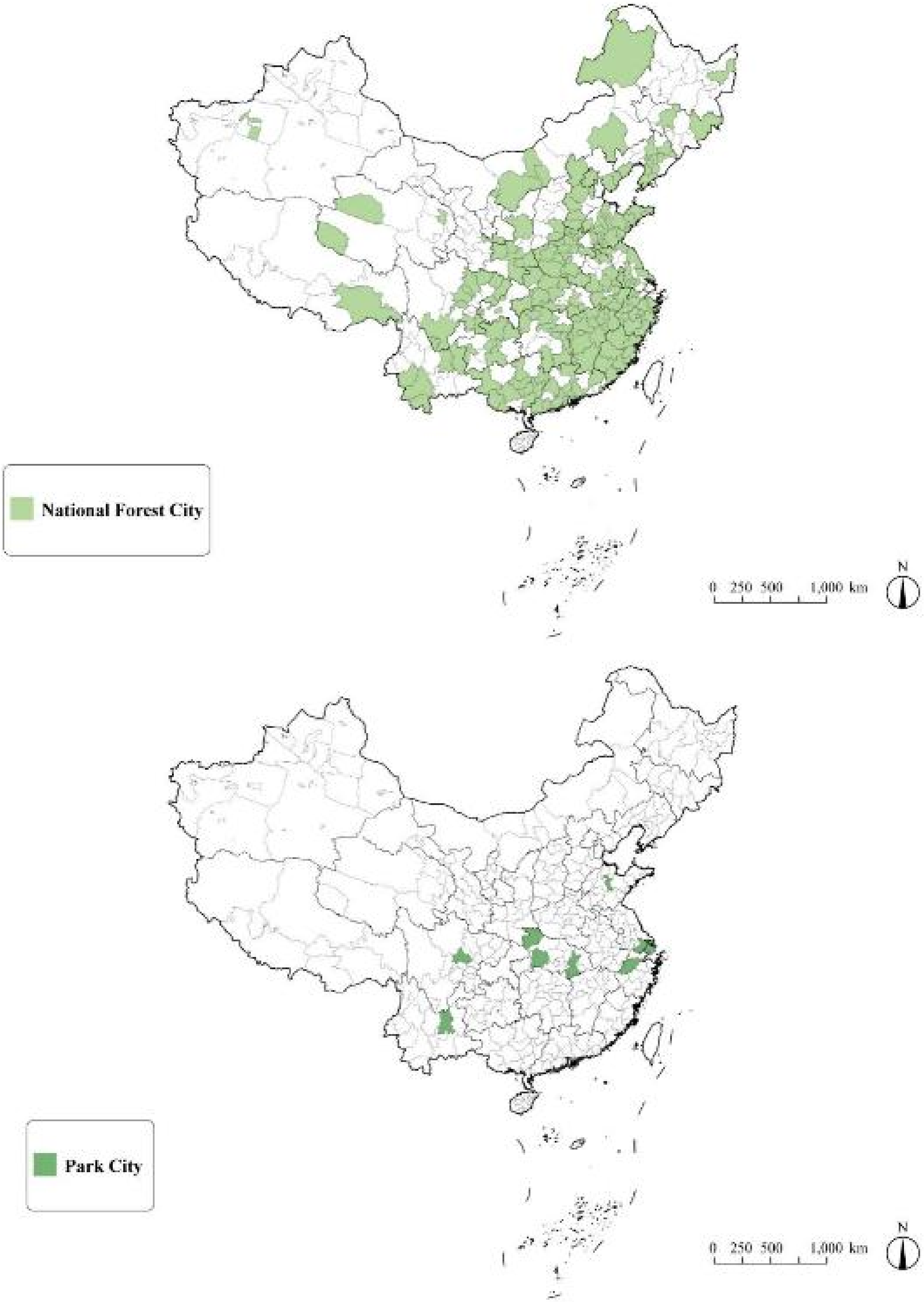

Figure C5. The distribution of "National Forest Cities" and "Park Cities"

Notable examples of the Pocket Park initiative include Beijing and Nanjing. Beijing has implemented numerous Pocket Parks across the city, particularly in older neighborhoods where larger parks are scarce. These small green spaces provide residents with convenient spots for

relaxation and socialization. Similarly, Nanjing has creatively utilized small plots of land to develop Pocket Parks, enhancing the urban landscape and providing green spaces in densely built-up areas.

China's "Forest City", "Park City", and "Pocket Park" initiatives are integral to the country's efforts to create more sustainable, healthy, and livable urban environments. By integrating nature into urban planning at various scales—from large urban forests to small pocket parks—these initiatives contribute to improved air quality, enhanced biodiversity, and greater public well-being. They also reflect China's commitment to sustainable urban development and its recognition of the vital role that green spaces play in the quality of urban life.

C-6. China's Three-North Shelter Forest Program (The Green Great Wall)

The Three-North Shelter Forest Program (三北防护林工程), also known as the "Green Great Wall" (绿色长城), is one of the most ambitious and extensive reforestation projects in the world. Launched in 1978, this program aims to combat desertification, soil erosion, and environmental degradation in the Three-North region of China, which includes the northeastern (东北), northwestern (西北), and northern (华北) parts of the country. It was established to achieve the following objectives:

- **Combating Desertification:** The program seeks to halt and reverse the spread of deserts, particularly the Gobi Desert, which has been encroaching on agricultural land and urban areas.

- **Preventing Soil Erosion:** By planting trees and shrubs, the program aims to stabilize the soil, reduce erosion, and protect agricultural land from being lost to desertification.

- **Improving the Environment:** The project aims to improve the overall environment of the Three-North region by increasing forest coverage, reducing dust storms, and enhancing local climate conditions.

- **Promoting Sustainable Development:** The program is designed to support sustainable agricultural and economic development by protecting land resources and improving living conditions for local populations.

The Three-North Shelter Forest Program covers a vast area of approximately 4.07 million square kilometers, which is about 42% of China's total land area. The project is planned to be completed in several phases over a span of 70 years, from 1978 to 2050. By the end of the project, the goal is to increase forest coverage in the region from 5% to over 15%. The program involves the planting of billions of trees across arid and semi-arid areas, creating shelterbelts that protect against winds, stabilize sand dunes, and help retain moisture in the soil. As of recent years, the Three-North Shelter Forest Program has made significant progress:

- **Increased Forest Coverage:** Forest coverage in the Three-North region has increased substantially, helping to slow the advance of deserts and rehabilitate degraded land.

- **Reduction in Dust Storms:** The frequency and severity of dust storms in northern China have decreased, contributing to better air quality in the region.

- **Improved Agricultural Productivity:** The stabilization of soil and improvement in local climate conditions have had positive effects on agricultural productivity, benefiting local communities.

- **Biodiversity Conservation:** The program has also contributed to the conservation of biodiversity by creating new habitats for various plant and animal species.

Despite its successes, the Three-North Shelter Forest Program has faced several challenges. The Survival Rates of Trees need to be concerned. In some areas, harsh climatic conditions and poor soil quality have led to low survival rates for planted trees. Besides, it has sometimes strained local water resources, as large-scale tree planting requires significant amounts of water, which can be scarce in arid regions. Also, ensuring the long-term sustainability of the project remains a challenge, as ongoing maintenance and care of the planted forests are essential for their continued success.

The Three-North Shelter Forest Program remains a cornerstone of China's efforts to combat desertification and promote environmental sustainability. As the project moves towards its completion in 2050, continuous efforts are needed to address the challenges and ensure that the gains made are preserved and built upon. The program serves as a model for large-scale environmental restoration projects globally and reflects China's commitment to addressing ecological issues through innovative and large-scale initiatives.

D. Best practice and sub-national cases in China

D-1. City-specific model linking weather and health data for better preparation of health risks

In China, extreme weather events, particularly heatwaves and cold spells, are increasingly linked to various health risks, including heatstroke, respiratory diseases, and cardiovascular incidents. Two cities, Tianjin and Shanghai, have developed effective weather-health models to mitigate these risks, demonstrating significant advances in the integration of weather data into health risk management.

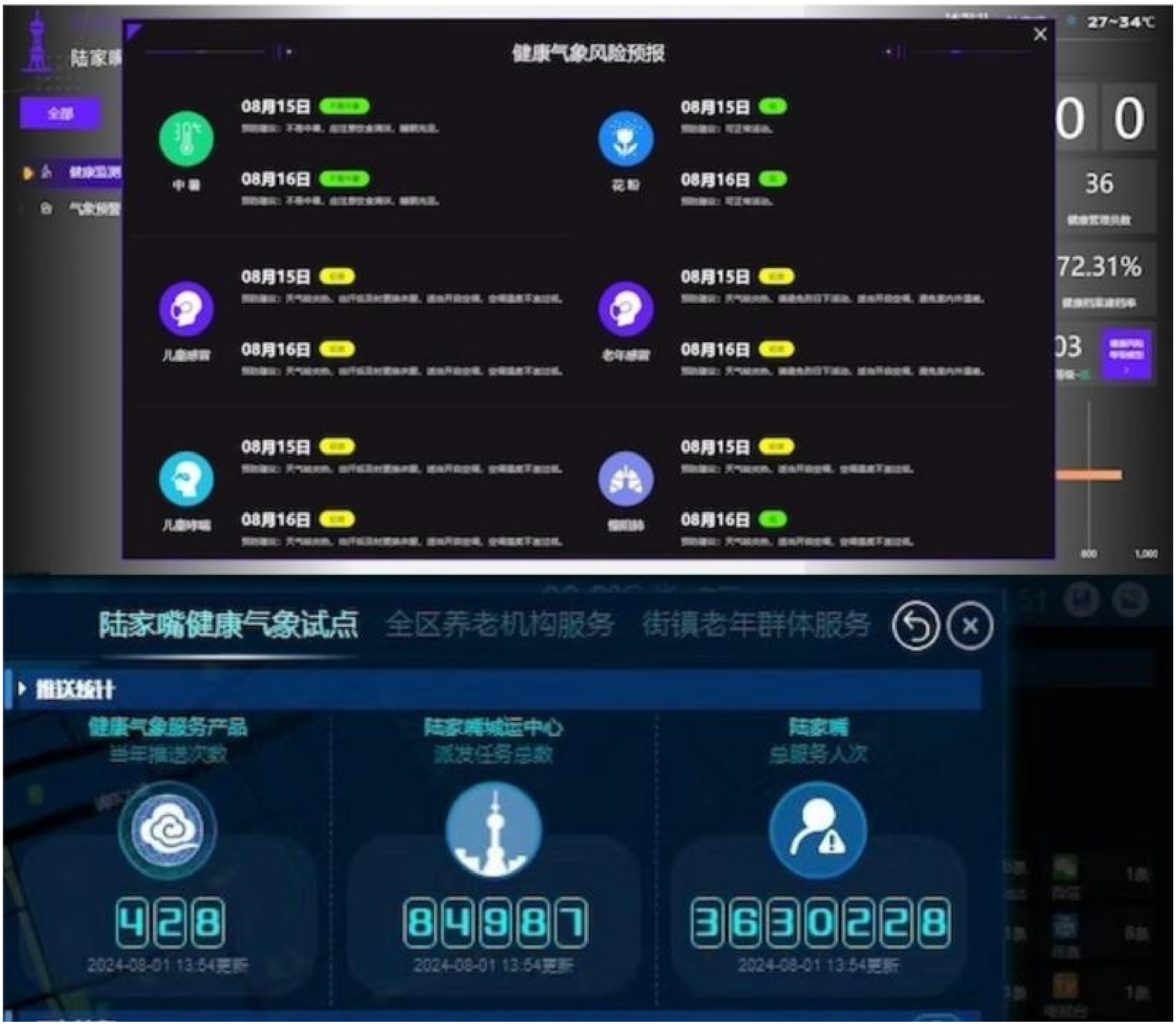

Figure D1. Lujiazui community, Shanghai is pilotly integrating health risk prediction into residents' health management

Tianjin has established a pioneering model focused on weather-induced stroke risks, particularly during extreme cold spells. The city has implemented a "Stroke Meteorological Risk Early Warning System" specifically targeting stroke, which is a leading cause of morbidity and mortality in China. Tianjin's system includes a three-tiered early warning structure (yellow, orange, red) that issues alerts based on factors such as low temperatures and abrupt temperature fluctuations. A key achievement in Tianjin's approach is the creation of the local standard, Stroke Meteorological Risk Early Warning Level Classification Standard (《脑卒中气象风险预警等级划分规范》), which sets clear criteria for issuing warnings. By integrating meteorological data with health statistics, Tianjin's model has demonstrated a reduction in stroke-related hospital admissions by up to 20.3% following early warnings, resulting in substantial cost savings and better public health outcomes.

Shanghai has also developed another comprehensive "weather-health risk prediction - urban climate resilience model", particularly focusing on chronic diseases such as cardiovascular conditions and respiratory diseases, which are exacerbated by extreme weather conditions. The model incorporates a

wide range of data, from meteorological forecasts to health statistics, to predict and issue warnings for diseases like heatstroke, asthma, and chronic obstructive pulmonary disease (COPD). Now Shanghai has successfully integrated these weather-health forecasts into its "smart city" framework (Figure 11). A key innovation is the digital health service mode that provides personalized health advice based on weather forecasts to individuals, particularly vulnerable groups like the elderly and chronic disease patients. In collaboration with local healthcare providers, Shanghai has launched services that enable health professionals to act on early warnings, thus preventing hospital admissions and reducing healthcare costs. For instance, health risk forecasting for COPD patients in the Pudong New Area has led to a 17.6% reduction in clinic visits and a 2.5% reduction in healthcare costs per patient.

D-2. 15-minute life circle in Meishan and Shanghai

The 15-minute life circle is an urban planning approach aimed at enhancing the quality of life by ensuring that residents can access essential services within a 15-minute walk or bike ride from their homes. This concept is designed to reduce reliance on cars, alleviate traffic congestion, and improve overall community well-being by making daily necessities, such as shopping, healthcare, education, and recreational facilities, readily accessible. The primary goal is to create more livable, sustainable, and resilient urban environments.

Meishan, a city in Sichuan province, is actively working to implement the 15-minutes life circle concept. In Jianggong community, each residential district has a "15-minute life circle" guide map, which delimits the 15-minute life circle with the current district as the center, and marks the basic service functions and public activity space required by the daily life of the residents, as well as the supporting residential functions such as commerce, culture, sports, health and education. The comprehensive cultural activity center in this community is equipped with a community library, a parent school, a juvenile activity room, and a science and technology experience hall, etc. Various activities are held regularly to meet the cultural and spiritual needs of the surrounding residents [11].

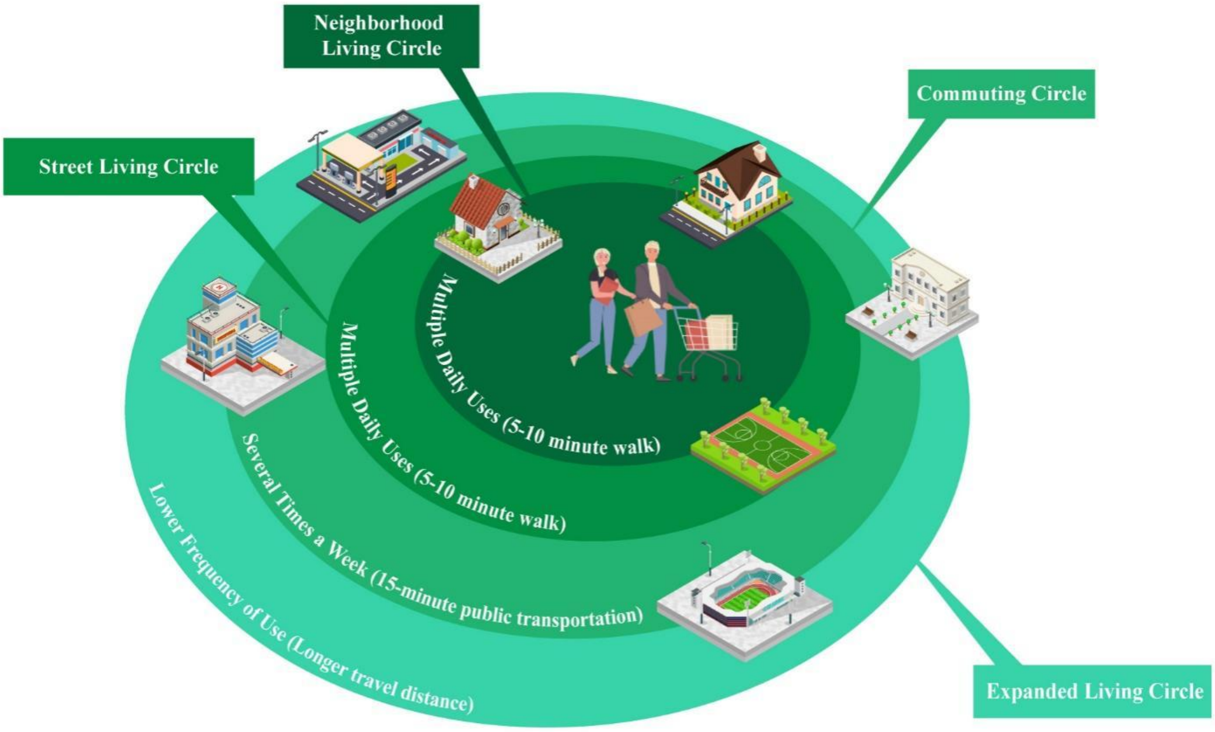

Figure D2. 15-minute life circle function schematic

Shanghai has actively advanced the 15-minutes life circle concept through a series of strategic actions. The city initiated its journey by developing and releasing the "***Planning Guidelines for 15-Minute Community Life Circle***" [12], and further solidified its commitment by launching the "15-minute Community Life Circle" Action Initiative in collaboration with 52 other cities during the Shanghai Urban Space Art Season in 2021. This initiative has established Shanghai as a leading example in national efforts to create 15-minute community life circles.

To implement this initiative effectively, Shanghai established a joint meeting system coordinated by the Shanghai Municipal Bureau of Planning and Natural Resources. In early 2023, the Shanghai "15-minute Community Life Circle" Action Conference Office released the "2023 Shanghai 15-minute Community Life Circle Action Plan" [13]. This plan outlines 1,600 designated circles within the city—860 in urban areas and 740 in rural areas—and incorporates various service categories such as convenience, community sports, and employment services.

By August 2023, around 1,300 out of 3,000 planned projects had been completed, with approximately 1,700 projects still under development. These projects span across education, culture, healthcare, elder care, sports, leisure, and employment, addressing critical community needs.

1+N Model in Shanghai

Shanghai has introduced the "1+N" model as part of its 15-minutes life circle strategy, which includes [14]:

- **Bai Le Fang (百乐坊):** This model represents "1" in the 1+N framework and refers to a one-stop

service center that consolidates multiple functions into a single location. The Bai Le Fang centers are categorized into three types based on their target audience and functional scope:

- **Ideal Type (全龄共享的理想型):** Typically ranging from 2,000 to 4,000 square meters, this type is designed for areas with ample space and is intended to serve all age groups. These centers are strategically located in areas with favorable spatial conditions and ample land, providing a comprehensive range of services to meet the diverse needs of residents. For instance, in Xuhui District, 29 "Living Boxes" have been established, featuring essential facilities such as health stations, community kitchens, and spaces for elderly care, catering to the varied needs of the local population.

- **Basic Type for Elder and Youth Services (服务老幼人群的基本型):** Generally ranging from 1,000 to 2,000 square meters, this type is designed for areas with limited space and high population density. These centers address the fundamental needs of elderly and younger residents, such as child care, elder care, and community activities. In high-density areas where space is constrained, these centers fill service gaps and provide essential functions. For example, the Xinhua Road Street in Changning District has repurposed community spaces into a comprehensive service center for elderly care, offering integrated services such as health care, education, and entertainment for the elderly.

- **Liu Yi Ting (六艺亭):** Representing "N" in the 1+N model, this refers to small-scale, multi-functional service facilities ranging from 5 to 500 square meters. These facilities are designed to be flexible and adaptable, enhancing public spaces and providing emergency support when needed. An example is the "Wangjiang Yi" service station along the Huangpu River, which offers amenities such as resting spaces, public restrooms, and drinking water.

The 15-minutes life circle concept is closely connected to the goals of creating age-friendly and sustainable cities. By ensuring that essential services are within a short distance, the concept improves accessibility for elderly residents, who may have mobility challenges and require easier access to daily necessities. This proximity reduces the need for long commutes, thereby supporting seniors in maintaining their independence and improving their quality of life. Additionally, the development of community service centers and activity spaces within the 15-minute radius promotes social interaction and reduces isolation among older adults, contributing to their overall well-being. The focus on reducing automobile dependency also aligns with sustainability goals by decreasing carbon emissions and supporting environmentally friendly transportation options. Overall, the 15-minutes life circle concept fosters a more inclusive and sustainable urban environment that benefits all residents, particularly the elderly.

D-3. Panan's practice on combining environmental meteorological resources, traditional Chinese medicine, wellness tourism into integrated medical service and elderly care.

The recent culmination of the National Meteorological Healthcare Volunteer Experience in Panan County, Zhejiang Province, marked a significant step forward in combining **environmental meteorological resources, traditional Chinese medicine (TCM), wellness tourism into**

integrated of medical service and elderly care in China. Guided by the Public Meteorological Service Center of the China Meteorological Administration and hosted by the Panan County Government, this initiative highlights an innovative "meteorology + TCM + wellness tourism" model in elderly care, designed to foster the development of meteorological healthcare and wellness industries [15].

- **Unique Meteorological and Environmental Conditions:** Panan County, with its distinctive mountainous terrain and elevation of approximately 500 meters, offers a microclimate that is notably cooler and less humid than its surrounding regions. This unique environment, enriched with abundant negative oxygen ions, creates an ideal setting for health and wellness activities. These favorable conditions set Panan apart from other cities and were a key factor in the success of the healthcare initiative.

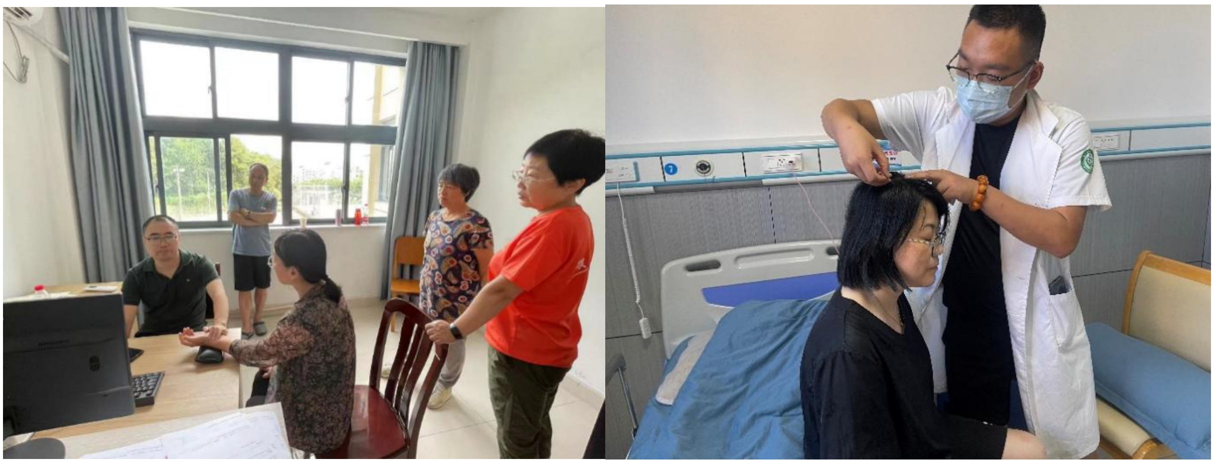

Figure D3. Elderly people are going to traditional Chinese medicine consultations to monitor their health on a daily basis

- **Daily Health Maintenance and TCM Integration:** The core of the program revolved around "health preservation according to weather conditions". Leveraging detailed meteorological forecasts provided by the Panan Meteorological Bureau, professional physicians developed personalized wellness plans for each participant. These plans included the integration of TCM into daily routines, such as incorporating medicinal herbs into meals, providing therapeutic herbal teas, and utilizing TCM diagnostics like tongue and pulse examination. Daily health monitoring was conducted, tracking physiological parameters such as blood pressure, pulse, temperature, and oxygen saturation, which were used to adjust and optimize the health regimens.

- **Enriching Elderly Life through Cultural and Physical Activities:** To further enhance the well-being of the elderly participants, the program included a variety of cultural and physical activities. Morning exercises featured traditional Chinese practices such as Tai Chi and Ba Duan Jin, guided by physicians to ensure the appropriate intensity for elderly individuals. Additionally, daily activities were organized to promote social interaction and mental stimulation, including board games, arts, and cultural performances. These activities were designed to enrich the lives of the elderly, providing them with a holistic approach to wellness that goes beyond physical health.

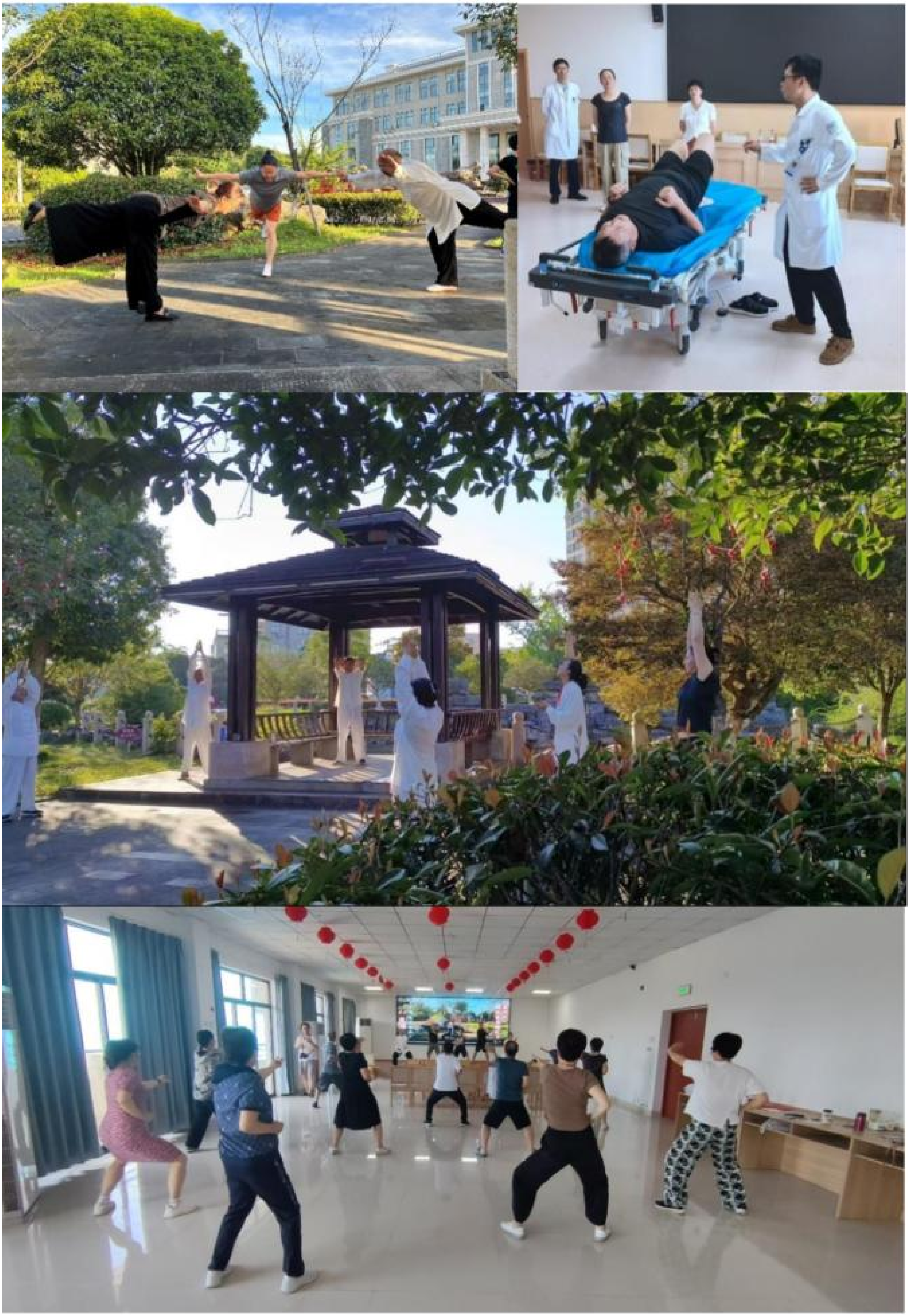

Figure D4. Elderly people are doing traditional physical exercises and sports rehabilitation such as Baduanjin and Tai Chi under the guidance of doctors and professionals

- **Knowledge Dissemination through Educational Seminars:** Educational seminars were a significant component of the program, focusing on health literacy and the relationship between weather and health. Topics covered included the proper use of medications, basic elderly health knowledge, and the impact of meteorological conditions on well-being. These sessions were aimed at empowering participants with the knowledge to manage their health more effectively and to understand the benefits of integrating meteorological insights into daily healthcare practices.

The initiative yielded promising results, with participants reporting improved physical and mental well-being. There were noticeable enhancements in blood pressure regulation, sleep quality, and overall vitality. These outcomes, while briefly noted, underscore the effectiveness of combining unique environmental conditions, TCM, and wellness practices to promote health and well-being among the elderly.

This innovative approach in Panan demonstrates the potential of integrating meteorological resources with TCM and wellness tourism to create a sustainable and effective model for elderly care, offering valuable insights for similar initiatives in other regions.

D-4. Dual-purpose infrastructure development in Beijing to enhance urban resilience to climate change

The construction of **dual-purpose infrastructure** [16] (“平急两用"公共基础设施建设) plays a critical role in enhancing urban resilience and preparedness by ensuring that public facilities can efficiently serve both everyday needs and emergency functions. This approach to infrastructure development integrates flexibility and adaptability into the design and operation of public services, enabling cities to respond swiftly and effectively to various unforeseen events such as natural disasters, public health emergencies, and other crises.

Beijing has been at the forefront of developing dual-purpose infrastructure, which is designed to serve both everyday needs and emergency situations. This approach is integral to enhancing urban resilience to climate change, ensuring that the city's infrastructure can adapt and respond to both routine and crisis conditions.

One of the key initiatives has been the transformation of public spaces and facilities into multifunctional hubs that can support disaster response efforts. For example, parks and open spaces have been equipped with emergency shelters and supplies, while also serving as recreational areas for residents during normal times. This dual functionality ensures that, in the event of extreme weather or other emergencies, these spaces can be quickly repurposed to accommodate displaced residents or emergency operations.

In addition to public spaces, Beijing has invested heavily in upgrading its transportation and communication networks. The city has integrated flood-resistant features into its subway systems, ensuring that they remain operational during heavy rainfall and flooding. Moreover, many of

Beijing's public buildings, such as schools and community centers, have been retrofitted with advanced HVAC systems that can be utilized to create climate-controlled environments for vulnerable populations during heatwaves or cold spells.

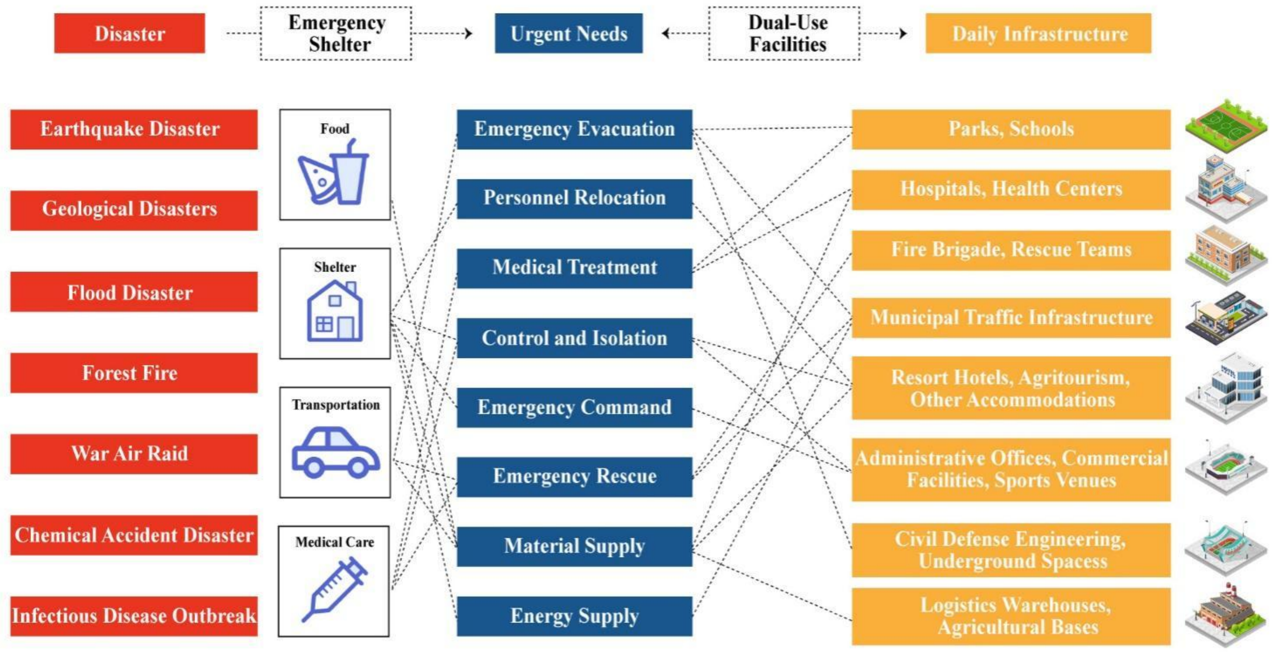

Figure D5. Application scenarios of dual-purpose infrastructures in Pinggu district, Beijing[34]

Beijing's commitment to dual-purpose infrastructure is further exemplified by its water management systems. The city has implemented an extensive network of stormwater retention and drainage facilities that not only reduce the risk of flooding but also help to conserve water during dry periods. These systems are designed to be flexible, allowing for quick adaptation to changing weather patterns and water availability, which is crucial in a city that faces both water scarcity and the threat of extreme weather events.

The city has also focused on ensuring that its energy infrastructure is resilient and adaptable. For instance, power plants and substations have been equipped with backup power supplies and are designed to withstand extreme weather conditions, minimizing the risk of power outages during emergencies. This is particularly important in a city as large and densely populated as Beijing, where even short-term disruptions can have significant consequences.

These efforts in Beijing serve as a model for other cities looking to enhance their urban resilience in the face of climate change. By integrating dual-purpose infrastructure into its urban planning, Beijing is not only safeguarding its residents against the impacts of climate change but also creating a more sustainable and livable city for the future.

Table S1. Characteristics of the included policy documents.

Number	Title in Chinese	Title in English	Department in Chinese	Department in English	Date of Issue	Domain
1	关于印发《智慧健康养老产业发展行动计划（2021-2025 年）》的通知[17]	Notice on Issuing the Action Plan for the Development of Smart Health and Elderly Care Industry (2021-2025)	工业和信息化部；民政部；国家卫生健康委	MIIT; MCA; NHC	2021/10/25	Age-friendly
2	关于进一步抓好互联网应用适老化及无障碍改造专项行动实施工作的通知[18]	Notice on Further Implementing the Special Action for the Adaptation and Barrier-Free Transformation of Internet Applications for the Elderly	工业和信息化部办公厅	General Office of MIIT	2021/4/12	Age-friendly
3	关于切实解决老年人运用智能技术困难便利老年人使用智能化产品和服务的通知[19]	Notice on Effectively Solving the Difficulties of the Elderly in Using Smart Technologies and Facilitating Their Use of Smart Products and Services	工业和信息化部	MIIT	2021/2/10	Age-friendly
4	关于印发《促进数字技术适老化高质量发展工作方案》的通知[20]	Notice on Issuing the Work Plan for Promoting High-Quality Development of Digital Technology Adaptation for the Elderly	工业和信息化部	MIIT	2023/12/27	Age-friendly
5	关于印发《互联网应用适老化及无障碍改造专项行动方案》的通知[21]	Notice on Issuing the Special Action Plan for the Adaptation and Barrier-Free Transformation of Internet Applications for the Elderly	工业和信息化部	MIIT	2020/12/25	Age-friendly
6	关于加强老年人居家医疗服务工作的通知[22]	Notice on Strengthening Home Medical Services for the Elderly	国家卫生健康委办公厅；国家中医药管理局办公室	General Office of NHC; Office of NATCM	2020/12/29	Age-friendly
7	关于建立积极应对人口老龄化重点联系城市机制的通知[23]	Notice on Establishing a Mechanism for Key Contact Cities to Actively Respond to Population Aging	国家发展改革委办公厅；民政部办公厅；国家卫生健康委办公厅	General Office of NDRC; General Office of MCA; General Office of NHC	2021/1/14	Age-friendly
8	关于进一步加强对口协同做好养老机构和社会福利机构老年人医疗服务工作的通知[24]	Notice on Further Strengthening Collaborative Medical Services for the Elderly in Elderly Care and Social Welfare Institutions	国务院联防联控机制医疗救治组	Medical Treatment Group of the State Council Joint Prevention and Control Mechanism	2023/1/12	Age-friendly

9	关于开展倡导文明健康绿色环保生活方式活动的意见 [25]	Opinions on Carrying out Activities to Promote Civilized, Healthy, Green, and Environmentally Friendly Lifestyles	全国爱国卫生运动委员会；中央精神文明建设指导委员会；健康中国行动推进委员会	NPHCC; CGCBS; HCAPC	2021/1/22	Environmentally sustainable
10	关于开展建设老年友善医疗机构工作的通知 [26]	Notice on Carrying out the Work of Building Elderly-Friendly Medical Institutions	国家卫生健康委；国家中医药管理局	NHC; NATCM	2020/12/2	Age-friendly
11	关于开展社区医养结合能力提升行动的通知 [27]	Notice on Carrying out the Action to Improve the Capacity of Community Medical and Elderly Care Integration	国家卫生健康委；国家发展改革委；民政部；财政部；住房城乡建设部；应急部；国家医保局；国家中医药局；中国残联	NHC; NDRC; MCA; MOF; MOHURD; MEM; NHSA; NATCM; CDPF	2022/3/25	Age-friendly
12	关于开展系统化全域推进海绵城市建设示范工作的通知 [28]	Notice on Carrying out the Demonstration Work of Promoting Sponge City Construction in a Systematic and Comprehensive Manner	财政部办公厅；住房城乡建设部办公厅；水利部办公厅	General Office of MOF; General Office of MOHURD; General Office of MWR	2021/4/25	Environmentally sustainable
13	关于全面加强老年健康服务工作的通知 [29]	Notice on Comprehensively Strengthening Elderly Health Services	国家卫生健康委；全国老龄办；国家中医药局	NHC; NWCA; NATCM	2022/1/17	Age-friendly
14	关于推进城市公共交通健康可持续发展的若干意见 [30]	Several Opinions on Promoting the Healthy and Sustainable Development of Urban Public Transportation	交通运输部；国家发展和改革委员会；公安部；财政部；人力资源和社会保障部；自然资源部；国家金融监督管理总局；中国证券监督管理委员会；中华全国总工会	MOT; NDRC; MPS; MOF; MOHRSS; MNR; NFRA; CSRC; ACFTU	2023/10/3	Environmentally sustainable

15	关于推进老年阅读工作的指导意见 [31]	Guiding Opinions on Promoting Elderly Reading Work	民政部；全国老龄办；中央宣传部；中央网信办；教育部；农业农村部；文化和旅游部；退役军人事务部；广电总局；全国总工会；共青团中央；全国妇联；中国残联；中国老龄协会	MCA; NWCA; CPCD; CAC; MOE; MARA; MCT; MVA; NRTA; ACFTU; CYLC; ACWF; CDPF; CAA	2024/10/28	Age-friendly
16	关于严禁养老机构违法违规开展医疗服务的通知 [32]	Notice on Strictly Prohibiting Elderly Care Institutions from Illegally Providing Medical Services	国家卫生健康委办公厅；民政部办公厅；国家中医药局综合司	General Office of NHC; General Office of MCA; Comprehensive Department of NATCM	2022/12/26	Age-friendly
17	关于印发“十四五”健康老龄化规划的通知 [33]	Notice on Issuing the "14th Five-Year Plan" for Healthy Aging	国家卫生健康委；教育部；科技部；工业和信息化部；财政部；人力资源社会保障部；住房和城乡建设部；退役军人事务部；市场监管总局；广电总局；体育总局；国家医保局；银保监会；国家中医药局；中国残疾人联合会	NHC; MOE; MOST; MIIT; MOF; MOHRSS; MOHURD; MVA; SAMR; NRTA; GAS; NHSA; CBIRC; NATCM; CDPF	2022/3/1	Age-friendly
18	关于印发《“十四五”环境健康工作规划》的通知 [34]	Notice on Issuing the "14th Five-Year Plan" for Environmental Health Work	生态环境部办公厅	General Office of MEE	2022/7/27	Environmentally sustainable
19	关于印发《“十四五”积极应对人口老龄化工程和托育建设实施方案》的通知 [35]	Notice on Issuing the Implementation Plan for the "14th Five-Year Plan" to Actively Respond to Population Aging and Childcare Construction	国家发展改革委；民政部；国家卫生健康委	NDRC; MCA; NHC	2021/6/17	Age-friendly
20	关于印发《2020 年新型城镇化建设和城乡融合发展重点任务》的通知 [36]	Notice on Issuing the Key Tasks for New Urbanization Construction and Urban-Rural Integration Development in 2020	国家发展改革委	NDRC	2020/4/9	Environmentally sustainable

21	关于印发《2021 年新型城镇化和城乡融合发展重点任务》的通知 [37]	Notice on Issuing the Key Tasks for New Urbanization and Urban-Rural Integration Development in 2021	国家发展改革委	NDRC	2021/4/13	Environmentally sustainable
22	关于印发《2022 年新型城镇化和城乡融合发展重点任务》的通知 [38]	Notice on Issuing the Key Tasks for New Urbanization and Urban-Rural Integration Development in 2022	国家发展改革委	NDRC	2022/3/17	Environmentally sustainable
23	关于印发《城市社区嵌入式服务设施建设导则（试行）》的通知 [39]	Notice on Issuing the Guidelines for the Construction of Embedded Service Facilities in Urban Communities (Trial)	国家发展改革委；住房城乡建设部；自然资源部	NDRC; MOHURD; MNR	2024/1/3	Age-friendly
24	关于印发《关于进一步促进养老服务消费提升老年人生活品质的若干措施》的通知 [40]	Notice on Issuing Several Measures to Further Promote Elderly Care Service Consumption and Improve the Quality of Life for the Elderly	民政部；商务部；中央网信办；国家发展改革委；工业和信息化部；公安部；财政部；人力资源社会保障部；自然资源部；住房城乡建设部；交通运输部；农业农村部；文化和旅游部；国家卫生健康委；中国人民银行；国务院国资委；市场监管总局；金融监管总局；广电总局；体育总局；国家医保局；国家邮政局；国家中医药局；国家消防救援局	MCA; MOFCOM; CAC; NDRC; MIIT; MPS; MOF; MOHRSS; MNR; MOHURD; MOT; MARA; MCT; NHC; PBC; SASAC; SAMR; NFRA; NRTA; GAS; NHSA; SPB; NATCM; NFRA	2024/10/31	Age-friendly

25	关于印发《国家适应气候变化战略 2035》的通知 [41]	Notice on Issuing the National Climate Change Adaptation Strategy 2035	生态环境部；国家发展和改革委员会；科学技术部；财政部；自然资源部；住房和城乡建设部；交通运输部；水利部；农业农村部；文化和旅游部；国家卫生健康委员会；应急管理部；中国人民银行；中国科学院；中国气象局；国家能源局；国家林业和草原局	MEE; NDRC; MOST; MOF; MNR; MOHURD; MOT; MWR; MARA; MCT; NHC; MEM; PBC; CAS; CMA; NEA; NFGA	2022/6/7	Environmentally sustainable
26	关于印发《环境基础设施建设水平提升行动（2023—2025 年）》的通知 [42]	Notice on Issuing the Action Plan for Improving the Level of Environmental Infrastructure Construction (2023-2025)	国家发展改革委；生态环境部；住房城乡建设部	NDRC; MEE; MOHURD	2023/7/25	Environmentally sustainable
27	关于印发《中央财政海绵城市建设示范补助资金绩效评价办法》的通知 [43]	Notice on Issuing the Performance Evaluation Measures for Central Financial Subsidy Funds for Sponge City Construction Demonstration	财政部办公厅；住房城乡建设部办公厅；水利部办公厅	General Office of MOF; General Office of MOHURD; General Office of MWR	2021/12/20	Environmentally sustainable
28	关于印发居家和社区医养结合服务指南（试行）的通知 [44]	Notice on Issuing the Guidelines for Home and Community Medical and Elderly Care Integration Services (Trial)	国家卫生健康委办公厅；国家中医药局综合司；国家疾控局综合司	General Office of NHC; Comprehensive Department of NATCM; Comprehensive Department of NCA	2023/11/8	Age-friendly
29	关于印发医疗卫生机构与养老服务机构签约合作服务指南（试行）的通知 [45]	Notice on Issuing the Guidelines for Contract Cooperation Services between Medical Institutions and Elderly Care Service Institutions (Trial)	国家卫生健康委办公厅；民政部办公厅；国家中医药管理局办公室	General Office of NHC; General Office of MCA; Office of NATCM	2020/12/18	Age-friendly
30	养老服务体系建设中央补助激励支持实施办法（2020 年修订版）[46]	Implementation Measures for Central Subsidy Incentive Support for the Construction of Elderly Care Service System (2020 Revision)	国家发展改革委；民政部；财政部	NDRC; MCA; MOF	2020/4/9	Age-friendly

31	关于做好积极应对人口老龄化重点联系城市有关工作的通知 [47]	Notice on Doing a Good Job in Key Contact Cities for Actively Responding to Population Aging	国家发展改革委	NDRC	2022/7/13	Age-friendly
32	关于加强城市内涝治理的实施意见 [48]	Implementation Opinions on Strengthening Urban Waterlogging Control	国家发展改革委	NDRC	2021/4/29	Environmentally sustainable
33	关于印发《"十四五"城镇生活垃圾分类和处理设施发展规划》的通知 [49]	Notice on Issuing the Development Plan for Urban Domestic Waste Classification and Treatment Facilities during the "14th Five-Year Plan" Period	国家发展改革委；住房城乡建设部	NDRC; MOHURD	2021/5/13	Environmentally sustainable
34	关于推介运用智能技术服务老年人示范案例的通知 [50]	Notice on Promoting Demonstration Cases of Using Smart Technologies to Serve the Elderly	国家发展改革委办公厅	General Office of NDRC	2021/9/24	Age-friendly
35	关于做好《国务院办公厅关于促进养老托育服务健康发展的意见》贯彻落实工作的通知 [51]	Notice on Implementing the Opinions of the General Office of the State Council on Promoting the Healthy Development of Elderly Care and Childcare Services	国家发展改革委办公厅	General Office of NDRC	2021/2/24	Age-friendly
36	印发《养老托育服务业纾困扶持若干政策措施》的通知 [52]	Notice on Issuing Several Policy Measures to Support the Elderly Care and Childcare Service Industry	国家发展改革委；教育部；科技部；民政部；财政部；人力资源社会保障部；住房城乡建设部；卫生健康委；人民银行；国务院国资委；税务总局；市场监管总局；银保监会	NDRC; MOE; MOST; MCA; MOF; MOHRSS; MOHURD; NHC; PBC; SASAC; STA; SAMR; CBIRC	2022/8/29	Age-friendly
37	关于印发《养老和家政服务标准化专项行动方案》的通知 [53]	Notice on Issuing the Special Action Plan for Standardization of Elderly Care and Home Services	国家标准化管理委员会；民政部；商务部	SAC; MCA; MOFCOM	2022/12/29	Age-friendly
38	关于促进医养结合服务高质量发展的指导意见 [54]	Guiding Opinions on Promoting the High-Quality Development of Medical and Elderly Care Integration Services	国家卫生健康委；民政部；国家医保局；国家中医药局；国家疾控局	NHC; MCA; NHSA; NATCM; NCA	2024/12/12	Age-friendly

39	关于推进健康乡村建设的指导意见 [55]	Guiding Opinions on Promoting Healthy Rural Construction	国家卫生健康委；全国爱卫办；国家发展改革委；民政部；财政部；生态环境部；住房城乡建设部；农业农村部；文化和旅游部；市场监管总局；体育总局；国家医保局；国家中医药局；国家疾控局	NHC; NPHCC; NDRC; MCA; MOF; MEE; MOHURD; MARA; MCT; SAMR; GAS; NHSA; NATCM; NCA	2024/8/1	Environmentally sustainable
40	关于开展失能老年人健康服务行动的通知 [56]	Notice on Carrying out the Action for Health Services for Disabled Elderly	国家卫生健康委办公厅；国家中医药管理局综合司	General Office of NHC; Comprehensive Department of NATCM	2024/8/16	Age-friendly
41	关于深化医疗卫生机构与养老机构协议合作的通知 [57]	Notice on Deepening Contract Cooperation between Medical Institutions and Elderly Care Institutions	国家卫生健康委办公厅；民政部办公厅；国家中医药局综合司；国家疾控局综合司	General Office of NHC; General Office of MCA; Comprehensive Department of NATCM; Comprehensive Department of NCA	2024/12/4	Age-friendly
42	关于开展老年痴呆防治促进行动(2023-2025年)的通知 [58]	Notice on Carrying out the Action to Promote the Prevention and Treatment of Alzheimer's Disease (2023-2025)	国家卫生健康委办公厅	General Office of NHC	2023/6/14	Age-friendly
43	关于开展老年听力健康促进行动（2024-2027年）的通知 [59]	Notice on Carrying out the Action to Promote Hearing Health for the Elderly (2024-2027)	国家卫生健康委办公厅	General Office of NHC	2024/6/25	Age-friendly
44	关于开展老年心理关爱行动的通知 [60]	Notice on Carrying out the Action for Psychological Care for the Elderly	国家卫生健康委办公厅	General Office of NHC	2022/6/20	Age-friendly
45	关于开展老年医疗护理服务试点工作的通知 [61]	Notice on Carrying out the Pilot Work of Elderly Medical Care Services	国家卫生健康委办公厅	General Office of NHC	2021/11/22	Age-friendly
46	关于实施进一步便利老年人就医举措的通知 [62]	Notice on Implementing Further Measures to Facilitate Medical Treatment for the Elderly	国家卫生健康委办公厅	General Office of NHC	2021/6/15	Age-friendly

47	关于探索开展抑郁症、老年痴呆防治特色服务工作的通知 [63]	Notice on Exploring Special Services for the Prevention and Treatment of Depression and Alzheimer's Disease	国家卫生健康委办公厅	General Office of NHC	2020/9/11	Age-friendly
48	关于提升老年医学医疗服务能力的通知 [64]	Notice on Improving the Medical Service Capacity of Geriatrics	国家卫生健康委办公厅	General Office of NHC	2024/11/4	Age-friendly
49	关于印发全国示范性老年友好型社区评分细则（试行）的通知 [2]	Notice on Issuing the Scoring Rules for National Demonstration Elderly-Friendly Communities (Trial)	国家卫生健康委办公厅	General Office of NHC	2021/6/2	Age-friendly
50	国家卫生健康委部署开展打击整治养老诈骗专项行动 [65]	National Health Commission Deploys Special Action to Combat and Rectify Elderly Fraud	国家卫生健康委老龄健康司	Department of Aging Health, NHC	2022/4/28	Age-friendly
51	关于做好方便老年人在基层医疗卫生机构看病就医有关工作的通知 [66]	Notice on Doing a Good Job in Facilitating Elderly People's Access to Medical Treatment in Primary Healthcare Institutions	国家卫生健康委基层司	Department of Primary Health, NHC	2021/1/8	Age-friendly
52	国家卫生健康委推进老年人失能（失智）预防干预试点 [67]	National Health Commission Promotes Pilot Projects for the Prevention and Intervention of Disability (Dementia) in the Elderly	国家卫生健康委老龄健康司	Department of Aging Health, NHC	2021/4/9	Age-friendly
53	关于发布药品说明书适老化及无障碍改革试点工作方案的公告 [68]	Announcement on Issuing the Pilot Work Plan for the Adaptation and Barrier-Free Reform of Drug Instructions for the Elderly	国家药监局	NMPA	2023/10/31	Age-friendly
54	关于进一步加强中医医院老年病科建设的通知 [69]	Notice on Further Strengthening the Construction of Geriatrics Departments in Traditional Chinese Medicine Hospitals	国家中医药管理局	NATCM	2023/12/15	Age-friendly
55	关于发展银发经济增进老年人福祉的意见 [70]	Opinions on Developing the Silver Economy to Enhance the Well-being of the Elderly	国务院办公厅	General Office of the State Council	2024/1/1	Age-friendly
56	《“十四五”城乡社区服务体系建设规划》[71]	"14th Five-Year Plan" for Urban and Rural Community Service System Construction	中华人民共和国民政部	MCA	2022/1/21	Environmentally sustainable
57	《关于促进养老托育服务健康发展的意见》[72]	Opinions on Promoting the Healthy Development of Elderly Care and Childcare Services	中华人民共和国人力资源和社会保障部	MOHRSS	2021/1/4	Age-friendly

58	《关于建立健全养老服务综合监管制度促进养老服务高质量发展的意见》[73]	Opinions on Establishing and Improving a Comprehensive Supervision System for Elderly Care Services to Promote High-Quality Development of Elderly Care Services	中华人民共和国人力资源和社会保障部	MOHRSS	2020/12/22	Age-friendly
59	关于切实解决老年人运用智能技术困难实施方案的通知[74]	Notice on the Implementation Plan to Effectively Solve the Difficulties of the Elderly in Using Smart Technologies	国务院办公厅	General Office of the State Council	2020/11/24	Age-friendly
60	关于加快建立健全绿色低碳循环发展经济体系的指导意见[75]	Guiding Opinions on Accelerating the Establishment and Improvement of a Green, Low-Carbon, and Circular Development Economic System	国务院	State Council	2021/2/2	Environmentally sustainable
61	关于印发“十四五”国家老龄事业发展和养老服务体系规划的通知[76]	Notice on Issuing the "14th Five-Year Plan" for National Aging Development and Elderly Care Service System	国务院	State Council	2022/2/21	Age-friendly
62	关于进一步加强适老化无障碍出行服务工作的通知[77]	Notice on Further Strengthening Barrier-Free Travel Services for the Elderly	交通运输部；国家铁路局；中国民用航空局；国家邮政局；中国残疾人联合会；全国老龄工作委员会办公室	MOT; NRA; CAAC; SPB; CDPF; NWCA	2024/1/12	Age-friendly
63	关于切实解决老年人运用智能技术困难便利老年人日常交通出行的通知[78]	Notice on Effectively Solving the Difficulties of the Elderly in Using Smart Technologies and Facilitating Their Daily Travel	交通运输部；人力资源和社会保障部；国家卫生健康委；中国人民银行；国家铁路局；中国民用航空局；中国国家铁路集团有限公司	MOT; MOHRSS; NHC; PBC; NRA; CAAC; CR	2020/12/28	Age-friendly
64	关于印发 2021 年便利老年人打车出行等 5 件更贴近民生实事工作方案的通知[79]	Notice on Issuing the Work Plan for 2021 to Facilitate Elderly People's Taxi Travel and Other 5 Closer-to-Life Practical Matters	交通运输部办公厅	General Office of MOT	2021/4/1	Age-friendly
65	关于印发 2022 年推行适老化交通出行服务等 5 件更贴近民生实事工作方案的通知[80]	Notice on Issuing the Work Plan for 2022 to Promote Elderly-Friendly Travel Services and Other 5 Closer-to-Life Practical Matters	交通运输部办公厅	General Office of MOT	2022/3/29	Age-friendly

66	关于印发 2023 年持续提升适老化无障碍交通出行服务等 5 件更贴近民生实事工作方案的通知 [81]	Notice on Issuing the Work Plan for 2023 to Continuously Improve Elderly-Friendly and Barrier-Free Travel Services and Other 5 Closer-to-Life Practical Matters	交通运输部办公厅	General Office of MOT	2023/4/11	Age-friendly
67	关于印发 2024 年适老化无障碍交通出行服务扩面提质增效等 5 件民生实事工作方案的通知 [82]	Notice on Issuing the Work Plan for 2024 to Expand, Improve, and Enhance Elderly-Friendly and Barrier-Free Travel Services and Other 5 Closer-to-Life Practical Matters	交通运输部办公厅	General Office of MOT	2024/3/1	Age-friendly
68	关于印发《科技支撑碳达峰碳中和实施方案（2022—2030 年）》的通知 [83]	Notice on Issuing the Implementation Plan for Science and Technology to Support Carbon Peaking and Carbon Neutrality (2022-2030)	科技部；国家发展改革委；工业和信息化部；生态环境部；住房城乡建设部；交通运输部；中科院；工程院；国家能源局	MOST; NDRC; MIIT; MEE; MOHURD; MOT; CAS; CAE; NEA	2022/6/24	Environmentally sustainable
69	老龄健康医养结合远程协同服务试点工作启动 [84]	Pilot Work on Remote Collaborative Services for Aging Health and Medical Care Integration Launched	国家卫生健康委老龄健康司	Department of Aging Health, NHC	2020/7/30	Age-friendly
70	关于组织开展中央财政支持经济困难失能老年人集中照护服务工作的通知 [85]	Notice on Organizing Central Financial Support for Centralized Care Services for Economically Disabled Elderly	民政部；财政部	MCA; MOF	2023/10/10	Age-friendly
71	关于加强养老机构非法集资防范化解工作的意见 [86]	Opinions on Strengthening the Prevention and Resolution of Illegal Fundraising in Elderly Care Institutions	民政部；公安部；市场监管总局；中国银保监会	MCA; MPS; SAMR; CBIRC	2022/11/7	Age-friendly
72	关于印发《积极发展老年助餐服务行动方案》的通知 [87]	Notice on Issuing the Action Plan for Actively Developing Elderly Meal Assistance Services	民政部；国家发展改革委；财政部；人力资源社会保障部；自然资源部；住房城乡建设部；农业农村部；商务部；应急管理部；税务总局；市场监管总局	MCA; NDRC; MOF; MOHRSS; MNR; MOHURD; MARA; MOFCOM; MEM; STA; SAMR	2023/11/14	Age-friendly

73	关于加快实施老年人居家适老化改造工程的指导意见 [88]	Guiding Opinions on Accelerating the Implementation of Home Adaptation Projects for the Elderly	民政部；国家发展改革委；财政部；住房和城乡建设部；国家卫生健康委；银保监会；国务院扶贫办；中国残联；全国老龄办	MCA; NDRC; MOF; MOHURD; NHC; CBIRC; LGOP; CDPF; NWCA	2020/7/10	Age-friendly
74	关于加强养老机构预收费监管的指导意见 [89]	Guiding Opinions on Strengthening the Supervision of Pre-Charges in Elderly Care Institutions	民政部；国家发展改革委；公安部；财政部；中国人民银行；市场监管总局；金融监管总局	MCA; NDRC; MPS; MOF; PBC; SAMR; NFRA	2024/4/23	Age-friendly
75	关于加强养老服务人才队伍建设的意见 [90]	Opinions on Strengthening the Construction of Elderly Care Service Talent Teams	民政部；国家发展改革委；教育部；财政部；人力资源社会保障部；住房城乡建设部；农业农村部；商务部； 国家卫生健康委；市场监管总局；税务总局；全国老龄办	MCA; NDRC; MOE; MOF; MOHRSS; MOHURD; MARA; MOFCOM; NHC; SAMR; STA; NWCA	2023/12/31	Age-friendly
76	关于组织开展基本养老服务综合平台试点的通知 [91]	Notice on Organizing Pilot Projects for the Comprehensive Platform for Basic Elderly Care Services	民政部；国家数据局	MCA; NDA	2024/1/23	Age-friendly
77	关于强化养老服务领域食品安全管理的意见 [92]	Opinions on Strengthening Food Safety Management in Elderly Care Services	民政部；市场监管总局	MCA; SAMR	2021/9/13	Age-friendly
78	《关于发挥基层群众性自治组织作用加强城乡社区防汛救灾工作的指导意见》[93]	Guiding Opinions on Giving Play to the Role of Grassroots Mass Autonomous Organizations to Strengthen Flood Control and Disaster Relief in Urban and Rural Communities	中华人民共和国民政部	MCA	2020/8/4	Environmentally sustainable

79	关于加快发展农村养老服务的指导意见[94]	Guiding Opinions on Accelerating the Development of Rural Elderly Care Services	民政部；中央精神文明建设办公室；农业农村部；国家发展改革委；教育部；司法部；财政部；人力资源社会保障部；自然资源部；住房城乡建设部；国家卫生健康委；应急管理部；中国人民银行；市场监管总局；金融监管总局；国务院国资委；国家医保局；国家邮政局；国家消防救援局；全国供销合作总社；中国残联；全国老龄办	MCA; CCO; MARA; NDRC; MOE; MOJ; MOF; MOHRSS; MNR; MOHURD; NHC; MEM; PBC; SAMR; NFRA; SASAC; NHSA; SPB; NFRA; ACFSC; CDPF; NWCA	2024/5/8	Age-friendly
80	关于开展特殊困难老年人探访关爱服务的指导意见[95]	Guiding Opinions on Carrying out Visiting and Caring Services for Special Difficult Elderly	民政部；中央政法委；中央文明办；教育部；财政部；住房城乡建设部；农业农村部；卫生健康委；中国残联；全国老龄办	MCA; CPLC; CCO; MOE; MOF; MOHURD; MARA; NHC; CDPF; NWCA	2022/9/27	Age-friendly
81	《关于深入推进智慧社区建设的意见》[96]	Opinions on Deepening the Construction of Smart Communities	民政部；中央政法委；中央网信办；发展改革委；工业和信息化部；公安部；财政部；住房城乡建设部；农业农村部	MCA; CPLC; CAC; NDRC; MIIT; MPS; MOF; MOHURD; MARA	2022/5/10	Environmentally sustainable
82	关于落实《关于切实解决老年人运用智能技术困难的实施方案》的通知[97]	Notice on Implementing the Implementation Plan to Effectively Solve the Difficulties of the Elderly in Using Smart Technologies	民政部办公厅	General Office of MCA	2020/12/29	Age-friendly

83	民政部部署建立全国统一养老机构等级评定体系 [98]	Ministry of Civil Affairs Deploys the Establishment of a National Unified Elderly Care Institution Rating System	中华人民共和国民政部	MCA	2020/1/2	Age-friendly
84	《关于推进"十四五"特殊困难老年人家庭适老化改造工作的通知》[99]	Notice on Promoting the Home Adaptation for Special Difficult Elderly Families during the "14th Five-Year Plan" Period	民政部；财政部；住房和城乡建设部；中国残联	MCA; MOF; MOHURD; CDPF	2022/2/24	Age-friendly
85	关于抓好大检查发现问题整改扎实推进农村人居环境整治的通知 [100]	Notice on Grasping the Rectification of Problems Found in the Major Inspection and Solidly Promoting the Improvement of Rural Living Environment	中华人民共和国农业农村部	MARA	2020/5/7	Environmentally sustainable
86	关于开展"智慧助老"行动的通知 [101]	Notice on Carrying out the "Smart Assistance for the Elderly" Action	全国老龄工作委员会办公室	NWCA	2020/12/1	Age-friendly
87	关于开展老年营养改善行动的通知 [102]	Notice on Carrying out the Action to Improve Nutrition for the Elderly	全国老龄办	NWCA	2022/10/20	Age-friendly
88	关于印发贯彻落实《中共中央 国务院关于加强新时代老龄工作的意见》任务分工方案的通知 [103]	Notice on Issuing the Task Division Plan for Implementing the Opinions of the Central Committee of the Communist Party of China and the State Council on Strengthening Aging Work in the New Era	全国老龄工作委员会	NWCA	2022/2/23	Age-friendly
89	《关于深入开展新时代"银龄行动"的指导意见》[104]	Guiding Opinions on Deepening the "Silver Age Action" in the New Era	中华人民共和国民政部	MCA	2024/9/23	Age-friendly
90	关于落实《关于切实解决老年人运用智能技术困难的实施方案》的通知 [105]	Notice on Implementing the Implementation Plan to Effectively Solve the Difficulties of the Elderly in Using Smart Technologies	文化和旅游部办公厅；国家文物局办公室	General Office of MCT; Office of NCHA	2020/12/22	Age-friendly
91	《中国公民生态环境与健康素养》[106]	Ecological Environment and Health Literacy of Chinese Citizens	生态环境部	MEE	2020/8/11	Environmentally sustainable
92	《关于统筹和加强应对气候变化与生态环境保护相关工作的指导意见》[107]	Guiding Opinions on Coordinating and Strengthening Climate Change Response and Ecological Environmental Protection	生态环境部	MEE	2021/1/13	Environmentally sustainable

93	关于印发强化农村防汛抗旱和供水保障专项推进方案的通知 [108]	Notice on Issuing the Special Promotion Plan for Strengthening Rural Flood Control, Drought Relief, and Water Supply Guarantee	水利部；国家发展改革委；财政部；生态环境部；住房城乡建设部；农业农村部；应急管理部；中国气象局；国家疾病预防控制局；国家乡村振兴局	MWR; NDRC; MOF; MEE; MOHURD; MARA; MEM; CMA; NCA; NRA	2022/9/28	Environmentally sustainable
94	关于进一步推进医养结合发展的指导意见 [109]	Guiding Opinions on Further Promoting the Development of Medical and Elderly Care Integration	卫生健康委；发展改革委；教育部；民政部；财政部；人力资源社会保障部；自然资源部；住房城乡建设部；应急部；市场监管总局；医保局	NHC; NDRC; MOE; MCA; MOF; MOHRSS; MNR; MOHURD; MEM; SAMR; NHSA	2022/7/18	Age-friendly
95	关于印发《国家气候变化健康适应行动方案（2024—2030 年）》的通知 [110]	Notice on Issuing the National Climate Change Health Adaptation Action Plan (2024-2030)	国家疾控局；国家发展改革委；财政部；生态环境部；住房城乡建设部；交通运输部；水利部；农业农村部；文化和旅游部；国家卫生健康委；应急管理部；中国气象局；国家林草局	NCA; NDRC; MOF; MEE; MOHURD; MOT; MWR; MARA; MCT; NHC; MEM; CMA; NFGA	2024/9/19	Environmentally sustainable
96	关于印发《乡镇（街道）突发事件应急预案编制参考》和《村（社区）突发事件应急预案编制参考》的通知 [111]	Notice on Issuing the Reference for Compiling Emergency Response Plans for Townships (Streets) and Villages (Communities)	应急管理部办公厅	General Office of MEM	2023/8/17	Environmentally sustainable
97	关于加强新时代老龄工作的意见 [112]	Opinions on Strengthening Aging Work in the New Era	国务院	State Council	2021/11/18	Age-friendly
98	关于完整准确全面贯彻新发展理念做好碳达峰碳中和工作的意见 [113]	Opinions on Fully, Accurately, and Comprehensively Implementing the New Development Concept to Do a Good Job in Carbon Peaking and Carbon Neutrality	中华人民共和国财政部	MOF	2021/11/23	Environmentally sustainable

99	关于推进新型城市基础设施建设打造韧性城市的意见 [114]	Opinions on Promoting New Urban Infrastructure Construction to Build Resilient Cities	中华人民共和国财政部	MOF	2024/12/6	Environmentally sustainable
100	《关于推动城乡建设绿色发展的意见》[115]	Opinions on Promoting Green Development in Urban and Rural Construction	中共中央办公厅;国务院办公厅	CCCPC; General Office of the State Council	2021/10/21	Environmentally sustainable
101	《关于推进基本养老服务体系建设的意见》[116]	Opinions on Promoting the Construction of Basic Elderly Care Service System	国务院	State Council	2023/5/21	Age-friendly
102	关于加快经济社会发展全面绿色转型的意见 [117]	Opinions on Accelerating the Comprehensive Green Transformation of Economic and Social Development	中华人民共和国财政部	MOF	2024/8/21	Environmentally sustainable
103	关于银行保险机构切实解决老年人运用智能技术困难的通知 [118]	Notice on Banking and Insurance Institutions Effectively Solving the Difficulties of the Elderly in Using Smart Technologies	中国银保监会办公厅	General Office of CBIRC	2021/3/26	Age-friendly
104	关于推动物业服务企业发展居家社区养老服务的意见 [119]	Opinions on Promoting Property Service Enterprises to Develop Home and Community Elderly Care Services	住房城乡建设部；发展改革委；民政部；卫生健康委；医保局；全国老龄办	MOHURD; NDRC; MCA; NHC; NHSA; NWCA	2020/11/24	Age-friendly
105	关于印发《口袋公园建设指南（试行）》的通知 [120]	Notice on Issuing the Guidelines for Pocket Park Construction (Trial)	住房城乡建设部办公厅	General Office of MOHURD	2024/6/17	Environmentally sustainable
106	关于印发海绵城市建设可复制政策机制清单的通知 [121]	Notice on Issuing the List of Replicable Policy Mechanisms for Sponge City Construction	住房城乡建设部办公厅	General Office of MOHURD	2024/5/2	Environmentally sustainable
107	关于全面开展城市体检工作指导意见 [122]	Guiding Opinions on Comprehensively Carrying out Urban Physical Examination Work	住房和城乡建设部	MOHURD	2023/12/6	Environmentally sustainable
108	关于印发“十四五”全国城市基础设施建设规划的通知 [123]	Notice on Issuing the "14th Five-Year Plan" National Urban Infrastructure Construction Plan	住房和城乡建设部；国家发展改革委	MOHURD; NDRC	2022/7/7	Environmentally sustainable
109	关于印发深入打好城市黑臭水体治理攻坚战实施方案的通知 [124]	Notice on Issuing the Implementation Plan for Deepening the Battle against Urban Black and Odorous Water Bodies	住房和城乡建设部；生态环境部；国家发展和改革委员会；水利部	MOHURD; MEE; NDRC; MWR	2022/7/17	Environmentally sustainable
110	关于进一步规范城市内涝防治信息发布等有关工作的通知 [125]	Notice on Further Regulating the Release of Urban Waterlogging Prevention Information and Related Work	住房和城乡建设部办公厅；国家发展改革委办公厅；中国气象局办公室	General Office of MOHURD; General Office of NDRC; Office of CMA	2022/6/28	Environmentally sustainable

111	关于做好 2023 年城市排水防涝工作的通知 [126]	Notice on Doing a Good Job in Urban Drainage and Waterlogging Prevention in 2023	住房和城乡建设部办公厅；国家发展改革委办公厅	General Office of MOHURD; General Office of NDRC	2023/4/16	Environmentally sustainable
112	关于做好因洪涝地质灾害影响贫困农户住房安全保障工作的通知 [127]	Notice on Doing a Good Job in Ensuring the Housing Safety of Poor Farmers Affected by Floods and Geological Disasters	住房和城乡建设部办公厅；国务院扶贫办综合司	General Office of MOHURD; Comprehensive Department of LGOP	2020/8/21	Environmentally sustainable
113	关于进一步明确海绵城市建设工作有关要求的通知 [128]	Notice on Further Clarifying the Requirements for Sponge City Construction Work	住房和城乡建设部办公厅	General Office of MOHURD	2022/4/18	Environmentally sustainable
114	关于开展城市公园绿地开放共享试点工作的通知 [129]	Notice on Carrying out Pilot Work on Open Sharing of Urban Park Green Spaces	住房和城乡建设部办公厅	General Office of MOHURD	2023/1/31	Environmentally sustainable
115	关于开展城市园林绿化垃圾处理和资源化利用试点工作的通知 [130]	Notice on Carrying out Pilot Work on Urban Landscaping Waste Treatment and Resource Utilization	住房和城乡建设部办公厅	General Office of MOHURD	2022/11/1	Environmentally sustainable
116	关于推动"口袋公园"建设的通知 [131]	Notice on Promoting the Construction of "Pocket Parks"	住房和城乡建设部办公厅	General Office of MOHURD	2022/7/29	Environmentally sustainable
117	关于印发完整居住社区建设指南的通知 [132]	Notice on Issuing the Guidelines for Complete Residential Community Construction	住房和城乡建设部办公厅	General Office of MOHURD	2021/12/17	Age-friendly
118	关于印发绿色社区创建行动方案的通知 [133]	Notice on Issuing the Action Plan for Green Community Creation	中华人民共和国住房和城乡建设部；中华人民共和国国家发展和改革委员会；中华人民共和国民政部；中华人民共和国公安部；中华人民共和国生态环境部；国家市场监督管理总局	MOHURD; NDRC; MCA; MPS; MEE; SAMR	2020/7/22	Environmentally sustainable
119	关于印发国家园林城市申报与评选管理办法的通知 [134]	Notice on Issuing the Management Measures for the Application and Selection of National Garden Cities	住房和城乡建设部	MOHURD	2022/1/6	Environmentally sustainable

120	住房和城乡建设部 国务院扶贫办联合下发通知要求 切实保障洪涝地质灾害受灾贫困农户住房安全 [135]	Ministry of Housing and Urban-Rural Development and State Council Leading Group Office of Poverty Alleviation and Development Jointly Issue Notice to Ensure the Housing Safety of Poor Farmers Affected by Floods and Geological Disasters	住房和城乡建设部	MOHURD	2020/8/24	Environmentally sustainable
121	关于印发《全面推进城市一刻钟便民生活圈建设三年行动计划(2023-2025)》的通知 [136]	Three-year Action Plan to Comprehensively promote the Construction of Urban one-quarter Hour Convenient Living Circle (2023-2025)	商务部办公厅；国家发展改革委办公厅；民政部办公厅；财政部办公厅；人力资源社会保障部办公厅；自然资源部办公厅；住房城乡建设部办公厅；文化和旅游部办公厅；国家卫生健康委办公厅；市场监管总局办公厅；金融监管总局办公厅；体育总局办公厅；国家邮政局办公室	MOFCOM; NDRC; MCA; MOF; MOHRSS; MNR; MOHURD; MCT; NHC; SAMR; NFRA; GAS; SPB	2023/7/11	Age-friendly
122	关于印发《深入实施以人为本的新型城镇化战略五年行动计划》的通知 [137]	The Five-Year Action Plan for the In-depth Implementation of the People-Centered New Urbanization Strategy	国务院	State Council	2024/7/28	Environmentally sustainable
123	关于印发“十四五”建筑节能与绿色建筑发展规划的通知 [138]	14th Five-Year Plan for Building Energy Efficiency and Green Building Development	住房和城乡建设部	MOHURD	2022/3/1	Environmentally sustainable
124	《关于积极稳步推进超大特大城市“平急两用”公共基础设施建设的指导意见》[139]	Guiding Opinions on Actively and Steadily Promoting the Construction of Public Infrastructure for Both Peacetime and Emergency Use in Megacities	国务院	State Council	2023/7/14	Environmentally sustainable

125	关于补齐公共卫生环境设施短板 开展城乡环境卫生清理整治的通知[140]	Notice on Addressing Shortcomings in Public Health Environmental Facilities and Carrying Out Urban and Rural Environmental Sanitation Cleanup and Improvement	国家发展改革委办公厅；生态环境部办公厅；住房城乡建设部办公厅；农业农村部办公厅；国家卫生健康委办公厅；国家疾控局综合司	NDRC; MEE; MOHURD; MARA; NHC; NCA	2023/6/30	Environmentally sustainable

Abbreviations: ACFSC, All-China Federation of Supply and Marketing Cooperatives; ACFTU, All-China Federation of Trade Unions; ACWF, All-China Women's Federation; CAA, China Aging Association; CAAC, Civil Aviation Administration of China; CAC, Cyberspace Administration of China; CAE, Chinese Academy of Engineering; CAS, Chinese Academy of Sciences; CBIRC, China Banking and Insurance Regulatory Commission; CCCPC, General Office of the Central Committee of the Communist Party of China; CCO, Central Civilization Office; CDPF, China Disabled Persons' Federation; CGCBS, Central Guidance Commission for Building Spiritual Civilization; CMA, China Meteorological Administration; CPCD, Central Propaganda Department; CPLC, Central Political and Legal Affairs Commission; CR, China State Railway Group Co., Ltd.; CSRC, China Securities Regulatory Commission; CYLC, Communist Youth League Central Committee; GAS, General Administration of Sport; HCAPC, Healthy China Action Promotion Committee; LGOP, State Council Leading Group Office of Poverty Alleviation and Development; MARA, Ministry of Agriculture and Rural Affairs; MCA, Ministry of Civil Affairs; MCT, Ministry of Culture and Tourism; MEE, Ministry of Ecology and Environment; MEM, Ministry of Emergency Management; MIIT, Ministry of Industry and Information Technology; MNR, Ministry of Natural Resources; MOE, Ministry of Education; MOF, Ministry of Finance; MOFCOM, Ministry of Commerce; MOHRSS, Ministry of Human Resources and Social Security; MOHURD, Ministry of Housing and Urban-Rural Development; MOJ, Ministry of Justice; MOST, Ministry of Science and Technology; MOT, Ministry of Transport; MPS, Ministry of Public Security; MVA, Ministry of Veterans Affairs; MWR, Ministry of Water Resources; NATCM, National Administration of Traditional Chinese Medicine; NCA, National Disease Control and Prevention Administration; NCHA, National Cultural Heritage Administration; NDA, National Data Administration; NDRC, National Development and Reform Commission; NEA, National Energy Administration; NFGA, National Forestry and Grassland Administration; NFRA, National Financial Regulatory Administration; NFRA, National Fire and Rescue Administration; NHC, National Health Commission; NHSA, National Healthcare Security Administration; NPHCC, National Patriotic Health Campaign Committee; NRA, National Railway Administration; NRTA, National Radio and Television Administration; NWCA, National Working Commission on Aging; PBC, People's Bank of China; SAC, Standardization Administration of China; SAMR, State Administration for Market Regulation; SASAC, State-owned Assets Supervision and Administration Commission; SPB, State Post Bureau; STA, State Taxation Administration.

Reference

1. NWCCA N. Notice on Setting up a National Model Age-Friendly Community [关于开展示范性全国老年友好型社区创建工作的通知]. 2020. https://www.gov.cn/zhengce/zhengceku/2020-12/14/content_5569385.htm. (accessed on 2025/01/05)
2. NHC GOo. Notice on Issuing the Scoring Rules for National Demonstration Elderly-Friendly Communities (Trial) [关于印发全国示范性老年友好型社区评分细则（试行）的通知]. 2021. http://www.nhc.gov.cn/lljks/zcwj2/202106/8e47c51f34d4444b9a1e583c6b7f4365.shtml. (accessed on 2025/01/07)
3. Commission FPH. Fujian Province 'National Demonstration elderly friendly community' creation case exhibition (21) : Xiamen Siming District Binhai Street Martial arts community [福建省“全国示范性老年友好型社区”创建案例展播(21)：厦门市思明区滨海街道演武社区]. 2021. https://wjw.fujian.gov.cn/jggk/csxx/lljkc/zhgl/202110/t20211022_5748191.htm.
4. MCA GOoMGOo. Notice on Carrying out the pilot Work of Building Complete Communities [关于开展完整社区建设试点工作的通知]. 2022. https://www.gov.cn/zhengce/zhengceku/2022-11/01/content_5723231.htm. (accessed on 2025/01/05)
5. The first 'complete community' in Hebei was opened in Handan [河北首个“完整社区”在邯郸启用]. 2020. https://www.thepaper.cn/newsDetail_forward_9109788.
6. MOHURD N. Notice on the Issuance of Pilot Work for the Construction of Climate-Resilient Cities [关于印发气候适应型城市建设试点工作的通知]. 2017. https://www.gov.cn/xinwen/2017-02/25/5170863/files/54dfbdaa54f44509a06689acd69f36fd.pdf. (accessed on 2025/01/04)
7. CDC MMMMMMC. Notice on Printing and Distributing the Pilot List for Deepening the Construction of Climate-Resilient Cities [关于印发深化气候适应型城市建设试点名单的通知]. 2024. https://www.gov.cn/zhengce/zhengceku/202405/P020240515620754729871.pdf. (accessed on 2025/01/04)
8. Council TS. Guiding Opinions on Promoting Sponge City Construction [关于推进海绵城市建设的指导意见]. 2015. https://www.gov.cn/zhengce/content/2015-10/16/content_10228.htm. (accessed on 2025/01/04)
9. MWR MM. Notice on Carrying out Systematic Demonstration Work on Promoting Sponge City Construction in the whole Area [关于开展系统化全域推进海绵城市建设示范工作的通知]. 2021. https://www.gov.cn/zhengce/zhengceku/2021-04/26/content_5602408.htm. (accessed on 2025/01/04)
10. Shanghai 15 sponge city construction and transformation cases share [上海市 15 个海绵城市建设改造案例分享]. 2018. https://www.sohu.com/a/247021442_649223.
11. Community 15-minute life circle, ‘circle’ out of a pleasant and beautiful life [社区 15 分钟生活圈 “圈”出惬意美好生活]. 2021. https://www.sohu.com/a/507751930_121106884.
12. Planning Guidelines for 15-Minute Community Life Circle [15 分钟社区生活圈规划导则]. 2016. https://up.caup.net/file/life-circle.pdf.
13. Resources SMBoPaN. 2023 Shanghai 15-minute Community Life Circle Action Plan [2023 年上海市“15 分钟社区生活圈”行动方案]. 2023. https://www.shanghai.gov.cn/gwk/search/content/c637171cef5a4a4baf2a3fc729604339.
14. Shanghai has comprehensively promoted the construction of '15-minute community life circle' this year - 1,600 'circles', bringing more 'fireworks' to the city. [上海今年全面推进“15 分钟社区生活圈”建设——1600 个“圈”，为城市带来更多“烟火气”]. 2023. https://www.shanghai.gov.cn/nw31406/20230920/e8dd636185994aafb4969925a0ee2c04.html.
15. The second National Meteorological medical care volunteer experience activity was successfully concluded [第二届全国气象医养志愿者体验活动圆满结束]. 2024. https://mp.weixin.qq.com/s/UqUXTV6RZpv9HPzb4ohCVw.
16. Key points and practical cases ofThe construction of dual-purpose infrastructure planning [“平急两用”公共基础

设施建设规划要点与实践案例]. 2024. https://mp.weixin.qq.com/s/GGbMk-jb5ZBjq09WUeBm8Q.

17. NHC MM. Notice on Issuing the Action Plan for the Development of Smart Health and Elderly Care Industry (2021-2025) [关于印发《智慧健康养老产业发展行动计划（2021-2025 年）》的通知]. 2021. http://www.nhc.gov.cn/lljks/zcwj2/202110/597c48d327744dc1976cf9b6972e5a4f/files/9129b14956d54d15bccbf1113b39c052.pdf. (accessed on 2025/01/08)

18. MIIT GOo. Notice on Further Implementing the Special Action for the Adaptation and Barrier-Free Transformation of Internet Applications for the Elderly [关于进一步抓好互联网应用适老化及无障碍改造专项行动实施工作的通知]. 2021. https://www.miit.gov.cn/jgsj/xgj/gzdt/art/2021/art_eddf498ded1b44829644bf20d28c9f6f.html. (accessed on 2024/12/27)

19. MIIT. Notice on Effectively Solving the Difficulties of the Elderly in Using Smart Technologies and Facilitating Their Use of Smart Products and Services [关于切实解决老年人运用智能技术困难便利老年人使用智能化产品和服务的通知]. 2021. https://www.miit.gov.cn/jgsj/xgj/wjfb/art/2021/art_f34ef0284e164abbb3c6ef193aa20586.html. (accessed on 2024/12/27)

20. MIIT. Notice on Issuing the Work Plan for Promoting High-Quality Development of Digital Technology Adaptation for the Elderly [关于印发《促进数字技术适老化高质量发展工作方案》的通知]. 2023. https://www.miit.gov.cn/zwgk/zcwj/wjfb/tz/art/2023/art_86781c41e1174d078af3ac43e90bd7ad.html. (accessed on 2024/12/27)

21. MIIT. Notice on Issuing the Special Action Plan for the Adaptation and Barrier-Free Transformation of Internet Applications for the Elderly [关于印发《互联网应用适老化及无障碍改造专项行动方案》的通知]. 2020. https://www.miit.gov.cn/api-gateway/jpaas-web-server/front/document/file-download?fileUrl=/cms_files/filemanager/1226211233/attach/202012/010f6dc6be574f7e9ed4230af23d5831.wps&fileName=%E4%BA%92%E8%81%94%E7%BD%91%E5%BA%94%E7%94%A8%E9%80%82%E8%80%81%E5%8C%96%E5%8F%8A%E6%97%A0%E9%9A%9C%E7%A2%8D%E6%94%B9%E9%80%A0%E4%B8%93%E9%A1%B9%E8%A1%8C%E5%8A%A8%E6%96%B9%E6%A1%88.wps. (accessed on 2024/12/27)

22. NATCM GOoNOo. Notice on Strengthening Home Medical Services for the Elderly [关于加强老年人居家医疗服务工作的通知]. 2020. http://www.nhc.gov.cn/yzygj/s7653pd/202012/19a2617ba8e641bea9ac2472ea04c82a.shtml. (accessed on 2025/01/07)

23. NHC GOoNGOoMGOo. Notice on Establishing a Mechanism for Key Contact Cities to Actively Respond to Population Aging [关于建立积极应对人口老龄化重点联系城市机制的通知]. 2021. https://www.gov.cn/zhengce/zhengceku/2021-02/03/content_5584551.htm. (accessed on 2024/12/25)

24. Administration DoM. Notice on Further Strengthening Collaborative Medical Services for the Elderly in Elderly Care and Social Welfare Institutions [关于进一步加强对口协同做好养老机构和社会福利机构老年人医疗服务工作的通知]. 2023. http://www.nhc.gov.cn/yzygj/s3594q/202301/9edf640fa90c4db99073bf509fcaef9f.shtml. (accessed on 2025/01/07)

25. HCAPC NC. Opinions on Carrying out Activities to Promote Civilized, Healthy, Green, and Environmentally Friendly Lifestyles [关于开展倡导文明健康绿色环保生活方式活动的意见]. 2021. http://www.nhc.gov.cn/guihuaxxs/gongwen1/202101/d723372da7aa4a42b3dce170721b4888.shtml. (accessed on 2025/01/09)

26. NATCM N. Notice on Carrying out the Work of Building Elderly-Friendly Medical Institutions [关于开展建设老年友善医疗机构工作的通知]. 2020. http://www.nhc.gov.cn/lljks/pqt/202012/03cdf0773f0c42fe86e577f2143f6721.shtml. (accessed on 2025/01/07)

27. CDPF NNMMMMNN. Notice on Carrying out the Action to Improve the Capacity of Community Medical and Elderly Care Integration [关于开展社区医养结合能力提升行动的通知]. 2022. http://www.nhc.gov.cn/lljks/zcwj2/202203/8157c1ba56ca41e08d0503e091a55198.shtml. (accessed on 2025/01/09)

28. MWR GOoMGOoMGOo. Notice on Carrying out the Demonstration Work of Promoting Sponge City Construction in a Systematic and Comprehensive Manner [关于开展系统化全域推进海绵城市建设示范工作的通知]. https://jjs.mof.gov.cn/zhengcefagui/202104/t20210425_3692009.htm. (accessed on 2025/01/05)

29. NATCM NN. Notice on Comprehensively Strengthening Elderly Health Services [关于全面加强老年健康服务工作的通知]. 2021. http://www.nhc.gov.cn/lljks/tggg/202201/e379815c740247d3be81d6b371cf6545.shtml. (accessed on 2025/01/07)

30. ACFTU MNMMMMNC. Several Opinions on Promoting the Healthy and Sustainable Development of Urban Public Transportation [关于推进城市公共交通健康可持续发展的若干意见]. 2023. https://www.gov.cn/zhengce/zhengceku/202310/content_6907977.htm. (accessed on 2025/01/06)

31. CAA MNCCMMMMNACAC. Guiding Opinions on Promoting Elderly Reading Work [关于推进老年阅读工作的指导意见]. 2024. https://www.gov.cn/zhengce/zhengceku/202411/content_6984995.htm. (accessed on 2024/12/25)

32. NATCM GOoNGOoMCDo. Notice on Strictly Prohibiting Elderly Care Institutions from Illegally Providing Medical Services [关于严禁养老机构违法违规开展医疗服务的通知]. 2022. https://www.gov.cn/zhengce/zhengceku/2022-12/30/content_5734233.htm. (accessed on 2024/12/25)

33. CDPF NMMMMMMMSNGNCN. Notice on Issuing the "14th Five-Year Plan" for Healthy Aging [关于印发"十四五"健康老龄化规划的通知]. 2022. https://view.officeapps.live.com/op/view.aspx?src=http%3A%2F%2Fwww.nhc.gov.cn%2Flljks%2Fpqt%2F202203%2Fc51403dce9f24f5882abe13962732919%2Ffiles%2Ff2b6cf2bed8d48d1876fc8c5139038a4.doc&wdOrigin=BROWSELINK. (accessed on 2025/01/08)

34. MEE GOo. Notice on Issuing the "14th Five-Year Plan" for Environmental Health Work [关于印发《"十四五"环境健康工作规划》的通知]. 2022. https://www.mee.gov.cn/xxgk2018/xxgk/xxgk05/202207/W020220729350033305073.pdf. (accessed on 2024/12/26)

35. NHC NM. Notice on Issuing the Implementation Plan for the "14th Five-Year Plan" to Actively Respond to Population Aging and Childcare Construction [关于印发《"十四五"积极应对人口老龄化工程和托育建设实施方案》的通知]. 2021. https://www.gov.cn/zhengce/zhengceku/2021-06/25/5620868/files/04b844773d1e4bb78dde9b8309aff945.pdf. (accessed on 2024/12/30)

36. NDRC. Notice on Issuing the Key Tasks for New Urbanization Construction and Urban-Rural Integration Development in 2020 [关于印发《2020年新型城镇化建设和城乡融合发展重点任务》的通知(发改规划〔2020〕532号)]. 2020. https://www.ndrc.gov.cn/xwdt/tzgg/202004/t20200409_1235945.html. (accessed on 2024/12/25)

37. NDRC. Notice on Issuing the Key Tasks for New Urbanization and Urban-Rural Integration Development in 2021 [关于印发《2021年新型城镇化和城乡融合发展重点任务》的通知]. 2021. https://www.ndrc.gov.cn/xwdt/tzgg/202104/t20210413_1272201.html. (accessed on 2024/12/25)

38. NDRC. Notice on Issuing the Key Tasks for New Urbanization and Urban-Rural Integration Development in 2022 [关于印发《2022年新型城镇化和城乡融合发展重点任务》的通知]. 2022. https://www.ndrc.gov.cn/xwdt/tzgg/202203/t20220317_1319456.html. (accessed on 2024/12/25)

39. MNR NM. Notice on Issuing the Guidelines for the Construction of Embedded Service Facilities in Urban Communities (Trial) [关于印发《城市社区嵌入式服务设施建设导则（试行）》的通知]. 2024. https://www.gov.cn/zhengce/zhengceku/202401/P020240117414409280245.pdf. (accessed on 2025/01/07)

40. NHSA; MMCNMMMMMMMMNPSSNNG. Notice on Issuing Several Measures to Further Promote Elderly Care Service Consumption and Improve the Quality of Life for the Elderly [关于印发《关于进一步促进养老服务消费提升老年人生活品质的若干措施》的通知]. 2024. https://www.gov.cn/zhengce/zhengceku/202411/content_6985707.htm. (accessed on 2024/12/30)

41. NFGA MNMMMMMMMMNMPCCN. Notice on Issuing the National Climate Change Adaptation Strategy 2035

[关于印发《国家适应气候变化战略 2035》的通知]. 2022. https://www.mee.gov.cn/xxgk2018/xxgk/xxgk03/202206/W020220613636562919192.pdf. (accessed on 2024/12/25)

42. MOHURD NM. Notice on Issuing the Action Plan for Improving the Level of Environmental Infrastructure Construction (2023-2025) [关于印发《环境基础设施建设水平提升行动（2023—2025 年）》的通知]. 2023. https://www.gov.cn/zhengce/zhengceku/202308/P020230824781190432393.pdf. (accessed on 2025/01/07)

43. MWR GOoMGOoMGOo. Notice on Issuing the Performance Evaluation Measures for Central Financial Subsidy Funds for Sponge City Construction Demonstration [关于印发《中央财政海绵城市建设示范补助资金绩效评价办法》的通知]. 2021. https://jjs.mof.gov.cn/tongzhigonggao/202201/P020220120381399019765.pdf. (accessed on 2025/01/05)

44. NCA GOoNCDoNCDo. Notice on Issuing the Guidelines for Home and Community Medical and Elderly Care Integration Services (Trial) [关于印发居家和社区医养结合服务指南（试行）的通知]. 2023. http://www.nhc.gov.cn/lljks/tggg/202311/6d6573c92b1a4c579383959354680458.shtml. (accessed on 2025/01/09)

45. NATCM GOoNGOoMOo. Notice on Issuing the Guidelines for Contract Cooperation Services between Medical Institutions and Elderly Care Service Institutions (Trial) [关于印发医疗卫生机构与养老服务机构签约合作服务指南（试行）的通知]. 2020. http://www.nhc.gov.cn/lljks/tggg/202012/4b2f6ed5d52d4e88a9ded73fe766df60.shtml. (accessed on 2025/01/08)

46. MOF NM. Implementation Measures for Central Subsidy Incentive Support for the Construction of Elderly Care Service System (2020 Revision) [《养老服务体系建设中央补助激励支持实施办法》（2020 年修订版）]. 2020. http://lgj.mofcom.gov.cn/zcgz/art/2020/art_ee8e1126070a4c1e9efc4337f385f029.html. (accessed on 2024/12/25)

47. NDRC. Notice on Doing a Good Job in Key Contact Cities for Actively Responding to Population Aging [关于做好积极应对人口老龄化重点联系城市有关工作的通知]. 2022. https://www.ndrc.gov.cn/fzggw/jgsj/shs/sjdt/202207/t20220713_1330437.html. (accessed on 2024/12/24)

48. NDRC. Implementation Opinions on Strengthening Urban Waterlogging Control [关于加强城市内涝治理的实施意见]. 2021. https://www.gov.cn/zhengce/content/2021-04/25/content_5601954.htm. (accessed on 2024/12/25)

49. MOHURD N. Notice on Issuing the Development Plan for Urban Domestic Waste Classification and Treatment Facilities during the "14th Five-Year Plan" Period [关于印发《“十四五”城镇生活垃圾分类和处理设施发展规划》的通知]. 2021. https://www.gov.cn/zhengce/zhengceku/2021-05/14/5606349/files/bd8ca92c767f4b8786b9374a76e0d1fd.pdf. (accessed on 2024/12/25)

50. NDRC GOo. Notice on Promoting Demonstration Cases of Using Smart Technologies to Serve the Elderly [关于推介运用智能技术服务老年人示范案例的通知]. 2021. https://www.gov.cn/zhengce/zhengceku/2021-09/29/5639975/files/bf03c72a9b014cfda5bfc5ec8deebcab.pdf. (accessed on 2024/12/25)

51. NDRC GOo. Notice on Implementing the Opinions of the General Office of the State Council on Promoting the Healthy Development of Elderly Care and Childcare Services [关于做好《国务院办公厅关于促进养老托育服务健康发展的意见》贯彻落实工作的通知]. 2021. https://www.gov.cn/zhengce/zhengceku/2021-03/10/content_5592022.htm. (accessed on 2025/01/05)

52. CBIRC NMMMMMMNPSSS. Notice on Issuing Several Policy Measures to Support the Elderly Care and Childcare Service Industry [印发《养老托育服务业纾困扶持若干政策措施》的通知]. 2022. https://jdjc.mof.gov.cn/fgzd/202209/t20220901_3837811.htm. (accessed on 2024/12/31)

53. MOFCOM SM. Notice on Issuing the Special Action Plan for Standardization of Elderly Care and Home Services [关于印发《养老和家政服务标准化专项行动方案》的通知]. 2022. https://view.officeapps.live.com/op/view.aspx?src=https%3A%2F%2Fwww.gov.cn%2Fzhengce%2Fzhengceku%2F2023-02%2F08%2F5740634%2Ffiles%2F2d17d32b2c694a08a6c4a293c4dc1402.docx&wdOrigin=BROWSELINK. (accessed on 2025/01/11)

54. NCA NMNN. Guiding Opinions on Promoting the High-Quality Development of Medical and Elderly Care Integration Services [关于促进医养结合服务高质量发展的指导意见]. 2024. https://www.gov.cn/zhengce/zhengceku/202412/content_6992253.htm. (accessed on 2025/01/06)

55. NCA NNNMMMMMMSGNN. Guiding Opinions on Promoting Healthy Rural Construction [关于推进健康乡村建设的指导意见]. 2024. https://www.gov.cn/gongbao/2024/issue_11666/202410/content_6983465.html. (accessed on 2025/01/06)

56. NATCM GOoNCDo. Notice on Carrying out the Action for Health Services for Disabled Elderly [关于开展失能老年人健康服务行动的通知]. 2024. http://www.nhc.gov.cn/lljks/s7785/202408/5ce825c6977c422ea9b4f8fba75fbe78.shtml. (accessed on 2025/01/07)

57. NCA GOoNGOoMCDoNCDo. Notice on Deepening Contract Cooperation between Medical Institutions and Elderly Care Institutions [关于深化医疗卫生机构与养老机构协议合作的通知]. 2024. http://www.nhc.gov.cn/lljks/tggg/202412/bf5e966dbe2f41bba43a89bf19d0af70.shtml. (accessed on 2025/01/08)

58. NHC GOo. Notice on Carrying out the Action to Promote the Prevention and Treatment of Alzheimer's Disease (2023-2025) [关于开展老年痴呆防治促进行动(2023-2025年)的通知]. 2023. http://www.nhc.gov.cn/lljks/tggg/202306/08c886def458469c8ff84e6dd6f2f7e0.shtml. (accessed on 2025/01/07)

59. NHC GOo. Notice on Carrying out the Action to Promote Hearing Health for the Elderly (2024-2027) [关于开展老年听力健康促进行动（2024-2027年）的通知]. 2024. http://www.nhc.gov.cn/lljks/tggg/202406/b37c0e2f2c3344f6bbeae260ee2930ce.shtml. (accessed on 2025/01/07)

60. NHC GOo. Notice on Carrying out the Action for Psychological Care for the Elderly [关于开展老年心理关爱行动的通知]. 2022. http://www.nhc.gov.cn/lljks/pqt/202206/c8cfbfd7dd464bc0afc6f55afe170bbb.shtml. (accessed on 2025/01/07)

61. NHC GOo. Notice on Carrying out the Pilot Work of Elderly Medical Care Services [关于开展老年医疗护理服务试点工作的通知]. 2021. http://www.nhc.gov.cn/yzygj/s7653pd/202111/5023db9c361b4e7f8c4bd6a735e6f4ac.shtml. (accessed on 2025/01/07)

62. NHC GOo. Notice on Implementing Further Measures to Facilitate Medical Treatment for the Elderly [关于实施进一步便利老年人就医举措的通知]. 2021. http://www.nhc.gov.cn/yzygj/s3594q/202106/f0378d1b6d4c48189f5457d8e3f902cc.shtml. (accessed on 2025/01/07)

63. NHC GOo. Notice on Exploring Special Services for the Prevention and Treatment of Depression and Alzheimer's Disease [关于探索开展抑郁症、老年痴呆防治特色服务工作的通知]. 2020. http://www.nhc.gov.cn/jkj/s7914/202009/a63d8f82eb53451f97217bef0962b98f.shtml. (accessed on 2025/01/07)

64. NHC GOo. Notice on Improving the Medical Service Capacity of Geriatrics [关于提升老年医学医疗服务能力的通知]. 2024. http://www.nhc.gov.cn/yzygj/s7655/202411/4b1ff98d22cb4135b621bdbb840e8a75.shtml. (accessed on 2025/01/07)

65. NHC. National Health Commission Deploys Special Action to Combat and Rectify Elderly Fraud [国家卫生健康委部署开展打击整治养老诈骗专项行动]. 2022. http://www.nhc.gov.cn/lljks/pqt/202204/27b63101912c4248a49366534ebf3b0f.shtml.

66. Department of Primary Health N. Notice on Doing a Good Job in Facilitating Elderly People's Access to Medical Treatment in Primary Healthcare Institutions [关于做好方便老年人在基层医疗卫生机构看病就医有关工作的通知]. 2021. http://www.nhc.gov.cn/jws/s7872/202101/2865bcef198c446fafc5a1a515bdea15.shtml. (accessed on 2025/01/07)

67. NHC. National Health Commission Promotes Pilot Projects for the Prevention and Intervention of Disability (Dementia) in the Elderly [国家卫生健康委推进老年人失能（失智）预防干预试点]. 2021. http://www.nhc.gov.cn/lljks/s7786/202104/c330604949a5447b9bf377b24537ed1c.shtml.

68. NMPA. Announcement on Issuing the Pilot Work Plan for the Adaptation and Barrier-Free Reform of Drug Instructions for the Elderly [关于发布药品说明书适老化及无障碍改革试点工作方案的公告]. 2023.

https://view.officeapps.live.com/op/view.aspx?src=https%3A%2F%2Fwww.gov.cn%2Fzhengce%2Fzhengceku%2F202310%2FP020231031721004335634.docx&wdOrigin=BROWSELINK. (accessed on 2025/01/06)

69. NATCM. Notice on Further Strengthening the Construction of Geriatrics Departments in Traditional Chinese Medicine Hospitals [关于进一步加强中医医院老年病科建设的通知]. 2023. https://www.gov.cn/zhengce/zhengceku/202312/content_6921300.htm. (accessed on 2024/12/25)

70. Council GOotS. Opinions on Developing the Silver Economy to Enhance the Well-being of the Elderly [关于发展银发经济增进老年人福祉的意见]. 2024. https://www.gov.cn/gongbao/2024/issue_11126/202401/content_6928803.html. (accessed on 2024/12/25)

71. MCA. "14th Five-Year Plan" for Urban and Rural Community Service System Construction [“十四五”城乡社区服务体系建设规划]. 2022. https://www.mca.gov.cn/gdnps/pc/content.jsp?id=116816&mtype=. (accessed on 2025/01/12)

72. NDRC GOo. Opinions on Promoting the Healthy Development of Elderly Care and Childcare Services [《关于促进养老托育服务健康发展的意见》]. 2020. https://www.gov.cn/zhengce/content/2020-12/31/content_5575804.htm. (accessed on 2024/12/25)

73. MOHRSS. Opinions on Establishing and Improving a Comprehensive Supervision System for Elderly Care Services to Promote High-Quality Development of Elderly Care Services [《关于建立健全养老服务综合监管制度促进养老服务高质量发展的意见》]. 2020. https://www.gov.cn/zhengce/content/2020-12/21/content_5571902.htm. (accessed on 2024/12/25)

74. Council GOotS. Notice on the Implementation Plan to Effectively Solve the Difficulties of the Elderly in Using Smart Technologies [关于切实解决老年人运用智能技术困难实施方案的通知]. 2020. http://www.mwr.gov.cn/zw/zgzygwywj/202011/t20201124_1478308.html. (accessed on 2024/12/25)

75. Council S. Guiding Opinions on Accelerating the Establishment and Improvement of a Green, Low-Carbon, and Circular Development Economic System [关于加快建立健全绿色低碳循环发展经济体系的指导意见]. 2021. http://bj.mof.gov.cn/ztdd/czysjg/zcfg/202104/t20210427_3693369.htm. (accessed on 2025/01/05)

76. Council S. Notice on Issuing the "14th Five-Year Plan" for National Aging Development and Elderly Care Service System [关于印发“十四五”国家老龄事业发展和养老服务体系规划的通知]. 2022. http://www.mwr.gov.cn/zw/zgzygwywj/202202/t20220221_1562452.html. (accessed on 2024/12/25)

77. NWCA MNCSC. Notice on Further Strengthening Barrier-Free Travel Services for the Elderly [关于进一步加强适老化无障碍出行服务工作的通知]. 2024. https://www.gov.cn/zhengce/zhengceku/202401/content_6925864.htm. (accessed on 2024/12/25)

78. CR MMNPNC. Notice on Effectively Solving the Difficulties of the Elderly in Using Smart Technologies and Facilitating Their Daily Travel [关于切实解决老年人运用智能技术困难便利老年人日常交通出行的通知]. 2020. https://xxgk.mot.gov.cn/2020/jigou/ysfws/202012/t20201228_3509294.html. (accessed on 2025/01/02)

79. MOT GOo. Notice on Issuing the Work Plan for 2021 to Facilitate Elderly People's Taxi Travel and Other 5 Closer-to-Life Practical Matters [关于印发 2021 年便利老年人打车出行等 5 件更贴近民生实事工作方案的通知]. 2021. http://jtt.hunan.gov.cn/jtt/xxgk/zcfg/zcfg_1/202104/t20210401_15427315.html. (accessed on 2025/01/02)

80. MOT GOo. Notice on Issuing the Work Plan for 2022 to Promote Elderly-Friendly Travel Services and Other 5 Closer-to-Life Practical Matters [关于印发 2022 年推行适老化交通出行服务等 5 件更贴近民生实事工作方案的通知]. 2022. https://view.officeapps.live.com/op/view.aspx?src=https%3A%2F%2Fxxgk.mot.gov.cn%2F2020%2Fjigou%2Fysfws%2F202204%2FP020220411350875902462.doc&wdOrigin=BROWSELINK. (accessed on 2025/01/02)

81. MOT GOo. Notice on Issuing the Work Plan for 2023 to Continuously Improve Elderly-Friendly and Barrier-Free Travel Services and Other 5 Closer-to-Life Practical Matters [关于印发 2023 年持续提升适老化无障碍交通出行服务等 5 件更贴近民生实事工作方案的通知]. 2023.

https://www.gov.cn/zhengce/zhengceku/2023-04/17/content_5751880.htm. (accessed on 2025/01/06)
82. MOT GOo. Notice on Issuing the Work Plan for 2024 to Expand, Improve, and Enhance Elderly-Friendly and Barrier-Free Travel Services and Other 5 Closer-to-Life Practical Matters [关于印发 2024 年适老化无障碍交通出行服务扩面提质增效等 5 件民生实事工作方案的通知]. 2024. https://www.gov.cn/zhengce/zhengceku/202404/content_6943595.htm. (accessed on 2025/01/06)
83. NEA MNMMMMCC. Notice on Issuing the Implementation Plan for Science and Technology to Support Carbon Peaking and Carbon Neutrality (2022-2030) [关于印发《科技支撑碳达峰碳中和实施方案（2022—2030 年）》的通知]. 2022. https://www.gov.cn/zhengce/zhengceku/2022-08/18/5705865/files/94318119b8464e2583a3d4284df9c855.pdf. (accessed on 2025/01/07)
84. Department of Aging Health N. Pilot Work on Remote Collaborative Services for Aging Health and Medical Care Integration Launched [老龄健康医养结合远程协同服务试点工作启动]. 2020. http://www.nhc.gov.cn/lljks/s7786/202007/dbbbd42cdf7b4ef1ae49b62649c29044.shtml.
85. MOF M. Notice on Organizing Central Financial Support for Centralized Care Services for Economically Disabled Elderly [关于组织开展中央财政支持经济困难失能老年人集中照护服务工作的通知]. 2023. https://www.gov.cn/zhengce/zhengceku/202310/content_6910517.htm. (accessed on 2024/12/30)
86. CBIRC MMS. Opinions on Strengthening the Prevention and Resolution of Illegal Fundraising in Elderly Care Institutions [关于加强养老机构非法集资防范化解工作的意见]. 2022. https://www.gov.cn/zhengce/zhengceku/2022-11/30/content_5729636.htm. (accessed on 2025/01/11)
87. SAMR MNMMMMMMMS. Notice on Issuing the Action Plan for Actively Developing Elderly Meal Assistance Services [关于印发《积极发展老年助餐服务行动方案》的通知]. 2023. https://www.mem.gov.cn/gk/zfxxgkpt/fdzdgknr/202311/t20231114_468483.shtml. (accessed on 2024/12/25)
88. NWCA MNMMNCLC. Guiding Opinions on Accelerating the Implementation of Home Adaptation Projects for the Elderly [关于加快实施老年人居家适老化改造工程的指导意见]. 2020. https://www.gov.cn/zhengce/zhengceku/2020-07/16/content_5527260.htm. (accessed on 2025/01/07)
89. NFRA MNMMPS. Guiding Opinions on Strengthening the Supervision of Pre-Charges in Elderly Care Institutions [关于加强养老机构预收费监管的指导意见]. 2024. https://www.gov.cn/gongbao/2024/issue_11386/202406/content_6955752.html. (accessed on 2024/12/25)
90. NWCA MNMMMMMMMNSS. Opinions on Strengthening the Construction of Elderly Care Service Talent Teams [关于加强养老服务人才队伍建设的意见]. 2023. https://www.gov.cn/zhengce/zhengceku/202401/content_6929136.htm. (accessed on 2024/12/25)
91. NDA M. Notice on Organizing Pilot Projects for the Comprehensive Platform for Basic Elderly Care Services [关于组织开展基本养老服务综合平台试点的通知]. 2024. https://www.gov.cn/zhengce/zhengceku/202402/content_6931233.htm. (accessed on 2025/01/10)
92. SAMR M. Opinions on Strengthening Food Safety Management in Elderly Care Services [关于强化养老服务领域食品安全管理的意见]. 2021. https://files.dandong.gov.cn/files/ueditor/ZAQZF/jsp/upload/file/20211230/1640845225361087620.pdf. (accessed on 2025/01/12)
93. MCA. Guiding Opinions on Giving Play to the Role of Grassroots Mass Autonomous Organizations to Strengthen Flood Control and Disaster Relief in Urban and Rural Communities [关于发挥基层群众性自治组织作用加强城乡社区防汛救灾工作的指导意见]. 2020. https://www.huadu.gov.cn/attachment/7/7667/7667424/9880138.pdf. (accessed on 2025/01/12)
94. CDPF; MCMNMMMMMMNMPSNSNSNA. Guiding Opinions on Accelerating the Development of Rural Elderly Care Services [关于加快发展农村养老服务的指导意见]. 2024. https://www.gov.cn/gongbao/2024/issue_11506/202408/content_6966480.html. (accessed on 2024/12/25)

95. NWCA MCCMMMMNC. Guiding Opinions on Carrying out Visiting and Caring Services for Special Difficult Elderly [关于开展特殊困难老年人探访关爱服务的指导意见]. 2022. https://www.gov.cn/gongbao/content/2022/content_5729427.htm. (accessed on 2024/12/25)

96. MARA MCCNMMMM. Opinions on Deepening the Construction of Smart Communities [《关于深入推进智慧社区建设的意见》]. 2022. https://www.gov.cn/zhengce/zhengceku/2022-05/21/content_5691593.htm. (accessed on 2025/01/13)

97. MCA. Notice on Implementing the Implementation Plan to Effectively Solve the Difficulties of the Elderly in Using Smart Technologies [民政部关于落实《关于切实解决老年人运用智能技术困难的实施方案》的通知]. 2020. https://www.gov.cn/zhengce/zhengceku/2020-12/30/content_5575177.htm. (accessed on 2024/12/30)

98. MCA. Ministry of Civil Affairs Deploys the Establishment of a National Unified Elderly Care Institution Rating System [民政部部署建立全国统一养老机构等级评定体系]. 2019. https://www.gov.cn/zhengce/zhengceku/2020-01/02/content_5465988.htm#:~:text=%E4%B8%BA%E6%B7%B1%E5%85%A5%E8%B4%AF%E5%BD%BB%E8%90%BD%E5%AE%9E%E4%B9%A0,%E5%87%BA%E4%BB%A5%E4%B8%8B%E6%8C%87%E5%AF%BC%E6%84%8F%E8%A7%81%E3%80%82. (accessed on 2025/01/12)

99. CDPF MMM. Notice on Promoting the Home Adaptation for Special Difficult Elderly Families during the "14th Five-Year Plan" Period [《关于推进“十四五”特殊困难老年人家庭适老化改造工作的通知》]. 2022. https://www.mca.gov.cn/n152/n167/c53551/content.html. (accessed on 2024/12/30)

100. MARA. Notice on Grasping the Rectification of Problems Found in the Major Inspection and Solidly Promoting the Improvement of Rural Living Environment [关于抓好大检查发现问题整改扎实推进农村人居环境整治的通知]. 2020. http://www.moa.gov.cn/nybgb/2020/202004/202005/t20200507_6343268.htm. (accessed on 2024/12/29)

101. NWCA. Notice on Carrying out the "Smart Assistance for the Elderly" Action [关于开展“智慧助老”行动的通知]. 2020. http://www.nhc.gov.cn/lljks/pqt/202012/3e8b6ac9653f4d2193ba09cba8ea8116.shtml. (accessed on 2025/01/08)

102. NWCA. Notice on Carrying out the Action to Improve Nutrition for the Elderly [关于开展老年营养改善行动的通知]. 2022. http://www.nhc.gov.cn/lljks/tggg/202210/013f52aae25c4bec9abcb773c57218e1.shtml. (accessed on 2025/01/08)

103. NWCCA. Notice on Issuing the Task Division Plan for Implementing the Opinions of the Central Committee of the Communist Party of China and the State Council on Strengthening Aging Work in the New Era [关于印发贯彻落实《中共中央 国务院关于加强新时代老龄工作的意见》任务分工方案的通知]. 2022. http://www.nhc.gov.cn/lljks/tggg/202202/ac784bab5b5c49d0957990d8051aa040.shtml. (accessed on 2025/01/08)

104. MCA. Guiding Opinions on Deepening the "Silver Age Action" in the New Era [关于深入开展新时代“银龄行动”的指导意见]. 2024. https://www.mca.gov.cn/n152/n165/c1662004999980001540/content.html. (accessed on 2024/12/30)

105. CNTA. Notice on Implementing the Implementation Plan to Effectively Solve the Difficulties of the Elderly in Using Smart Technologies 关于落实[《关于切实解决老年人运用智能技术困难的实施方案》的通知]. 2020. https://www.mct.gov.cn/whzx/bnsj/ggwhs/202012/t20201229_920306.html. (accessed on 2025/01/04)

106. MEE. Ecological Environment and Health Literacy of Chinese Citizens [《中国公民生态环境与健康素养》]. 2020. https://www.mee.gov.cn/xxgk2018/xxgk/xxgk01/202007/W020200727581542944802.pdf. (accessed on 2025/01/06)

107. MEE. Guiding Opinions on Coordinating and Strengthening Climate Change Response and Ecological Environmental Protection [关于统筹和加强应对气候变化与生态环境保护相关工作的指导意见]. 2021. https://www.mee.gov.cn/xxgk2018/xxgk/xxgk03/202101/t20210113_817221.html. (accessed on 2024/12/25)

108. NRA MNMMMMMCN. Notice on Issuing the Special Promotion Plan for Strengthening Rural Flood Control, Drought Relief, and Water Supply Guarantee [关于印发强化农村防汛抗旱和供水保障专项推进方案的通知]. 2022. http://www.mwr.gov.cn/zwgk/gknr/202210/t20221011_1600044.html. (accessed on 2024/12/25)

109. NHSA NNMMMMMMMS. Guiding Opinions on Further Promoting the Development of Medical and Elderly Care

Integration [关于进一步推进医养结合发展的指导意见]. 2022. https://www.gov.cn/gongbao/content/2022/content_5713989.htm. (accessed on 2025/01/06)

110. NFGA MNMMMMMMMNMC. Notice on Issuing the National Climate Change Health Adaptation Action Plan (2024-2030) [关于印发《国家气候变化健康适应行动方案（2024—2030年）》的通知]. 2024. https://www.ndcpa.gov.cn/jbkzzx/c100081/common/content/content_1836297186892951552.html. (accessed on 2025/01/06)

111. MEM GOo. Notice on Issuing the Reference for Compiling Emergency Response Plans for Townships (Streets) and Villages (Communities) [关于印发《乡镇（街道） 突发事件应急预案编制参考》和《村（社区） 突发事件应急预案编制参考》的通知]. 2023. https://www.mem.gov.cn/gk/zfxxgkpt/fdzdgknr/202308/t20230825_460298.shtml. (accessed on 2024/12/25)

112. Council S. Opinions on Strengthening Aging Work in the New Era [关于加强新时代老龄工作的意见]. 2021. https://www.gov.cn/gongbao/content/2021/content_5659511.htm. (accessed on 2024/12/25)

113. MOF. Opinions on Fully, Accurately, and Comprehensively Implementing the New Development Concept to Do a Good Job in Carbon Peaking and Carbon Neutrality [关于完整准确全面贯彻新发展理念做好碳达峰碳中和工作的意见]. 2021. https://nb.mof.gov.cn/czjg/dcyj/202111/t20211123_3767992.htm. (accessed on 2025/01/05/12:18:32)

114. MOF. Opinions on Promoting New Urban Infrastructure Construction to Build Resilient Cities [关于推进新型城市基础设施建设打造韧性城市的意见]. 2024. https://nmg.mof.gov.cn/jgdj2019/202412/t20241206_3949153.htm. (accessed on 2025/01/05)

115. Council CGOotS. Opinions on Promoting Green Development in Urban and Rural Construction [《关于推动城乡建设绿色发展的意见》]. 2021. https://www.gov.cn/zhengce/2021-10/21/content_5644083.htm. (accessed on 2025/01/07)

116. Council S. Opinions on Promoting the Construction of Basic Elderly Care Service System [《关于推进基本养老服务体系建设的意见》]. 2023. https://www.gov.cn/gongbao/2023/issue_10506/202306/content_6885267.html. (accessed on 2024/12/25)

117. MOF. Opinions on Accelerating the Comprehensive Green Transformation of Economic and Social Development [关于加快经济社会发展全面绿色转型的意见]. 2024. http://jx.mof.gov.cn/zt/jgdj/xxjl/202408/t20240812_3941568.htm. (accessed on 2025/01/07)

118. CBIRC GOo. Notice on Banking and Insurance Institutions Effectively Solving the Difficulties of the Elderly in Using Smart Technologies [关于银行保险机构切实解决老年人运用智能技术困难的通知]. 2021. https://www.gov.cn/zhengce/zhengceku/2021-03/31/content_5596890.htm. (accessed on 2024/12/25)

119. NWCA MNMNN. Opinions on Promoting Property Service Enterprises to Develop Home and Community Elderly Care Services [关于推动物业服务 企业发展居家社区养老服务的意见]. 2020. https://www.gov.cn/gongbao/content/2021/content_5581077.htm. (accessed on 2025/01/07)

120. MOHURD GOo. Notice on Issuing the Guidelines for Pocket Park Construction (Trial) [关于印发《口袋公园建设指南（试行）》的通知]. 2024. https://view.officeapps.live.com/op/view.aspx?src=https%3A%2F%2Fwww.gov.cn%2Fzhengce%2Fzhengceku%2F202406%2FP020240628447049143139.docx&wdOrigin=BROWSELINK. (accessed on 2025/01/07)

121. MOHURD GOo. Notice on Issuing the List of Replicable Policy Mechanisms for Sponge City Construction [关于印发海绵城市建设可复制政策机制清单的通知]. 2024. https://www.gov.cn/zhengce/zhengceku/202405/P020240517455123418322.pdf. (accessed on 2025/01/07)

122. MOHURD. Guiding Opinions on Comprehensively Carrying out Urban Physical Examination Work [关于全面开展城市体检工作指导意见]. 2023. https://www.gov.cn/zhengce/zhengceku/202312/content_6918801.htm. (accessed on 2025/01/07)

123. NDRC M. Notice on Issuing the "14th Five-Year Plan" National Urban Infrastructure Construction Plan [关于印发“十四五”全国城市基础设施建设规划的通知]. 2022.

https://www.gov.cn/zhengce/zhengceku/2022-07/31/5703690/files/d4ebd608827e41138701d06fe6133cdb.pdf. (accessed on 2025/01/07)

124. MWR MMN. Notice on Issuing the Implementation Plan for Deepening the Battle against Urban Black and Odorous Water Bodies [关于印发深入打好城市黑臭水体治理攻坚战实施方案的通知]. 2022. https://www.mee.gov.cn/xxgk2018/xxgk/xxgk10/202207/W020220717804650388908.pdf. (accessed on 2024/12/26)

125. CMA GOoMGOoNOo. Notice on Further Regulating the Release of Urban Waterlogging Prevention Information and Related Work [关于进一步规范城市内涝防治信息发布等有关工作的通知]. 2022. https://www.gov.cn/zhengce/zhengceku/2022-07/16/content_5701336.htm. (accessed on 2025/01/07)

126. NDRC GOoMGOo. Notice on Doing a Good Job in Urban Drainage and Waterlogging Prevention in 2023 [关于做好2023年城市排水防涝工作的通知]. 2023. https://www.gov.cn/zhengce/zhengceku/2023-04/20/content_5752356.htm. (accessed on 2025/01/07)

127. LGOP GOoMCDo. Notice on Doing a Good Job in Ensuring the Housing Safety of Poor Farmers Affected by Floods and Geological Disasters [关于做好因洪涝地质灾害影响贫困农户住房安全保障工作的通知]. 2020. https://www.gov.cn/zhengce/zhengceku/2020-08/21/content_5536492.htm. (accessed on 2025/01/07)

128. MOHURD GOo. Notice on Further Clarifying the Requirements for Sponge City Construction Work [关于进一步明确海绵城市建设工作有关要求的通知]. 2022. https://www.gov.cn/zhengce/zhengceku/2022-04/29/content_5687999.htm. (accessed on 2025/01/07)

129. MOHURD GOo. Notice on Carrying out Pilot Work on Open Sharing of Urban Park Green Spaces [关于开展城市公园绿地开放共享试点工作的通知]. 2023. https://www.gov.cn/zhengce/zhengceku/2023-02/06/content_5740376.htm. (accessed on 2025/01/07)

130. MOHURD GOo. Notice on Carrying out Pilot Work on Urban Landscaping Waste Treatment and Resource Utilization [关于开展城市园林绿化垃圾处理和资源化利用试点工作的通知]. 2022. https://www.gov.cn/zhengce/zhengceku/2022-11/07/content_5725223.htm. (accessed on 2025/01/07)

131. MOHURD GOo. Notice on Promoting the Construction of "Pocket Parks" [关于推动“口袋公园”建设的通知]. 2022. https://www.gov.cn/zhengce/zhengceku/2022-08/09/content_5704766.htm. (accessed on 2025/01/07)

132. MOHURD GOo. Notice on Issuing the Guidelines for Complete Residential Community Construction [关于印发完整居住社区建设指南的通知]. 2021. https://www.gov.cn/zhengce/zhengceku/2022-01/12/5667815/files/a84ca3d812e54074a43e332f3cc18eca.pdf. (accessed on 2025/01/07)

133. SAMR MNMMM. Notice on Issuing the Action Plan for Green Community Creation [关于印发绿色社区创建行动方案的通知]. 2023. https://view.officeapps.live.com/op/view.aspx?src=https%3A%2F%2Fwww.gov.cn%2Fzhengce%2Fzhengceku%2F2020-08%2F01%2F5531812%2Ffiles%2F1319995e9fe648d9a90438a6d27cd644.doc&wdOrigin=BROWSELINK. (accessed on 2025/01/07)

134. MOHURD. Notice on Issuing the Management Measures for the Application and Selection of National Garden Cities [关于印发国家园林城市申报与评选管理办法的通知]. 2022. https://www.gov.cn/zhengce/zhengceku/2022-01/14/content_5668177.htm. (accessed on 2025/01/07)

135. MOHURD. Ministry of Housing and Urban-Rural Development and State Council Leading Group Office of Poverty Alleviation and Development Jointly Issue Notice to Ensure the Housing Safety of Poor Farmers Affected by Floods and Geological Disasters [住房和城乡建设部国务院扶贫办联合下发通知要求 切实保障洪涝地质灾害受灾贫困农户住房安全]. 2020. https://www.gov.cn/xinwen/2020-08/24/content_5536967.htm.

136. SPB MNMMMMMMNSNG. Three-year Action Plan to Comprehensively promote the Construction of Urban one-quarter Hour Convenient Living Circle (2023-2025)[关于印发《全面推进城市一刻钟便民生活圈建设三年行动计划(2023-2025)》的通知]. 2023. https://www.gov.cn/zhengce/zhengceku/202307/content_6891466.htm. (accessed on

2025/01/05)

137. Council S. The Five-Year Action Plan for the In-depth Implementation of the People-Centered New Urbanization Strategy[关于印发《深入实施以人为本的新型城镇化战略五年行动计划》的通知]. 2024. https://www.gov.cn/zhengce/content/202407/content_6965542.htm. (accessed on 2025/01/06)

138. MOHURD. 14th Five-Year Plan for Building Energy Efficiency and Green Building Development [关于印发“十四五”建筑节能与绿色建筑发展规划的通知]. 2022. https://view.officeapps.live.com/op/view.aspx?src=https%3A%2F%2Fwww.gov.cn%2Fzhengce%2Fzhengceku%2F2022-03%2F12%2F5678698%2Ffiles%2Fc6b07b1ddd944810a2800bf20db0c128.doc&wdOrigin=BROWSELINK. (accessed on 2024/12/26)

139. Council S. Guiding Opinions on Actively and Steadily Promoting the Construction of Public Infrastructure for Both Peacetime and Emergency Use in Megacities [关于积极稳步推进超大特大城市“平急两用”公共基础设施建设的指导意见]. 2023. https://www.gov.cn/lianbo/bumen/202407/content_6960782.htm. (accessed on 2024/12/28)

140. NCA NMMMN. Notice on Addressing Shortcomings in Public Health Environmental Facilities and Carrying Out Urban and Rural Environmental Sanitation Cleanup and Improvement[关于补齐公共卫生环境设施短板 开展城乡环境卫生清理整治的通知]. 2023. https://www.ndrc.gov.cn/xwdt/tzgg/202308/t20230824_1360049.html. (accessed on 2025/01/04)
